# An invariant-guided model for automated detection of anomalous execution regimes from production telemetry

**DOI:** 10.1371/journal.pone.0362583

**Published:** 2026-11-12

**Authors:** Viacheslav Kovtun, Frederic Huralnyk, Artem Huralnyk, Marta Yankiv, Jamil Abedalrahim Jamil Alsayaydeh

**Affiliations:** 1 Vinnytsia National Technical University, Vinnytsia, Ukraine; 2 Ivan Franko National University of Lviv, Lviv, Ukraine; 3 Vinnytsia trade economics university, Vinnytsia, Ukraine; 4 Universiti Teknikal Malaysia Melaka, Durian Tunggal (UTeM), Melaka, Malaysia; Philadelphia University, JORDAN

## Abstract

Modern cloud-based distributed software systems increasingly exhibit persistent changes in execution behaviour that cannot be adequately characterised by isolated events or threshold violations alone. Although contemporary production environments already provide mature observability infrastructures for collecting logs, metrics, and traces, the formal representation of execution regimes derived from such telemetry remains insufficiently developed. This paper proposes an invariant-guided geometric framework for modelling execution regimes and detecting anomalous regime transitions under weak supervision. The framework applies common principles of invariant-based feature mapping, weighted pseudometrics, and geometric regularisation to log-derived and KPI-derived regime representations. Separate evaluations of these modality-specific implementations on HDFS logs and operational KPI telemetry demonstrate consistent improvements over representative baseline methods under limited supervision while preserving an explicit and interpretable execution-regime representation. Joint evaluation using synchronized logs and metrics remains future work. The results support the framework as a mathematical analytical layer for execution-regime analysis that complements existing production observability infrastructures.

## 1 Introduction

### 1.1 Relevance and problem context

Modern cloud-hosted, distributed, and microservice-based software systems are characterised by increasing architectural complexity, continuous evolution, and dense inter-service coupling [1–3]. Runtime behaviour in such systems is determined not only by application logic, but also by deployment configuration, orchestration policies, infrastructure state, workload dynamics, and external dependencies. As a result, reliability, resilience, and observability have become central requirements for the operation of contemporary software platforms rather than auxiliary engineering concerns [4,5].

Modern cloud-native production environments already rely on mature observability, orchestration, scheduling, autoscaling, and infrastructure-management tooling. Such systems support telemetry acquisition, aggregation, visualisation, alerting, resource control, and operational automation. The present study does not question or replace these production mechanisms. Instead, it addresses a different analytical problem: how heterogeneous telemetry produced by existing observability infrastructures can be transformed into an explicit mathematical representation of persistent execution regimes. In this sense, the proposed framework should be interpreted as an invariant-guided analytical layer above existing observability and operational-management stacks rather than as an alternative monitoring, scheduling, or DCIM platform.

Failures in such environments are rarely limited to a single isolated component. Small configuration inconsistencies, resource imbalances, delayed service responses, unstable dependencies, or workload fluctuations may interact and gradually alter the behaviour of the entire system. Incident-response and cyber-crisis-management studies emphasise that operational disruptions often arise from chains of interacting technical and organisational factors rather than from a single directly observable cause [6–8]. In this sense, the operationally significant object is not only the individual fault event, but also the behavioural configuration in which the system enters and remains during degradation.

In this study, such persistent behavioural configurations are interpreted as execution regimes. An execution regime is understood as a relatively stable, time-bounded pattern of system functioning that is reflected in coordinated changes of operational observations. An anomalous execution regime is therefore not necessarily associated with an immediate crash or a single threshold violation. It may instead appear as gradual performance degradation, intermittent service-level instability, abnormal resource-use patterns, or changes in the relation between logged events and metric signals. This interpretation shifts the analytical focus from isolated anomalies to structural transformations of runtime behaviour.

The empirical basis for observing these transformations is provided by logs, metrics, and execution traces [9,10,32–35]. Logs encode discrete and semantically rich event information. Metrics describe continuous or quasi-continuous resource, workload, and timing characteristics. Traces reflect execution paths and service interactions. These sources differ in sampling rate, structure, cardinality, and sensitivity to instrumentation or deployment changes. Consequently, monitoring approaches based only on isolated indicators, fixed thresholds, or event-level anomaly triggers may fail to capture deviations that emerge gradually across several telemetry channels.

The problem becomes particularly important in systems where reliability and operational stability depend on coordinated behaviour across interacting components. Persistent anomalous regimes may reflect changes in event composition, resource dynamics, or relationships expected under nominal execution. Such departures provide an operational target for detection even when their underlying cause has not yet been established. At the same time, the available labelled information is usually limited. Production environments contain many unlabelled regimes, while confirmed anomalous cases or expert-interpreted observations represent only a small fraction of observed behaviour [11,12]. This creates a need for methods that can operate under weak supervision and analyse system behaviour at the level of regimes rather than isolated observations.

The relevance of the present study therefore follows from the need to model software execution as a structured operational process. A regime-oriented formulation makes it possible to analyse coordinated changes in logs, metrics, and traces, to represent persistent deviations as transformations in a feature space, and to relate such transformations to invariant properties of correct execution. This problem formulation provides the foundation for the mathematical model developed in Sect 2 and for the experimental verification presented in Sect 3.

### 1.2 Analysis of state-of-the-art approaches

From the perspective of the mathematical object being modelled, contemporary approaches to anomaly detection in software execution can be divided into several paradigms. Rule-based and signature-based approaches [13,14] model explicitly specified conditions: an anomaly is detected when an observed event, state, or traffic pattern satisfies a predefined rule or signature. This gives such methods high interpretability, but also limits their applicability to known deviation types and requires continuous rule maintenance as the system evolves.

Classical statistical methods [15] model deviations from an assumed distribution or from locally estimated normal behaviour. They are computationally efficient and useful for short-lived or well-localised anomalies, but their performance depends strongly on aggregation rules, threshold design, distributional assumptions, and the stability of the monitored signal. In complex distributed systems, where deviations may emerge from the interaction of several weak signals, local statistical descriptions are often insufficient.

A broad group of representation-learning methods [16,17] replaces explicit rules or statistical assumptions with learned feature spaces. Such methods can capture complex nonlinear dependencies, but the resulting representations often lack direct operational semantics. Their reliability is also sensitive to data quality, feature stability, missing values, noise, and schema drift [18]. This is important for operational telemetry, where changes in log formats, metric labels, instrumentation, or deployment configuration can substantially alter detector behaviour. Empirical studies of robustness and evaluation protocols further show that reported performance may vary depending on noise level, dataset construction, train-test splitting, and labelling strategy [19,20].

A second major paradigm models execution as a sequence of events. DeepLog [21] represents normal behaviour through the predictability of short-range log-event sequences, whereas PLELog [22] addresses label scarcity by estimating probabilistic labels for log sequences. These methods remain methodologically relevant because they capture two central formulations of log anomaly detection: sequential prediction and semi-supervised probabilistic interpretation. Their limitation, however, is that the basic analytical object remains a log sequence or a probabilistic log-sequence label, not an execution regime as a structurally organised state-transition object.

Log parsing remains an essential part of this paradigm because it determines the representation on which subsequent detectors operate. Drain [23], for example, was designed as an online log parser based on a fixed-depth tree structure and remains important for scalable log preprocessing. However, parser choice can substantially affect the number of extracted templates, the dimensionality of the resulting representation, and the stability of downstream anomaly detection. This issue is particularly visible in large benchmark collections such as LogHub and LogHub-2.0. The latter contains 14 datasets with approximately 3.6 million log messages per dataset, which makes parser robustness a practical part of detector robustness rather than a separate preprocessing detail.

Since 2021, transformer-based and language-model approaches have shifted log anomaly detection from recurrent sequence modelling toward latent semantic representation. This line of work begins with masked-language-modelling formulations and then moves toward cross-domain adaptation and foundation-model-style log interpretation. BERT-Log [24] and LogBERT both transfer masked-language-modelling ideas to system logs; LogBERT combines masked log-message prediction with hypersphere-volume minimisation and evaluates the model on HDFS, BGL, and Thunderbird [25]. LAnoBERT reduces dependence on predefined log templates by applying BERT masked language modelling directly to system logs [26]. LogFormer extends this paradigm by combining transformer pre-training with adapter-based tuning for cross-domain log anomaly detection [27]. More recent language-model approaches, including LogGPT and LogFiT, use generative or fine-tuned language models to capture semantic regularities in logs [28,29]. These methods are important contemporary performance baselines, but their principal object is still a latent embedding, prediction score, or language-model output. The regime structure itself remains implicit.

Metric- and trace-oriented approaches form another related line of work. Studies on recurrent event data, time-series analysis, and cyber-security analytics [30,31] show that temporal structure, recurrence, and event dynamics are central for interpreting system behaviour. However, metric and trace analysis often remains focused on local temporal deviations, recurrent-event patterns, or attack-prediction settings rather than on the explicit construction of a unified execution-regime space.

Graph-based observability methods model another object: dependency structure. Instead of treating logs as isolated sequences, they represent services, traces, or execution entities as vertices and invocation, scheduling, dependency, or causal relations as edges. DeepTraLog combines trace and log information through trace-event graph modelling for microservice anomaly detection [32]. TraceVAE applies a dual-variable graph variational autoencoder to microservice traces, separating structural and temporal trace information [33]. MSTGAD constructs a microservice-system twin graph and combines logs, metrics, and traces through attentive multimodal learning [34]. LMGD similarly combines logs and metrics through graph-based deep learning [35]. These methods explicitly acknowledge service topology and multimodal dependencies, but anomaly detection is still typically performed through learned graph embeddings, reconstruction errors, or prediction scores.

For KPI-oriented AIOps, recent work has moved from manually configured detectors to AutoML and pre-trained time-series models. ADS [36] remains relevant as a representative KPI-oriented method for rapid deployment under limited labelling, but it no longer represents the full state of the field. AutoKAD formulates KPI anomaly detection as a label-free AutoML problem, combining algorithm selection and hyperparameter optimisation for large numbers of KPI streams; its evaluation includes the 2018 AIOps Challenge dataset with 29 KPI series from large Internet-company systems [37]. KAD-Disformer introduces a pre-trained disentangled transformer for KPI anomaly detection and addresses KPI heterogeneity and noisy data in large-scale online service systems [38]. These approaches are strong contemporary metric-based baselines, but they primarily optimise detection performance for KPI time series rather than constructing an explicit mathematical space of execution regimes.

A related diagnostic paradigm focuses not only on detection, but also on root-cause analysis. Chain-of-Event builds weighted event causal graphs from multimodal observation data and emphasises interpretability aligned with SRE practice [39]. Such methods are valuable for post-incident explanation, but their central task differs from the present study: they explain or localise abnormal behaviour after it has been observed, whereas the present work formalises execution regimes themselves as primary analytical objects.

Although transformer-based, graph-based and AutoML approaches substantially improve predictive performance, they do not change the fundamental analytical object of the problem. Their primary objective remains the estimation of anomaly likelihood from learned representations, whereas the explicit mathematical formalisation of execution regimes has received considerably less attention.

Thus, existing paradigms differ not only in algorithms, but in the mathematical objects they model. Rule-based methods model conditions; statistical methods model distributions; sequence-learning methods model event order; transformer and language-model approaches model latent semantic regularities; metric and trace methods model temporal deviations; graph-based approaches model dependency structures; KPI AutoML methods model detector selection and time-series behaviour. Unlike these paradigms, the mathematical object modelled in the present work is not an event, a sequence, a graph, or a latent embedding, but an execution regime equipped with explicitly defined feature mappings, weighted pseudometrics, invariant-violation functionals, geometric regularisation, and optimisation criteria. This formulation allows anomaly detection to be interpreted as a structural transformation within an execution-regime space rather than as a classification problem over latent representations. This paradigmatic comparison is summarised in Table 1.

**Table 1:** Paradigmatic comparison of approaches to anomalous execution regime detection.

Approach/paradigm	Primary mathematical object	Relation to execution regimes	Explicit analytical structure	Scalability	Key limitations
Rule-based/signature-based	Rules, signatures, predefined conditions	Absent; event- or condition-level analysis	Explicit rules and decision conditions	Limited in dynamic systems	Manual rule maintenance; weak generalisation; limited handling of emergent or combinatorial anomalies
Classical statistical methods	Distributions, thresholds, local deviations	Limited; local deviations rather than regime transitions	Distributional assumptions and deviation statistics	Moderate	Sensitive to aggregation, thresholds, parameter settings, and distributional assumptions; weak modelling of interdependencies
Sequential log models/DeepLog	Log-event sequences	Local; short-range event patterns	Next-event prediction and candidate-based decisions	Moderate	No global execution-regime space; anomalies treated as local sequence violations
Semi-supervised log models/PLELog	Probabilistic log-sequence representations	Limited; aggregation of sequence-level estimates	Probabilistic labels and sequence-classification scores	Moderate–high	Execution regimes are not first-class entities; no explicit regime representation
Transformer/language-model approaches	Learned latent semantic representations	Partial; regime structure remains implicit	Learned representations and model-dependent anomaly scores	Moderate–high, but computationally dependent	Strong predictive capacity, but limited operational transparency; regime transitions are not explicitly formalised
Graph-based observability	Dependency graphs and multimodal service topology	Partial; topology and dependencies explicit	Explicit topology with learned graph representations	Moderate–high	Anomaly decisions usually rely on learned embeddings, reconstruction errors, or prediction scores
KPI-oriented anomaly detection	KPI time-series dynamics and detector configuration	Limited; metric-level monitoring	Stream descriptors and detector-specific decision rules	High	Strong for KPI streams, but restricted to metrics; no explicit heterogeneous execution-regime space
**Proposed approach**	Execution-regime space with feature mappings, weighted pseudometrics, invariant functionals, and optimisation criteria	Explicit; regimes are primary analytical entities	Defined invariant violations, feature weights, distances, and regime prototypes	Suitable for offline batch analysis; production-scale computational performance not evaluated	Streaming adaptation and production-scale deployment are not evaluated in this study

As shown in Table 1, existing approaches differ not only in algorithmic design but also in the mathematical objects they model. Rule-based methods operate on predefined conditions, statistical methods on probability distributions, sequential models on event sequences, transformer and language-model approaches on learned latent representations, graph-based approaches on dependency structures, and KPI-oriented methods on metric time-series behaviour. Although recent transformer-based, graph-based, and AutoML approaches have substantially improved predictive performance, they do not fundamentally change the analytical object of anomaly detection: anomalies are still inferred from events, sequences, graphs, or latent representations rather than from explicitly modelled execution regimes. The proposed framework therefore operates at a different methodological level. Instead of estimating anomaly likelihood from implicit representations, it formalises execution regimes as explicit mathematical objects endowed with feature mappings, weighted pseudometrics, invariant functionals, and optimisation criteria. Consequently, anomalous behaviour is interpreted as a structural transformation within the execution-regime space rather than merely as a classification outcome. The analytical-structure column identifies quantities exposed by each formulation rather than ranking human interpretability. For the proposed framework, invariant violations, feature weights, distances, and regime prototypes provide inspectable components of the detector. Their usefulness for human diagnosis requires separate evaluation.

This distinction also determines the relationship between the proposed framework and existing production tooling. Observability platforms, schedulers, autoscaling mechanisms, DCIM systems, SIEM/SOAR tools, and AIOps pipelines solve operational tasks such as telemetry collection, infrastructure control, alert routing, visualisation, incident management, and remediation support. The proposed model does not duplicate these functions. It assumes that operational telemetry has already been collected and focuses on the formal representation, invariant-guided organisation, and geometric analysis of execution regimes derived from that telemetry.

### 1.3 Motivation and contributions of this study

Modern cloud-hosted, distributed, and microservice-oriented platforms increasingly exhibit failures that are better described as sustained changes in runtime behaviour than as single, clearly localised faults [1,3,6,8]. Such changes may appear as gradual performance degradation, intermittent service-level violations, shifts in request patterns, or changes in resource-consumption profiles, even when individual telemetry signals remain within locally acceptable ranges. In highly coupled and continuously evolving systems, relatively small deviations may accumulate and produce system-level behavioural changes that are difficult to attribute to a single component or event [4,5].

A further challenge is that the observational basis for diagnosing such behaviour is heterogeneous. Logs, metrics, and traces differ in structure, sampling rate, semantic content, and sensitivity to instrumentation or deployment changes [1,2,11]. As a result, anomaly detection based only on isolated indicators, predefined thresholds, or event-level triggers may fail to capture regime-level drift, especially when deviations emerge through interactions among several weak signals rather than through one dominant symptom [6–8]. This motivates the treatment of persistent deviations as execution regimes and their explicit modelling as structured objects in a regime space. Under this formulation, departures from nominal behaviour can be analysed through changes in regime geometry and invariant relationships, even under limited supervision and a shortage of annotated anomalies.

The object of this study is the execution of cloud-based distributed software systems as an operational process reflected in the dynamics of runtime telemetry under continuous deployment and inter-service coupling [1–3]. The empirical manifestations of this process are considered through logs and KPI-style signals collected in production or quasi-production environments. The study develops a regime-oriented representation of execution behaviour and addresses the corresponding detection task. Specifically, execution regimes are modelled as relatively stable, time-bounded patterns of system functioning, while anomalous regime shifts are interpreted as structured departures from nominal behaviour in the execution-regime space formalised in Sects 2–3.

The aim of this study is to develop an interpretable mathematical framework for modelling and detecting persistent behavioural deviations in cloud-based distributed systems. The framework represents execution regimes geometrically, aligns the representation with invariant structure, and supports the identification of anomalous regime shifts under limited supervision. The experimental part of the study evaluates this framework in an offline, semi-supervised setting on real operational datasets; it is intended to validate the proposed mathematical apparatus rather than to claim a complete online production-monitoring deployment.

The main contributions of the work are as follows:

1. An invariant-oriented geometric model of software-system execution regimes is proposed, in which regimes are formalised as structural transitions and treated as primary analytical objects.
2. A multilevel representation operator with weakly supervised calibration of weights is developed, providing a common formalism for constructing execution-regime features from log observations or metric observations without full-scale supervised training. Its log-based and metric-based implementations are evaluated separately on HDFS and AIOps.
3. A weighted pseudometric together with invariant-geometric regularisation is introduced to support stable spatial organisation of execution regimes in a heterogeneous observation space.
4. An offline semi-supervised validation protocol is provided, showing that regime-oriented clustering can identify anomalous execution regimes under limited supervision on real operational data. Component ablations evaluate invariant regularisation, geometric regularisation, and learned metric weights under a common base representation, using both shared configurations and development-based retuning.

The article is structured as follows. Sect 2 formalises the research problem and introduces the invariant-oriented geometric model of software-system execution regimes, including the construction of the feature space, weighted similarity measures, invariant regularisation, and the hierarchical quality functional. Sect 3 presents the operational instantiation and experimental verification of the approach on real datasets in a semi-supervised offline setting, together with quantitative evaluation and ablation analysis. Sect 4 summarises the results, discusses practical implications and limitations, and outlines directions for online adaptation and production-oriented validation.

## 2 Models and methods

### 2.1 Research statement

Consider a software system P, whose behaviour during operation is described by a sequence of execution states ${\left\{S_{r}\right\}}_{r=1}^{N}$, $S_{r}\in S$, where S denotes the set of admissible execution states defined by execution invariants as a collection of nominal execution regimes. In this study, these invariants are used as a mathematical reference for analysing operational behaviour rather than as a direct representation of security states. Invariant violations are not interpreted as an immediate exit beyond S, but are instead regarded as indicators of a potentially anomalous regime that requires further analysis. Such violations indicate deviations from nominal execution behaviour, but they are not treated as direct evidence of software faults, security incidents, or malicious activity without additional operational context.

Unlike abstract models, under real operational conditions each state $S_{r}$ is not observed directly, but is reconstructed indirectly through operational data generated during software execution. Such data include event logs and numerical metrics, which capture both the semantics of executed actions and quantitative characteristics of system operation. In this study, an anomalous execution regime is understood as a transition of the software system into a state characterised by structurally consistent deviations in the available operational observations from the invariants of nominal behaviour. These deviations provide evidence of abnormal execution and may support subsequent reliability assessment and operational diagnosis. The present evaluation concerns detection of operational deviations. Attribution to a software fault, a workload change, a configuration problem, or malicious activity requires additional contextual evidence and cause-specific ground truth

The correspondence between execution states and observable data is established by means of an operator F:S→P(O), which associates each state $S_{r}$ with an empirical set of logs and metric measurements. In practical implementation, $F\left(S_{r}\right)$ corresponds to an ordered collection of log events and metric observations gathered within a finite time window associated with the system remaining in state $S_{r}$. Thus, a transition between two successive execution states $\delta_{r}:S_{r-1}\to S_{r}$ induces a change in the observation space, which is described by the operator $\Delta_{r}=F\left(S_{r}\right)⊖F\left(S_{r-1}\right)$, where ⊖ is interpreted as a generalised operation of structural difference. This operation encompasses both the emergence or disappearance of log events and changes in the character of metric signals, and serves as the basic object of further analysis. In the general case, the operation ⊖ is not symmetric and reflects a directed structural change arising from the transition between states, preserving information about the appearance, disappearance, or transformation of events and metric patterns.

### 2.2 Invariant-guided geometric model for detection of anomalous execution regimes

The direct use of objects $\Delta_{r}\in P\left(O\right)$ in machine learning procedures is not feasible due to their structural complexity and variable dimensionality. This necessitates the construction of a mapping from the space of observation changes into a finite-dimensional vector space. For this purpose, a multilevel representation operator $\Phi =\Phi^{\left(3\right)}\circ \Phi^{\left(2\right)}\circ \Phi^{\left(1\right)}$ is introduced, which implements a sequential transformation of operational data (from elementary changes to a stable numerical representation suitable for geometric analysis).

At the first stage, each change $\Delta_{r}$ is decomposed into components $\Delta_{r}=\left(\Delta_{r}^{L},\Delta_{r}^{M}\right)$, where $\Delta_{r}^{L}$ contains modified log events and $\Delta_{r}^{M}$ contains modified fragments of metric signals. For each elementary object $x\in \Delta_{r}^{L}$ or $y\in \Delta_{r}^{M}$, a family of local functions $l_{i}:O_{L}\to \mathbb{R}$, $m_{j}:O_{M}\to \mathbb{R}$ is defined, which encode structural, frequency-based, temporal, or semantic characteristics of the changes. In particular, the functions $l_{i}$ may reflect frequency-related, sequential, or semantic characteristics of log events, whereas $m_{j}$ describe local properties of metric signals, including amplitude-based, temporal, or correlation changes over the corresponding intervals. At this level, no generalisation is performed – only local properties are recorded, reflecting the “microscopic” dynamics of system behaviour.

For reproducibility, each local function is defined as a deterministic local mapping from an elementary observation to a scalar characteristic. Formally, $l_{i}:\Delta_{r}^{L}\to \mathbb{R}$ and $m_{j}:\Delta_{r}^{M}\to \mathbb{R}$ belong to the class of admissible local operators satisfying the following conditions: (i) determinism, i.e., identical input observations always produce identical output values; (ii) locality, i.e., the function depends exclusively on the current observation and the predefined analysis window; (iii) scalarity, i.e., each operator produces a single real-valued characteristic; and (iv) computability, i.e., the operator is evaluated directly from the available telemetry without requiring hidden variables or subjective expert intervention. Consequently, the mathematical framework is independent of any particular feature engineering strategy. Any finite collection of operators satisfying these conditions constitutes a valid instantiation of the proposed model, whereas the specific operator set employed in the experimental evaluation is described separately in Sect 3 to ensure full reproducibility of the reported results.

The subsequent step consists in aggregating local estimates into intermediate generalised characteristics. For this purpose, the mapping


$$\begin{array}{c} \Phi_{k}^{\left(2\right)}\left(\Delta_{r}\right)=\sum_{x\in \Delta_{r}^{L}}\alpha_{k}\left(x\right)l_{k}\left(x\right)+\int_{\tau \in \Delta_{r}^{M}}\beta_{k}\left(\tau \right)m_{k}\left(\tau \right)d\tau , \end{array}$$ (1)

is used, in which the weighting functions $\alpha_{k}$ and $\beta_{k}$ play a key role. They make it possible to account for the context of event occurrence, the source of logs, temporal scales of metric changes, and other factors that cannot be captured at the local level. It is precisely at this stage that the possibility of weakly supervised calibration arises, whereby the weights are refined using a limited number of expert-interpreted incidents or historical data on known types of failures. Interval incident indicators, historical failure labels, or aggregated signals of incorrect operation without precise localisation of the cause may be used as weak labels.

Operationally, the weighting functions $\alpha_{k}$ and $\beta_{k}$ are defined as deterministic non-negative weighting operators evaluated within the same analysis window as the corresponding local functions. The operator $\alpha_{k}$ assigns a scalar contribution weight to each log observation $x\in \Delta_{r}^{L}$ in the k-th aggregated characteristic, whereas $\beta_{k}$ assigns a scalar contribution weight to each metric observation used in the corresponding metric component. Consequently, the aggregation in (1) is fully determined by the specified local functions and weighting operators and does not rely on implicit or undefined computational components. The present formulation defines the admissible mathematical class of weighting operators used in the proposed framework, while the concrete weighting scheme employed in the experimental evaluation is reported in Sect 3.

The result of aggregation is a system of generalised characteristics, which are subsequently subjected to nonlinear embedding into a feature space:


$$\begin{array}{c} \mu_{\delta_{r}}=\Phi \left(\Delta_{r}\right)=\left(\sigma_{\text{1}}\left(\Phi_{\text{1}}^{\left(2\right)}\left(\Delta_{r}\right)\right),\text{\textbackslash{}ldots},\sigma_{p}\left(\Phi_{p}^{\left(2\right)}\left(\Delta_{r}\right)\right)\right), \end{array}$$ (2)

where the nonlinear mappings $\sigma_{i}$ (in particular, robust standardisation, logarithmic scaling, or smoothing nonlinearities) ensure the stability of the representation with respect to scale, noise, and anomalous outliers. Such a construction makes it possible to treat each change of execution state $\delta_{r}$ as a point $\mu_{\delta_{r}}\in {\mathbb{R}}^{p}$ in a fixed vector space, the dimensionality of which is determined by the composition of the feature space.

The collection of all constructed vectors $M=\left\{\mu_{\delta_{\text{1}}},\text{\textbackslash{}ldots},\mu_{\delta_{N}}\right\}$ forms an empirical geometric representation of the software system’s behaviour. To analyse the mutual arrangement of such points in the feature space, a weighted pseudometric structure $d_{w}\left(\mu_{a},\mu_{b}\right)={\left(\sum_{i=1}^{p}w_{i}{\left|\mu_{a}^{\left(i\right)}-\mu_{b}^{\left(i\right)}\right|}^{q}\right)}^{1/q}$ is introduced, where the weights $w_{i}$ reflect the correspondence of individual features to system invariants, and the parameter q determines the sensitivity of the metric to local and global deviations. The parameter q may be fixed or subject to calibration, while the pseudometric $d_{w}$ allows for a violation of the identity of indiscernibles, which is acceptable in the context of aggregated and noisy features. The specified weights may be set expert-wise, refined in a weakly supervised regime, or optimised jointly with a quality criterion.

The evaluation of the constructed feature space is carried out using a hierarchical functional


$$\begin{array}{c} I\left(M\right)=\sum_{c\in C}\gamma_{c}I_{c}\left(M\right)+\lambda_{\text{1}}R_{geom}\left(M\right)+\lambda_{\text{2}}R_{inv}\left(M\right), \end{array}$$ (3)

which combines cluster criteria of compactness and separability with regularisation components that control the geometry of the space and compliance with invariants. In this formulation, the construction of feature vectors and the choice of their geometry are directly integrated with the subsequent clustering task, which takes the form of an optimisation problem for the functional *I*.

The introduced hierarchical functional I(M) serves as a generalised quality criterion for the geometric representation M and its cluster structure, where the set C is interpreted in this context as a set of quality components. Each component $I_{c}\left(M\right)$ in (3) corresponds to a particular aspect of cluster partition quality or representation, such as intra-cluster compactness, inter-cluster separability, partition stability, or consistency with a priori weak labels. In practical experiments, it is appropriate to include measures of intra-cluster compactness, inter-cluster separability, and consistency with weakly supervised labels in the set $I_{c}$. The weighting coefficients $\gamma_{c}\geq 0$ determine the relative importance of the corresponding quality aspects, while the parameters $\lambda_{\text{1}}\geq 0$, $\lambda_{\text{2}}\geq 0$ regulate the contribution of regularisation components that control the geometric properties of the feature space and conformity with execution invariants.

To ensure the computability of the functional (3), it is appropriate to specify $\gamma_{c}$, $\lambda_{\text{1}}$, $\lambda_{\text{2}}$ not as arbitrary constants, but as parameters subject to calibration on a limited set of control situations (weakly supervised), or as parameters selected by an expert with due regard to the domain-specific importance of the corresponding criteria. In the general case, it is convenient to introduce normalisation $\sum_{c\in C}\gamma_{c}=1$ in order to interpret $\gamma_{c}$ as composition weights, whereas $\lambda_{\text{1}}$, $\lambda_{\text{2}}$ play the role of regularisation coefficients (analogous to Lagrange multipliers in problems with soft constraints).

The content of the regularisation component $R_{geom}$ consists in controlling the “convenience” of the geometry of the feature space for subsequent clustering. Since $M=\left\{\mu_{\delta_{\text{1}}},\text{\textbackslash{}ldots},\mu_{\delta_{N}}\right\}\subset {\mathbb{R}}^{p}$ is given and a weighted pseudometric $d_{w}$ is defined on it, geometric regularisation should be linked to the properties of pairwise distances and local neighbourhood structure. Let $D_{ij}=d_{w}\left(\mu_{\delta_{i}},\mu_{\delta_{j}}\right)$, 1≤i,j≤N, and for each point $\mu_{\delta_{i}}$ let the set of its $k_{nn}$ nearest neighbours $N_{k_{nn}}\left(i\right)$ be defined with respect to the metric $d_{w}$. Then $R_{geom}\left(M\right)$ can be specified as a multicomponent functional that constrains geometric degradation via three typical mechanisms: scale degeneration, excessive anisotropy, and instability of local structure. One constructive variant is


$$\begin{array}{c} R_{geom}\left(M\right)=\rho_{\text{1}}R_{scale}\left(M\right)+\rho_{\text{2}}R_{anis}\left(M\right)+\rho_{\text{3}}R_{local}\left(M\right),\text{\textasciitilde{}}\rho_{j}\geq 0,\text{\textasciitilde{}}\sum_{j=1}^{\text{3}}\rho_{j}=1. \end{array}$$ (4)

Scale regularisation in (4) may be defined through stabilisation of the empirical distribution of pairwise distances, for example:


$$\begin{array}{c} R_{scale}\left(M\right)={\left(\frac{\text{1}}{\left|P\right|}\sum_{\left(i,j\right)\in P}D_{ij}\right)}^{-2}\left(\frac{\text{1}}{\left|P\right|}\sum_{\left(i,j\right)\in P}{\left(D_{ij}-\overset{―}{D}\right)}^{\text{2}}\right), \end{array}$$ (5)

where P={(i,j):1≤i<j≤N}; $\overset{―}{D}$ denote the mean value of $D_{ij}$ over P. Such an expression penalises situations in which all distances “collapse” to a small scale (which degrades separability), or in which the variance of distances becomes excessive relative to the mean scale (which is typically correlated with instability of the cluster structure). In practical implementations, the set P may be restricted to a subsample or defined on the basis of local neighbourhoods, which reduces computational complexity without loss of the regularisation effect. For N regime vectors of dimensionality p, evaluation of a single weighted pseudometric requires O(p) operations. An exhaustive evaluation over all regime pairs therefore has $O\left(N^{\text{2}}p\right)$ time complexity and, if the complete distance matrix is materialised, $O\left(N^{\text{2}}\right)$ memory complexity. This represents the principal worst-case computational bottleneck of the geometric regularisation rather than an intrinsic requirement to retain all pairwise distances. If $R_{\text{scale}}$ is estimated from a subset P containing m pairs, its distance-evaluation cost becomes O(mp) with O(m) storage. Likewise, the regularisation terms based on $k_{nn}$-neighbourhoods require only $O\left(Nk_{nn}\right)$ stored neighbourhood relations once the neighbourhood structure has been obtained. The cost of constructing that structure depends on whether exhaustive, indexed, or approximate neighbour search is employed. For the clustering stage, one spherical k-means fit requires O(NkpT) operations for k clusters and T iterations, whereas scoring N regimes against the resulting prototypes requires O(Nkp). These bounds distinguish the predominantly linear dependence of prototype-based processing on N, for fixed p and k, from the quadratic worst case associated with exhaustive pairwise geometry.

The anisotropic component in (4) can be introduced via the weighted covariance structure of the empirical sample. Let $\overset{―}{\mu }=\frac{\text{1}}{N}\sum_{i=1}^{N}\mu_{\delta_{i}}$, $\Sigma_{w}=\frac{\text{1}}{N}\sum_{i=1}^{N}\left(\mu_{\delta_{i}}-\overset{―}{\mu }\right){\left(\mu_{\delta_{i}}-\overset{―}{\mu }\right)}^{⊤}$, then


$$\begin{array}{c} R_{anis}\left(M\right)=\frac{\lambda_{max}\left(\Sigma_{w}\right)}{tr\left(\Sigma_{w}\right)+\varepsilon }, \end{array}$$ (6)

where $\lambda_{max}$ denotes the largest eigenvalue, tr the trace, and ε>0 a numerical stabilisation term. This constrains degeneracy in cases where the data are effectively distributed along a single direction and clustering becomes sensitive to noise. The weighting $\Sigma_{w}$ in this case is implemented indirectly through the components $\mu_{\delta_{i}}$, in which the weights have already been taken into account at the preceding stages of feature space construction.

Local regularisation in (4) can be specified through the consistency of distances to neighbours:


$$\begin{array}{c} R_{local}\left(M\right)=\frac{\text{1}}{N}\sum_{i=1}^{N}Var\left(\left\{D_{ij}:j\in N_{k_{nn}}\left(i\right)\right\}\right), \end{array}$$ (7)

which penalises situations in which local scales differ sharply from point to point, forming density “gaps” or “peaks” that lack semantic justification in the data.

The regularisation component $R_{inv}\left(M\right)$ in (4) is introduced to control the consistency of the resulting geometry with the defined execution invariants. Let the invariants be specified as a set of predicates $G={\left\{g_{m}\right\}}_{m=1}^{M}$, $g_{m}\left(S\right)=0$, for “normal” execution states, or in the form of soft constraints $g_{m}\left(S\right)\leq 0$. Since in this study the analysis is conducted in the feature space, it is appropriate to introduce an operator that transfers invariants into the observation space and subsequently into the feature space. For this purpose, an induced invariant violation function at the level of a transition $\delta_{r}$ can be defined as $vm\left(\delta_{r}\right)=\psi_{m}\left(\Delta_{r}\right)\geq 0$, where $\psi_{m}$ is a functional that measures the degree of violation of the m-th invariant based on the observed changes $\Delta_{r}$ (an example of such an invariant is the consistency between the type of a log event and the admissible range of changes in a resource metric within the corresponding time window).

Operationally, each invariant predicate $g_{m}$ induces a deterministic evaluation rule that maps every observed transition $\Delta_{r}$ to a scalar violation value through the corresponding functional $\psi_{m}$. For hard invariants, this rule distinguishes admissible and non-admissible transitions; for soft invariants, it quantifies the magnitude of deviation from the admissible relation. Thus, $v_{m}\left(\delta_{r}\right)=\psi_{m}\left(\Delta_{r}\right)$ is not an abstract penalty term but an explicitly evaluated scalar characteristic of the observed transition. Consequently, every invariant contributes to the regularisation term $R_{inv}\left(𝐌\right)$ through an operationally defined evaluation procedure, which ensures the reproducibility of the proposed invariant regularisation framework.

The invariant regulariser $R_{\text{inv}}\left(𝐌\right)$ couples transition-violation values with neighbourhood proximity in the mapped representation. Here 𝐌 denotes the empirical collection of regime vectors constructed by Φ. A fixed numerical stabiliser ε>0 keeps each denominator positive. A family with zero violation values for all transitions contributes zero. If every family has zero violations, the complete regulariser is zero. One constructive form is given in (8):


$$\begin{array}{c} R_{inv}\left(M\right)=\sum_{m=1}^{M}\eta_{m}\frac{\sum_{i=1}^{N}v_{m}\left(\delta_{i}\right)\cdot \sum_{j\in N_{k_{nn}}\left(i\right)}exp\left(-D_{ij}\right)}{\sum_{i=1}^{N}v_{m}\left(\delta_{i}\right)+\varepsilon }, \end{array}$$ (8)

where $\eta_{m}\geq 0$, $\sum_{m=1}^{M}\eta_{m}=1$, and $exp\left(-D_{ij}\right)$ plays the role of a proximity kernel. If, for transitions with a large invariant violation, their nearest neighbours remain “too close” (that is, $D_{ij}$ is small and $exp\left(-D_{ij}\right)$ is large), the penalty increases. The use of an exponential kernel corresponds to the standard heat-kernel interpretation of local proximity and allows the contribution of neighbours to be smoothly weighted as a function of distance. In this way, $R_{inv}$ forces the geometry of the feature space and the metric $d_{w}$ to “separate” into different regions those regimes that, according to invariants, have different natures. The parameters $\eta_{m}$ allow different importance levels of invariants to be reflected: critical security or correctness invariants may be assigned larger weights, whereas invariants related to performance or quality-of-service degradation may be weighted differently. Similarly, $\lambda_{\text{2}}$ controls the overall “rigidity” of invariant regularisation. For large values of $\lambda_{\text{2}}$, the feature space is more strongly constrained by invariants, which may increase interpretability but reduce sensitivity to non-formalised types of deviations.

Taken together, the specified parametric setting $\gamma_{c}$, $\lambda_{\text{1}}$, $\lambda_{\text{2}}$, as well as the constructive forms $R_{inv}\left(M\right)$ and $R_{geom}\left(M\right)$ (in the form (4)), transform the functional (3) into a computable criterion suitable for use as an objective function in a clustering task. This establishes a direct transition to the formalisation of clustering as a minimisation problem for I and to the introduction of weakly supervised mechanisms for calibrating the weights $w_{i}$, which are considered next.

Consider the clustering of the constructed vectors $\mu_{r}$ as the formation of a set of clusters $Q=\left\{q_{\text{1}},\text{\textbackslash{}ldots},q_{k}\right\}$, $q_{j}\subset \Delta$, $q_{i}⋂q_{j}=\emptyset \left(i\neq j\right)$, ${⋃}_{j=1}^{k}q_{j}=\Delta$, where $\Delta ={\left\{\delta_{r}\right\}}_{r=1}^{N}$ denotes the set of recorded execution transitions/intervals, and each $\delta_{r}$ corresponds to a point $\mu_{\delta_{r}}\in {\mathbb{R}}^{p}$ expressed via (2). In this formulation, a cluster is a formal group in the feature space rather than an “error class”. The semantics of clusters are established later through interpretation and weakly supervised steps.

Since in practical systems the distribution of features in (2) is mixed in nature (with some components reflecting frequency-based or aggregated properties of logs, others representing metric signals, and others invariant penalties), clustering requires a proximity measure that, on the one hand, is consistent with the geometry of $d_{w}$ and, on the other hand, does not reduce grouping solely to the scale of individual components. For this reason, angular similarity, which is invariant to uniform scalar multiplication, is an appropriate basic object for comparing two feature vectors. Accordingly, the pseudometric $d_{w}$ is used for constructing local structures and regularisation terms, whereas angular similarity $\rho_{w}$ is applied directly within the clustering procedure.

Let $\mu_{a},\mu_{b}\in {\mathbb{R}}^{p}$. Introduce the weighted angular similarity


$$\begin{array}{c} \rho_{w}\left(\mu_{a},\mu_{b}\right)={cos}_{w}\left(\mu_{a},\mu_{b}\right)=\frac{{\langle \mu_{a},\mu_{b}\rangle }_{w}}{{\|\mu_{a}\|}_{w}{\|\mu_{b}\|}_{w}}, \end{array}$$ (9)

where ${\langle \mu_{a},\mu_{b}\rangle }_{w}=\sum_{i=1}^{p}w_{i}\mu_{a}^{\left(i\right)}\mu_{b}^{\left(i\right)}$, ${\|\mu \|}_{w}=\sqrt{\sum_{i=1}^{p}w_{i}{\left(\mu^{\left(i\right)}\right)}^{\text{2}}}$, and $w_{i}\geq 0$ are component weights that have already appeared in the definition of $d_{w}$, but here they enter a normalised inner product. Such a choice ensures two properties that are critical specifically for the task of error detection, namely:

- the dominance of “scale-related” components is suppressed, for example overall logging intensity or large absolute values of resource metrics,

- the emphasis is shifted to the direction of the vector, that is, to relative feature proportions that are associated with invariants and the structure of execution.

From a technical perspective, the k-means algorithm is attractive as a basic unsupervised procedure due to its simplicity, scalability, and the availability of efficient implementations. At the same time, in its “classical” form, k-means minimises Euclidean inertia and is sensitive to scale. Therefore, in this work it is appropriate to employ it in an angular formulation, that is, as the minimisation of a divergence based on (9), which is equivalent to weighted spherical k-means.

For each cluster $q_{j}$, define its prototype (centre) $c_{j}\in {\mathbb{R}}^{p}$. Since the similarity is angular, it is natural to normalise the centres $c_{j}=c_{j}/{\|c_{j}\|}_{w}$. The clustering objective functional can then be formulated as the problem of maximising the total similarity to the prototypes (under the constraint ${\|C_{j}\|}_{w}=1$): $\underset{Q,{\left\{c_{j}\right\}}_{j=1}^{k}}{max}J\left(Q,c\right)=\sum_{j=1}^{k}\rho_{w}\left(\mu_{\delta_{r}},c_{j}\right)$. Equivalently, one can minimise the total angular loss


$$\begin{array}{c} \underset{Q,\left\{c_{j}\right\}}{min}L_{clust}\left(Q,c\right)=\sum_{j=1}^{k}\sum_{\delta_{r}\in q_{j}}\left(1-\rho_{w}\left(\mu_{\delta },c_{j}\right)\right) \end{array}$$ (10)

through the intra-cluster functional $L_{clust}$, induced by the chosen proximity measure ${\|C_{j}\|}_{w}=1$. This formulation corresponds precisely to the intuition of “grouping by feature structure rather than by absolute scale”.

The hierarchical functional I(𝐌) introduced in (3) integrates clustering with representation calibration. The overall optimisation scheme in (11) therefore has two levels.

1. Inner level (unsupervised): for fixed weights w and a fixed number of clusters k, Q is constructed by minimising $L_{clust}$.
2. Outer level (weakly supervised/model selection): the parameters k and w are selected according to criterion (3), where $I_{c}\left(M\right)$ includes clustering quality measures, $R_{geom}$ controls the geometry of the space, and $R_{inv}$ ensures invariant consistency. In general form, this can be written as the optimisation problem

$$\begin{array}{c} \underset{k\in \mathbb{N},w\in {\mathbb{R}}_{\geq 0}^{p}}{min}I\left(M;k,w\right), \end{array}$$ (11)

where $I\left(M;k,w\right)=\sum_{c\in C}\gamma_{c}I_{c}\left(M;Q_{k,w}\right)+\lambda_{\text{1}}R_{geom}\left(M;w\right)+\lambda_{\text{2}}R_{inv}\left(M;w\right)$, and $Q_{k,w}$ denotes the result of angular k-means based on (9) for given (k,w). It is important that clustering here is an embedded procedure: $Q_{k,w}$ is determined algorithmically, whereas I evaluates its quality and geometric/invariant validity.

Operationally, the framework forms a sequential analytical workflow. Raw telemetry is first partitioned into execution states and converted into directed structural changes $\Delta_{r}$. these changes are mapped by Φ into regime vectors $\mu_{\delta_{r}}$. The resulting regime space is equipped with the weighted geometry $d_{w}$, while $R_{\text{geom}}$ controls its structural regularity and $R_{\text{inv}}$ incorporates violations of domain-specific invariants. The joint criterion I(𝐌) then determines the calibrated geometry and cluster configuration, after which weighted angular similarity to the learned regime representatives provides the anomaly score. Thus, Eqs (1)–(11) correspond respectively to successive stages of telemetry transformation, regime representation, geometric calibration, invariant integration, model selection, and anomaly detection rather than to independent mathematical constructions.

## 3 Results and discussion

The experimental evaluation presented below assesses the detection performance of the invariant-guided execution-regime representation under limited supervision on operational KPI and log benchmarks. The evaluated targets are operator-labelled KPI anomalies and anomalous HDFS block sessions. The reported metrics quantify agreement with these operational labels. Attack detection and attribution to a security incident are outside the evaluated task.

The experimental design uses real operational observations from two public datasets without synthetic data generation. These datasets provide complementary metric-based and event-based settings for evaluating the framework. The 2018 AIOps Challenge KPI dataset [https://github.com/NetManAIOps/KPI-Anomaly-Detection] contains 29 individually labelled KPI time series with operator-marked anomalies and represents performance and reliability monitoring. The HDFS dataset from the LogHub collection [https://github.com/logpai/loghub/tree/master/HDFS] contains system logs grouped into Block-ID sessions with normal or anomalous labels associated with block execution outcomes. Neither dataset provides attack-specific ground truth for the present evaluation. The KPI and HDFS experiments separately instantiate the observation spaces $O_{M}$ and $O_{L}$ defined in Sect 2. The datasets do not provide synchronized log and metric observations of common execution entities. Their separate evaluation therefore tests the framework in each modality without evaluating a combined log–metric representation.

The selected datasets provide reproducible evaluations of the metric-based and log-based implementations of the mathematical formulation introduced in Sect 2. The AIOps benchmark supplies 29 labelled univariate KPI series. The HDFS benchmark comprises 11,175,629 log messages organized into 575,061 labelled block execution sessions. Both implementations use regime construction, weakly supervised feature weighting, weighted geometry, and invariant regularisation. Their descriptor sets, nominal references, calibrated weights, and fitted models are determined separately. Consequently, the results assess the applicability of these mathematical principles to continuous KPI observations and discrete log events. They do not establish transfer between modalities, alignment of the two fitted spaces, or an improvement from combining observations. The datasets also do not establish generalisation across industrial architectures, organizations, instrumentation stacks, or production environments. Such validation requires additional independently collected operational datasets.

The experiments use offline batch evaluation of recorded execution observations. Representation calibration and model fitting are completed before the held-out regimes are scored. No online adaptation or incremental parameter updates are performed. For each KPI series, the native sampling interval is identified from the most frequent positive difference between consecutive timestamps. The intervals are 60 or 300 seconds. Larger timestamp gaps are distinguished from the native interval when defining uninterrupted segments. Window length and stride are specified in observations, preserving a common descriptor construction across sampling rates. A 60-observation window therefore has a nominal duration of 60 or 300 minutes, and a 15-observation stride corresponds to 15 or 75 minutes. The overlapping windows have 75% overlap in both cases. The experiments consequently use comparable observation counts without assuming an identical physical duration for all KPI regimes.

The initial step consists in forming execution regimes on the basis of raw observations. For metric KPI data, regimes are defined by overlapping time windows of fixed length, selected so as to capture a representative phase of system behaviour while simultaneously fixing both stable states and gradual shifts. For log data, regimes correspond to individual execution sessions or sliding windows of event patterns, which reflects the discrete, event-driven nature of such observations. Regardless of the data type, subsequent analysis operates on homogeneous transitions $\delta_{r}$, formed via the structural difference operator introduced in Sect 2, which makes it possible to explicitly account for the directed evolution of execution.

The adopted offline protocol constitutes a deliberate validation stage of the proposed mathematical framework rather than an attempt to emulate a complete production monitoring pipeline. Its purpose is to determine whether the invariant-guided feature mappings, weighted pseudometric, invariant regularisation, and optimisation criterion introduced in Sect 2 are capable of constructing a stable and interpretable execution-regime space under fully reproducible experimental conditions. Production-scale monitoring introduces additional engineering mechanisms, including streaming ingestion, incremental feature maintenance, concept-drift adaptation, latency constraints, distributed state synchronisation, and bounded-memory processing. These mechanisms affect the deployment architecture rather than the mathematical formulation itself and therefore constitute a subsequent engineering stage beyond the scope of the present study. Accordingly, the adopted offline protocol isolates the contribution of the proposed mathematical apparatus and provides a reproducible basis for its experimental validation before future extension to streaming production environments.

At the next stage, each transition $\delta_{r}$ is mapped onto a feature vector $\mu_{\delta_{r}}$ in accordance with formula (2). The local functions are instantiated for the observation modality available in each dataset. Nonlinear transformations $\sigma_{i}$ normalize the descriptors before construction of the corresponding regime representation. HDFS uses the log-derived descriptor functions, whereas AIOps uses the metric-derived functions. The common formulation specifies how observations are transformed and calibrated. It does not require identical feature coordinates or a shared fitted space across datasets. The following operational definitions describe the two separately evaluated implementations.

For HDFS, session membership in development and test sets is fixed before constructing session differences. Within each partition, Block-ID sessions are ordered by the timestamp of their first raw log event. Ties are resolved by the position of that event in the original log file and then by the numerical Block ID. This ordering uses observation metadata and does not use anomaly labels.

Let $z_{r}$ denote the session representation before directed differencing. For consecutive sessions within a partition, the directed difference is $z_{k}-z_{r-1}$. Since these sessions concern independent blocks, this difference compares successive observed execution patterns. It does not describe a causal transition of the same block. No pair of individual sessions is formed across a training, validation, or test boundary.

The first session of each partition is compared with a fixed nominal reference $z_{\text{ref}}$. This reference is the componentwise mean of the pre-difference representations of disclosed normal development sessions. During cross-validation, it is estimated exclusively from disclosed normal sessions in the corresponding training fold. For final test evaluation, it is estimated from the development set and then held fixed. Thus, the first difference is $z_{\text{1}}-z_{\text{ref}}$. Every session retains one evaluated representation, and the first session is not forced to have zero difference.

Each transition inherits the label of its destination session, $y\left(\delta_{r}\right)=y\left(b_{r}\right)$, where $b_{r}$ is the session being evaluated. A normal-to-anomalous pair is labelled anomalous, whereas an anomalous-to-normal pair is labelled normal. The target is therefore the operational outcome of the destination execution rather than the presence of a change between two executions. A large difference can describe either entry into abnormal behaviour or return to normal behaviour and is not, by itself, an anomalous ground-truth label.

For AIOps, chronological partitions are defined before constructing windows. Each window belongs to one KPI series and lies entirely within its assigned partition. Let $W_{r}$ denote a 60-sample window and let $y_{t}\in \left\{0,1\right\}$ be the supplied point label. The window label is $y\left(W_{r}\right)={max}_{t\in W_{r}}y_{t}$. A window is therefore anomalous if it contains at least one labelled anomalous point. Windows are advanced by 15 samples. Directed differences are formed between consecutive admissible windows of the same series within the same partition, and the transition inherits the destination-window label, $y\left(\delta_{r}\right)=y\left(W_{r}\right)$. No transitions are constructed between different KPI series or across partition boundaries.

The first admissible window within each series and partition is compared with a nominal window-descriptor reference estimated from disclosed normal development windows. The reference is calculated separately for each series when such windows are available. Otherwise, the componentwise mean of disclosed normal development window descriptors is used in the development-standardized metric descriptor space. During cross-validation, reference estimation uses only the training fold. The resulting reference is fixed before transforming the corresponding validation or test windows. Overlapping windows remain distinct evaluation records. A labelled anomalous point can consequently contribute to several anomalous windows. The reported metrics evaluate these window-level decisions without point adjustment or replacement of individual predictions over an anomalous interval.

For reproducibility, the abstract constructs introduced in Eqs (1)–(11) were instantiated through dataset-specific operational rules. For the HDFS benchmark, the LogHub representation was used: raw logs are grouped into block-ID execution traces, each trace has a normal/anomalous ground-truth label, and the preprocessed files include HDFS_templates.csv, Event_traces.csv, Event_occurrence_matrix.csv, HDFS.npz, and anomaly_label.csv. Thus, an elementary log observation $x\in \Delta_{r}^{L}$ corresponded to a parsed event-template occurrence within a block trace, and $\Delta_{r}^{L}$ was defined as the directed structural difference between consecutive block-trace representations. The local log functions $l_{i}$ were instantiated through frequency-related, sequential, and semantic/template-identity descriptor classes extracted from parsed event templates. Operational representatives of these classes included event-template occurrence statistics, normalised template frequencies, and sequential variation of template occurrence between adjacent trace representations. For each HDFS block trace, let $n_{re}$ denote the number of occurrences of template e in trace r, and let $N_{r}=\sum_{e}n_{re}$. The frequency representative was defined as the normalised occurrence $f_{re}=n_{re}/N_{r}$. Directed sequential variation between two consecutive trace representations was calculated as $\Delta f_{re}=f_{re}-f_{r-1,e}$, thereby preserving the sign of template appearance, disappearance, or frequency change. Template identity was preserved by maintaining a fixed coordinate for each parsed event template in the feature representation. Missing occurrence of an already defined template in a trace was represented by zero rather than by removing the corresponding coordinate. This produced a deterministic fixed-dimensional log-side representation before weighting and nonlinear transformation. The log-side aggregation weights $\alpha_{k}$ were initialised uniformly within descriptor classes and refined under weak supervision while preserving non-negativity and normalisation. Since no synchronised KPI stream is provided for the same HDFS block traces, the metric component of (1) was not imputed for this benchmark; the HDFS configuration therefore instantiated only $O_{L}$, $\Delta_{r}^{L}$, $l_{i}$, and $\alpha_{k}$.

For the 2018 AIOps Challenge KPI benchmark, the data were treated as 29 labelled univariate KPI time series collected from real Internet-service monitoring environments. An elementary metric observation $y\in \Delta_{r}^{M}$ corresponded to one timestamped KPI value, and each execution regime was represented by a fixed temporal window. The baseline configuration used a 60-sample window and a 15-sample stride. For KPI series sampled at one-minute resolution, this corresponds to one-hour windows with 75% overlap. The metric transition $\Delta_{r}^{M}$ was defined as the directed difference between two consecutive window representations. The local metric functions $m_{j}$ were instantiated through amplitude-based, temporal, and local-deviation descriptor classes. Operational representatives of these classes included normalised level, deviation from the preceding window, local temporal slope, and within-window variability. For a KPI window $W_{r}=\{y_{t}{\}}_{t=1}^{L}$, these representatives were evaluated deterministically after robust series-level standardisation. The level descriptor was defined as the median value within $W_{r}$. The inter-window deviation as the difference between the median values of $W_{r}$ and $W_{r-1}$. The local temporal slope as the least-squares coefficient obtained by regressing the standardised KPI values on their within-window time indices; and within-window variability as the interquartile range of the standardised values in $W_{r}$. These four quantities were calculated for every admissible window before construction of the corresponding directed regime transition. The metric-side aggregation weights $\beta_{k}$ were initialised uniformly across descriptor classes and refined under the same weak-supervision constraint. Since aligned log events are not provided for the KPI benchmark, the log component of (1) was not synthetically generated; this configuration instantiated only $O_{M}$, $\Delta_{r}^{M}$, $m_{j}$, and $\beta_{k}$.

The nonlinear mappings $\sigma_{i}$ in (2) were implemented as robust standardisation for all descriptors, with logarithmic compression used only for non-negative heavy-tailed frequency or magnitude descriptors. The feature-construction pipeline was applied in a fixed order. First, raw observations were converted into dataset-specific regime representations. Second, directed differences between consecutive regime representations were formed. Third, the deterministic descriptor operators defined above were evaluated. Fourth, non-negative heavy-tailed frequency or magnitude descriptors were subjected to logarithmic compression, where applicable. Fifth, robust standardisation was applied and the transformed coordinates were assembled into the regime vector $\mu_{\delta_{r}}$. No unavailable telemetry modality was synthetically imputed: the HDFS experiment instantiated the log branch of the framework, whereas the AIOps experiment instantiated the metric branch. The dimensionality p of $\mu_{\delta_{r}}$ was therefore determined by the instantiated descriptor set: for HDFS, by the number of parsed event-template descriptors; for KPI data, by the four window-level metric descriptor representatives per KPI series. The weighted pseudometric $d_{w}$ used q=2. Feature weights $w_{i}$ were initialised uniformly, $w_{i}=1/p$, and updated under the constraints $w_{i}\geq 0$ and $\sum_{i}w_{i}=1$. Weight calibration was implemented by deterministic projected coordinate search on the probability simplex. Starting from the uniform vector $w^{\left(0\right)}=(1/p,\ldots ,1/p)$, each coordinate was considered successively in the fixed feature order. For coordinate i, candidate positive and negative perturbations of magnitude h were generated whenever admissible, after which the remaining coordinates were rescaled proportionally so that non-negativity and $\sum_{i}w_{i}=1$ were preserved. For each candidate weight vector, the inner spherical k-means problem in (10) was refitted and the complete criterion I(𝐌;k,w) in (11) was recomputed. A candidate update was accepted only if it reduced I. The initial step was h=0.10. After a complete coordinate pass without an accepted update, the step was halved. Calibration terminated when $h<10^{-3}$ or after 100 complete coordinate passes, whichever occurred first. This procedure was applied identically to all datasets and supervision settings. The geometric regularisation in (4) used the three components defined in Sect 2 (scale, anisotropy, and local-neighbourhood stability) with initial values $\rho_{\text{1}}=\rho_{\text{2}}=\rho_{\text{3}}=1/3$. The nearest-neighbour parameter in (7) and (8) was fixed at $k_{nn}=10$.

The invariant families were implemented as soft consistency constraints on observed regime changes. They encode reference relationships that are expected to remain approximately stable under nominal execution. For each fitting partition, let 𝒩 contain the changes associated with its disclosed nominal regimes. Reference membership is determined by the disclosed label of the evaluated regime. It does not require both constituent regimes of a change to have disclosed nominal labels. The reference therefore describes changes associated with nominal-labelled regimes rather than exclusively transitions between two confirmed nominal regimes. Concealed labels are not consulted.

Within development cross-fitting, reference estimation excludes the held-out partition. For the final fit, references are recomputed from disclosed nominal regimes in the complete development partition. Test observations and test labels are excluded from both procedures. Reference statistics remain fixed during feature-weight calibration, so changes in w cannot redefine the nominal constraints being enforced.

Each family produces a non-negative departure statistic $h_{m}\left(\Delta_{r}\right)$. Let $G_{m}$ be its nominal-reference distribution. Define $\tau_{m}=Q_{0.95}\left(G_{m}\right)$, $b_{m}=max\;\left(Q_{0.75}\left(G_{m}\right)-Q_{0.25}\left(G_{m}\right),\text{\textbackslash{}hspace\{0.5em}\}0.10\text{\textbackslash{}hspace\{0.17em}\;\}\tau_{m},\varepsilon \right)$, $\varepsilon =10^{-6}$.

The induced violation functional is $v_{m}\left(\delta_{r}\right)=\psi_{m}\left(\Delta_{r}\right)=\frac{[h_{m}(\Delta_{r})-\tau_{m}{]}_{+}}{b_{m}+[h_{m}\Delta_{r})-\tau_{m}{]}_{+}}$, $\text{\textbackslash{}hspace\{0.5em}\}[x{]}_{+}=max(x,0)$. Here $Q_{p}\left(G\right)=inf\{x|G(xgep\}$. The functional is zero inside the nominal tolerance, increases monotonically with its exceedance, and remains below one. The reference-spread term limits sensitivity to very small nominal tolerances. The numerical floor makes the definition valid for a constant reference distribution. The 95th percentile defines a fixed empirical tolerance for an invariant family. It is independent of the subsequently calibrated anomaly-score decision threshold.

For HDFS, let J denote the number of event templates, $n_{rj}$ their session occurrence counts, and $b_{rj}=1(n_{rj}>0)$, $\text{\textbackslash{}hspace\{0.5em}\}f_{rj}=n_{rj}/max\left(1,\sum_{\ell =1}^{J}n_{r\ell }\right)$. The observed changes are $d_{rj}^{\left(b\right)}=b_{rj}-b_{r-1,j}$, $d_{rj}^{\left(f\right)}=f_{rj}-f_{r-1,j}$.

Template-occurrence consistency is evaluated through the nominal rarity of appearance, disappearance, or persistence. Its departure statistic is $h_{\text{1}}^{\text{HDFS}}\left(\Delta_{r}\right)=-\frac{\text{1}}{J}\sum_{j=1}^{J}log{\hat{p}}_{j}\left(d_{rj}^{\left(b\right)}\right)$, ${\hat{p}}_{j}\left(a\right)=\frac{\sum_{i\in 𝒩}1(d_{ij}^{\left(b\right)}=a)+\text{\textbackslash{}hspace\{0.17em}\;}{1}/2\}\left|𝒩\right|+3/2$, a∈{−1,0,1}. Smoothing makes the departure statistic finite when a change category is absent from the reference sample. A template whose occurrence status is normally stable contributes little when that status persists.

Template-frequency stability is evaluated through marginal deviations of relative-frequency changes. For each template, define $c_{j}={median}_{i\in 𝒩}d_{ij}^{\left(f\right)}$, $s_{j}=max\;\left(1.4826\text{\textbackslash{}hspace\{0.17em}\;\}{median}_{i\in 𝒩}|d_{ij}^{\left(f\right)}-c_{j}|,\text{\textbackslash{}hspace\{0.17em}\;\}0.01\text{\textbackslash{}hspace\{0.17em}\;\}Q_{0.95}\left\{\right|d_{ij}^{\left(f\right)}|{\}}_{i\in 𝒩},\varepsilon \right)$, $\text{\textbackslash{}hspace\{0.5em}\}z_{rj}=\frac{d_{rj}^{\left(f\right)}-c_{j}}{s_{j}}$. Its departure statistic is $h_{\text{2}}^{\text{HDFS}}\left(\Delta_{r}\right)=\frac{\text{1}}{J}\sum_{j=1}^{J}|z_{rj}|$. The scale floor depends on the reference magnitude of the corresponding descriptor. It therefore avoids imposing the same substantive absolute tolerance on templates with different frequency scales.

Trace-composition consistency is evaluated through joint relationships among frequency changes. Let $z_{r}=(z_{r1},\ldots ,z_{rJ}{)}^{⊤}$. The reference covariance matrix C is the sample covariance of the nominal-reference vectors after coordinate-wise clipping to [−5,5]. Clipping is used only for covariance estimation. Define $C_{\text{reg}}=\text{\textbackslash{}hspace\{0.5em}\}0.90\text{\textbackslash{}hspace\{0.17em}\;\}C+0.10\text{\textbackslash{}hspace\{0.17em}\;\}diag\left(C\right)+\varepsilon max\;\left(\frac{tr\left(C\right)}{J},1\right)I_{J}$. The departure statistic is $h_{\text{3}}^{\text{HDFS}}\left(\Delta_{r}\right)=\sqrt{\frac{z_{r}^{⊤}C_{\text{reg}}^{-1}z_{r}}{J}}$.

Evaluation uses the unclipped vector $z_{r}$. The positive diagonal term makes the inverse defined despite dependencies among relative frequencies or constant coordinates. The second family measures marginal departures, whereas the third measures departures from their joint nominal organisation. Each HDFS family uses the empirical distribution of its departure statistic over 𝒩 as $G_{m}$.

For KPI series a, let $L_{ar}$, $B_{ar}$, and $V_{ar}$ denote the window median, least-squares temporal slope, and interquartile range defined above. The three change descriptors are $u_{ar1}=L_{ar}-L_{a,r-1}$, $\text{\textbackslash{}hspace\{0.5em}\}u_{ar2}=B_{ar}-B_{a,r-1}$, $u_{ar3}=V_{ar}-V_{a,r-1}$.

Reference estimation combines series-specific and shared nominal information after development-only series standardisation. Let $n_{a}=\left|{𝒩}_{a}\right|$ and let $F_{am}$ be the empirical distribution of descriptor m over the disclosed nominal fitting regimes of series a. The pooled distribution $F_{\text{pool},m}$ is the equally weighted mixture of these empirical distributions over series with at least one disclosed nominal fitting regime. Define $\omega_{a}=\frac{n_{a}}{n_{a}+\text{\textbackslash{}hspace\{0.5em}}20\}$, $\text{\textbackslash{}hspace\{0.5em}\}F_{am}^{*}=\omega_{a}F_{am}+(1-\omega_{a})F_{\text{pool},m}$.

When $n_{a}=0$, the reference is $F_{\text{pool},m}$. This continuous weighting retains series-specific information while reducing dependence on very small local reference samples. The constant 20 is fixed across annotation budgets and is not selected using test performance.

For each series and descriptor, define $c_{am}=Q_{0.50}\left(F_{am}^{*}\right)$, $s_{am}=max\;\left(1.4826\text{\textbackslash{}hspace\{0.17em}\;\}Q_{0.50}\left(\right|U-c_{am}\left|\right),0\mathrm{.01}\text{\textbackslash{}hspace\{0.17em}\;\}Q_{0.95}\left(\right|U\left|\right),\varepsilon \right)$, $\text{\textbackslash{}hspace\{0.5em}\}U\;F_{am}^{*}$. The departure statistics are $h_{am}^{\text{KPI}}\left(\Delta_{ar}\right)=\frac{\left|u_{arm}-c_{am}\right|}{s_{am}}$, m=1,2,3.

The nominal-reference distribution $G_{am}$ is the distribution of $|U-c_{am}|/s_{am}$ under $F_{am}^{*}$. It supplies the tolerance and spread used in $\psi_{m}$. The three functionals respectively constrain departures in changes of level, slope, and variability. All local and pooled reference quantities use only the permitted fitting observations.

The invariant-family weights remain fixed at $\eta_{\text{1}}=\eta_{\text{2}}=\eta_{\text{3}}=1/3$. Weak supervision contributes both to the label-consistency component and to identifying the nominal reference regimes. The invariant-family weights are not additional free hyperparameters. The compactness, separability, and label-consistency components retain equal weights $\gamma_{\text{1}}=\gamma_{\text{2}}=\gamma_{\text{3}}=1/3$. The candidate grids remain k∈{2,…,12} and $\lambda_{\text{1}},\lambda_{\text{2}}\in \{0,0.05,0.10,0.20,0\mathrm{.50}\}$. For a fixed candidate configuration, feature-weight calibration minimises the integrated criterion in (11). Configuration selection uses held-out development performance as specified below.

The operational configuration used in all experiments was therefore fixed as follows: q=2, $k_{nn}=10$, $\rho_{\text{1}}=\rho_{\text{2}}=\rho_{\text{3}}=1/3$, $\eta_{\text{1}}=\eta_{\text{2}}=\eta_{\text{3}}=1/3$, $\gamma_{\text{1}}=\gamma_{\text{2}}=\gamma_{\text{3}}=1/3$, k∈{2,…,12}, $\lambda_{\text{1}},\lambda_{\text{2}}\in \left\{0,0.05,0.10,0.20,0\mathrm{.50}\right\}$, and weak-supervision levels of 5% and 1%. Implementation-level reproducibility was additionally assessed by repeating the complete calibration and model-selection procedure over 20 independent spherical k-means initialisations for each dataset and weak-supervision level. All feature-construction rules, initial feature weights, hyperparameter grids, stopping criteria, and weak-label subsets were held fixed across repetitions, so that only the stochastic initialisation of spherical k-means varied. The same discrete model configuration $\left(k,\lambda_{\text{1}},\lambda_{\text{2}}\right)$ was selected in 85% of repetitions for AIOps at 1% supervision, 90% for AIOps at 5%, 80% for HDFS at 1%, and 85% for HDFS at 5%. The corresponding standard deviations of F1 across the 20 repetitions were 0.010, 0.008, 0.015, and 0.011, respectively. Stability of the calibrated feature weights was assessed by the mean absolute deviation from the repetition-wise mean weight vector; this value was 0.024 for AIOps at 1% supervision, 0.018 for AIOps at 5%, 0.033 for HDFS at 1%, and 0.026 for HDFS at 5%. These results show that both the selected hyperparameter configuration and the resulting detection performance remain stable with respect to clustering initialisation. Consequently, the experimental instantiation specifies the data representation, deterministic feature-construction rules, fixed parameters and admissible search ranges, weight-calibration algorithm, optimisation stopping criteria, and model-selection procedure associated with Eqs (1)–(11), thereby providing an operationally reproducible specification of the experimental pipeline.

The resulting regime vectors are interpreted in geometric terms. For this purpose, a weighted angular similarity measure $\rho_{w}$, defined in (9), is employed. It induces the structure of the space and enables the comparison of regimes while accounting for the differing significance of individual features. Clustering is carried out using spherical k-means by minimising the within-cluster objective in (10). In this setting, it is used primarily to organise the regime space, because the resulting partition is then used to assess deviations and regime stability.

Degenerate angular inputs are identified after descriptor transformation and weighting. Define ${\|\mu \|}_{w}={\left(\sum_{i}w_{i}\mu_{i}^{\text{2}}\right)}^{1/2}$, $\varepsilon_{\text{ang}}=10^{-12}$. A regime is treated as directionally degenerate when ${\|\mu_{\delta_{r}}\|}_{w}\leq \varepsilon_{\text{ang}}$. This includes a nonzero feature vector whose nonzero coordinates receive zero effective weight. A zero raw difference is not necessarily degenerate after development-fitted centring and descriptor transformation.

For spherical k-means calculations, the computational similarity of a degenerate regime to every nondegenerate prototype is set to zero. Such regimes contribute a constant angular loss and no direction to prototype updates. Assignment ties are resolved by the smallest cluster identifier. This assignment is computational and does not establish operational normality. Degenerate regimes remain available for weak-label accounting, invariant evaluation, and final scoring.

If an empty cluster or cancellation produces a degenerate prototype, it is reinitialized from a nondegenerate development regime with the largest current angular loss. Ties are resolved by record identifier. No validation or test observation is used for reinitialization. The number of directional clusters is restricted to the number of available nondegenerate fitting regimes.

For final scoring, absence of a direction is not treated as either geometric agreement or evidence of abnormality. Instead, the score uses the three bounded invariant violations defined above, $s_{\text{inv}}\left(\delta_{r}\right)=2\left(\frac{\text{1}}{\text{3}}\sum_{m=1}^{\text{3}}v_{m}\left(\delta_{r}\right)\right)$. The factor 2 places this fallback in the same [0,2] range as $1-\rho_{w}$. It does not make the two components probabilistically equivalent. The complete scoring rule, including this branch, is included in development threshold calibration.

The experimental implementation distinguishes representation calibration from selection of the discrete configuration. Eqs (10)–(11) define the clustering and integrated representation criteria. In the reported implementation, w is calibrated by minimising I for each fixed triplet $\left(k,\lambda_{\text{1}},\lambda_{\text{2}}\right)$, while the triplet is selected using held-out development performance. Candidate cluster numbers are k∈{2,…,12}, and each regularisation coefficient belongs to {0,0.05,0.10,0.20,0.50}. Configurations with $\lambda_{\text{2}}=0$ remain eligible throughout selection.

For every candidate triplet and development cross-fitting fold, preprocessing, nominal-reference estimation, and representation calibration use only the fitting partition. The label-consistency component uses only its disclosed labels. Candidate weight updates are evaluated by refitting spherical k-means and recomputing I. The resulting model then scores the held-out partition, whose observations and labels have not entered fitting.

Candidate configurations are compared using the pooled disclosed out-of-fold predictions from the five-fold development protocol defined below. For each candidate, scores are transformed using the corresponding fitting-partition nominal score distribution. Its threshold is selected by the stated development F1 rule. The candidate with the highest resulting development F1 is retained. Ties within $10^{-6}$ are resolved by the smaller k, followed by the smaller $\lambda_{\text{1}}+\lambda_{\text{2}}$, and then the smaller $\lambda_{\text{2}}$.

Penalised training-objective values are not compared across different regularisation coefficients. This avoids favouring zero coefficients simply because they remove non-negative penalties. The selected configuration is refitted on the complete development partition, with its nominal references recomputed from the disclosed nominal development regimes. Its decision threshold is fixed from development out-of-fold predictions before test scoring. The fully labelled comparison in Table 4 uses its existing held-out validation partition for configuration and threshold selection.

The following configurations identify the reference fits with initialisation seed 42 underlying the fixed-geometry comparison. On AIOps, the selected triplet $\left(k,\lambda_{\text{1}},\lambda_{\text{2}}\right)$ is (3,0.05,0.10) at 1% supervision and (4,0.05,0.10) at 5% supervision. On HDFS, it is (4,0.10,0.20) at 1% supervision and (6,0.05,0.20) at 5% supervision. These are also the modal configurations across the 20 initialisation repetitions. They are selected in 17, 18, 16, and 17 repetitions, respectively, corresponding to the previously reported frequencies of 85%, 90%, 80%, and 85%. The fully labelled HDFS comparison in Table 4 selects (6,0.05,0.10).

Every reported reference configuration has $\lambda_{\text{2}}>0$. In each reference fit, at least one invariant family produces positive violation values among the fitting regimes, giving $R_{\text{inv}}>0$. The invariant term therefore contributes to representation calibration rather than being present only in the formal objective. Its predictive contribution is evaluated separately by removing the term in the ablation experiment.

The selected k describes subdivisions of execution-regime geometry rather than the number of ground-truth classes. Multiple clusters can represent distinct nominal configurations or different forms of departure within a binary detection task. The larger HDFS configuration at 5% supervision is consistent with resolving additional event-composition patterns when more labelled regimes are available. This interpretation does not assign a failure type to each cluster. Likewise, the different numerical values of $\lambda_{\text{2}}$ do not establish a cross-dataset ranking of invariant importance.

Configuration selection is repeated within development data for every initialisation and weak-label subset. The reported modal frequencies concern the original fixed-subset initialisation experiment. They do not describe the additional weak-label-subset experiment. Individual selected configurations are retained with the per-run results.

The weak-supervision experiments separate development regimes from an independent test set before representation fitting, weight calibration, model selection, or decision calibration. Development labels are disclosed only within the specified annotation budget, while test labels are used exclusively for evaluation. Threshold calibration reuses the disclosed development labels through out-of-fold predictions, without introducing an additional fully labelled validation set. This protocol evaluates whether the framework can construct an informative execution-regime representation and calibrate its decision rule under limited supervision. For HDFS, the test partition is identical to that used in the controlled experiment described later in this section. It contains 115,013 Block-ID sessions, comprising 111,645 nominal and 3368 anomalous sessions, with an anomaly prevalence of 2.928%. The remaining 460,048 sessions form the development partition, comprising 446,578 nominal and 13,470 anomalous sessions. Block IDs are disjoint between development and test partitions, and regime representations are constructed independently within each partition. For AIOps, each of the 29 KPI series is divided chronologically into an initial 80% development segment and a final 20% test segment before window construction. Only windows and consecutive-window transitions contained entirely within one segment are retained. The resulting development and test sets contain 80,000 and 20,000 regimes, respectively. The development set contains 4268 anomalous and 75,732 nominal regimes; the test set contains 1067 anomalous and 18,933 nominal regimes, giving a test anomaly prevalence of 5.335%. A window is labelled anomalous when it contains at least one timestamp annotated as anomalous, and a transition inherits the label of its destination window. Evaluation is performed at the regime level without point adjustment. Within each dataset, the test set is unchanged between supervision settings.

Two annotation budgets are considered, with r=0.01 and r=0.05 denoting the proportions of development anomalies whose labels are disclosed. The same proportion of nominal development labels is disclosed, using $\lfloor {rN}_{c}\rfloor$ labelled regimes from each class c. The resulting HDFS budgets contain 134 anomalous and 4465 nominal labels at 1%, and 673 anomalous and 22,328 nominal labels at 5%. The AIOps budgets contain 42 anomalous and 757 nominal labels at 1%, and 213 anomalous and 3786 nominal labels at 5%. Class-specific sampling uses a fixed random ordering, with the 1% subset nested within the 5% subset. All other development labels remain concealed. The same disclosed subsets and test regimes are used for every compared method. Thus, 1% and 5% specify annotation availability, not the proportion of test regimes flagged as anomalous.

Disclosed development regimes are excluded from test metrics by partition membership. They contribute labels to development model selection and receive out-of-fold scores for decision calibration. Within each calibration fold, held-out regimes and their labels are excluded from fitting and model selection. Final evaluation uses the fixed test-regime manifest defined above. Any exclusions required by window or transition construction are applied before that manifest is fixed. All compared methods, initialisation repetitions, and bootstrap samples use the same test identifiers and ground-truth labels.

Data-dependent standardisation parameters, calibrated feature weights, nominal-reference statistics, and model-selection quantities are estimated from the permitted development observations. The label-consistency component is evaluated only on disclosed development labels. Concealed development labels and test labels do not enter this component. Test labels are accessed only after the final predictions have been fixed. The fixed-geometry bootstrap resamples completed test prediction records and does not resample disclosed development regimes.

The original 20 repetitions assess sensitivity to spherical k-means initialisation. Development and test manifests, weak-label identities, cross-fitting folds, feature-construction rules, hyperparameter grids, and stopping criteria remain fixed. Only the random seed controlling spherical k-means initialisation changes. Each repetition reruns representation calibration, model selection, cross-fitted threshold calibration, and final scoring. Changes in fitted weights, selected configurations, and thresholds are therefore downstream effects of initialisation rather than additional independently varied experimental factors.

After fixing the selected model configuration (k,w), each transition receives the score $s\left(\delta_{r}\right)=\left\{ \begin{array}{cc} @ll@1-{max}_{1\leq j\leq k}\rho_{w}\left(\mu_{\delta_{r}},c_{j}\right), & {\|\mu_{\delta_{r}}\|}_{w}>\varepsilon_{\text{ang}},\\ \frac{\text{2}}{\text{3}}\sum_{m=1}^{\text{3}}v_{m}\left(\delta_{r}\right), & {\|\mu_{\delta_{r}}\|}_{w}\leq \varepsilon_{\text{ang}}. \end{array}\right.$.

The second branch specifies scoring when the angular direction is undefined. All regimes remain in the evaluation set, and both branches use the same development-calibrated decision threshold. For nondegenerate regimes, increasing scores indicate weaker directional agreement with the closest fitted prototype. The prototypes represent groups of development observations. Compactness alone does not establish that a group is nominal.

Invariant regularisation and weak-label consistency influence representation and model selection. They do not convert the prototype-proximity score into a cluster-level probability of abnormality. If an anomalous pattern forms its own compact development cluster, its members can have high similarity to that cluster’s prototype and consequently receive low scores. In the limiting case of perfect directional agreement, the angular score is zero irrespective of the cluster’s operational label.

Geometric separation and anomaly scoring therefore have distinct roles. Separating an anomalous group can improve the organization of the representation without increasing the scores of its members when that group contributes a prototype. The reported results establish detection performance in the evaluated settings rather than guaranteed detection of every compact anomalous cluster. Restricting scoring to nominal prototypes or adding calibrated cluster risk would extend the detector and requires separate evaluation.

Decision calibration uses five-fold cross-fitting within the development partition. HDFS folds are formed from disjoint Block-ID sessions, while AIOps folds comprise contiguous temporal blocks within each KPI series. Windows or transitions sharing raw observations with a held-out block are excluded from its corresponding fitting sample. In each fold, preprocessing statistics, feature weights, model configuration, and prototypes are estimated without the held-out regimes or their disclosed labels. The fitted model then produces out-of-fold scores for the disclosed regimes in that fold.

To make scores comparable across fold-specific geometries, each score is mapped to its empirical percentile relative to the disclosed nominal regimes used for fitting that fold. This monotone transformation preserves score ordering. The decision threshold maximises F1 over the pooled out-of-fold predictions of the disclosed development regimes; ties are resolved in favour of the higher threshold. The final model is fitted on the complete development partition using the same annotation budget, and its scores are transformed using the corresponding development nominal-reference distribution. The selected percentile threshold is then fixed for test evaluation. Neither concealed development labels nor test labels enter threshold calibration. The observed test alert fraction is therefore independent of the nominal 1% or 5% annotation budget.

For the quantitative comparison, DeepLog, PLELog, and ADS represent sequential log prediction, probabilistic label estimation for log sequences, and semi-supervised KPI detection. Their selection reflects the observation types and limited-label setting considered in this study. Their original experimental protocols differ from the common protocol used here. The retained components and adaptations are specified below. Invariant Mining is additionally discussed as a direct methodological antecedent of invariant-based HDFS problem detection.

DeepLog [21] learns conditional next-log-key distributions from normal execution using an LSTM. Its log-key detector identifies observations outside the accepted next-key candidates. The original framework also includes parameter-value detection, workflow diagnosis, and optional online adaptation. The present HDFS comparison evaluates the log-key prediction component. It supplies a sequential alternative to the proposed representation of regime changes through a calibrated pseudometric.

PLELog [22] combines semantic log representations, HDBSCAN-based probabilistic label estimation, and an attention-based GRU classifier. Known normal sequences anchor the estimation of labels for the remaining training sequences. Its classifier subsequently produces sequence-level anomaly estimates. This supplies a comparison in which unlabelled development sequences contribute to detector training through estimated class probabilities.

ADS [36] addresses deployment of anomaly detectors across individual KPI streams. It combines shape-based stream grouping, detector-severity features, and contrastive pessimistic likelihood estimation with a random-forest base classifier. Its original setting transfers information from labelled representative streams to unlabelled target streams. The present implementation retains this organisation while adapting the supervised records and classifier outputs to the window-level AIOps evaluation.

Overall, the selected analogues cover three distinct paradigms, namely local sequential modelling of events, probabilistic semi-supervised interpretation of log data, and KPI-oriented detection. This comparison provides a principled background for contrasting them with the geometric model of execution regimes, as it allows differences to be analysed not only in terms of quantitative quality metrics but also with respect to the organisation of the observation space and the level of behavioural generalisation of the system.

Invariant Mining by Lou et al. [40] is a direct predecessor of invariant-based HDFS problem detection. It groups related log messages and mines sparse integer relations between their occurrence counts. Such relations take the form $a_{\text{0}}+\sum_{j=1}^{J}a_{j}n_{j}=0$, where $n_{j}$ counts occurrences of message type j within a group. Violations identify departures from mined execution-flow relations. The present framework instead specifies tolerance-based functionals of regime changes and estimates their nominal references from disclosed normal development regimes. These constraints describe statistical regularity rather than automatically recovered program identities. They enter feature-weight calibration and thereby influence distances and neighbourhoods in the regime space. The contribution concerns this integration with regime geometry and metric adaptation. Invariant-based detection itself is established prior work.

The weak-supervision comparison evaluates the invariant-guided framework against three relevant detection paradigms under common data partitions, disclosed-label budgets, and decision calibration. Architectural and representation differences remain part of this method-level comparison. Table 2 reports paired F1 differences, and Table 3 provides complementary detection and ranking metrics. Table 4 separately considers recent neural detectors under more extensive supervision. Table 5 examines the contributions of invariant regularisation, geometric regularisation, and learned weights within the proposed framework.

**Table 2:** Paired-bootstrap intervals for test F1 differences.

Metric	AIOps KPI (1%)	AIOps KPI (5%)	HDFS (1%)	HDFS (5%)
Compared baseline	ADS	ADS	PLELog	DeepLog
Baseline test F1	0.646	0.742	0.651	0.724
Proposed test F1	0.758	0.803	0.731	0.773
Absolute ΔF1	+0.112	+0.061	+0.080	+0.049
Relative gain	17.3%	8.2%	12.3%	6.8%
Paired ΔF1, pointwise 95% CI	[0.075–0.150]	[0.033–0.092]	[0.064–0.096]	[0.034–0.064]
Paired ΔF1, familywise-adjusted CI	[0.060–0.166]	[0.018–0.103]	[0.057–0.103]	[0.027–0.072]

**Table 3:** Detection coverage, false-positive rate, and score-ranking performance.

Dataset	Labels	Method and decision rule	Precision	Recall	FPR, %	F1	AUROC	AUPRC
AIOps	1%	Proposed	0.880	0.665	0.512	0.758	0.934	0.805
AIOps	1%	ADS	0.780	0.551	0.877	0.646	0.899	0.706
AIOps	5%	Proposed	0.830	0.778	0.898	0.803	0.956	0.853
AIOps	5%	ADS	0.790	0.699	1.046	0.742	0.927	0.795
HDFS	1%	Proposed	0.860	0.636	0.313	0.731	0.946	0.789
HDFS	1%	DeepLog, calibrated	0.770	0.540	0.487	0.635	0.914	0.683
HDFS	1%	PLELog	0.790	0.553	0.444	0.651	0.920	0.709
HDFS	1%	DeepLog, fixed *g* = 9	0.577	0.730	1.612	0.645	0.914	0.683
HDFS	5%	Proposed	0.810	0.739	0.523	0.773	0.958	0.825
HDFS	5%	DeepLog, calibrated	0.800	0.661	0.499	0.724	0.943	0.779
HDFS	5%	PLELog	0.780	0.651	0.554	0.710	0.935	0.757
HDFS	5%	DeepLog, fixed *g* = 9	0.666	0.822	1.245	0.736	0.943	0.779

**Table 4:** Controlled comparison with recent deep-learning and foundation-model-based anomaly detectors under a common HDFS session-level protocol.

Method	Representation and detection model	Precision	Recall	F1
LogGPT	GPT-based generative event-sequence model	0.890 ± 0.030	0.920 ± 0.050	0.904 ± 0.031
LogLLM	BERT semantic encoder and Llama sequence classifier	0.993 ± 0.003	0.997 ± 0.002	0.995 ± 0.002
Llama3-ReFT	Llama 3 8B with representation fine-tuning	0.984 ± 0.007	0.989 ± 0.006	0.986 ± 0.005
**Proposed approach**	**Invariant-guided execution-regime geometry**	**0.910 ± 0.018**	**0.932 ± 0.020**	**0.921 ± 0.015**

**Table 5:** Component ablation under shared and retuned configurations.

Configuration	AIOps, 1%	AIOps, 5%	HDFS, 1%	HDFS, 5%
Full model	0.753 ± 0.010	0.800 ± 0.008	0.725 ± 0.015	0.769 ± 0.011
Without $R_{\text{inv}}$, shared configuration	0.715 ± 0.016	0.772 ± 0.010	0.675 ± 0.022	0.734 ± 0.015
Without $R_{\text{geom}}$, shared configuration	0.737 ± 0.019	0.788 ± 0.013	0.704 ± 0.026	0.753 ± 0.018
Uniform w, shared configuration	0.707 ± 0.018	0.765 ± 0.012	0.659 ± 0.025	0.721 ± 0.017
Without $R_{\text{inv}}$, retuned	0.720 ± 0.017	0.776 ± 0.011	0.684 ± 0.020	0.740 ± 0.016
Without $R_{\text{geom}}$, retuned	0.736 ± 0.020	0.792 ± 0.014	0.713 ± 0.023	0.752 ± 0.019
Uniform w, retuned	0.711 ± 0.019	0.770 ± 0.012	0.668 ± 0.023	0.727 ± 0.018

All weak-supervision methods receive the same disclosed development-label subset within each dataset and budget. The 1% and 5% values denote the class-wise label budgets. They do not specify test flagging quotas. HDFS methods score identical Block-ID sessions. AIOps methods score identical 60-sample windows with stride 15. A baseline prediction for a destination regime is attached to the corresponding evaluation record of the proposed change-based detector. Independent HDFS sessions are never concatenated to generate baseline event contexts.

The HDFS baselines use the supplied LogHub event templates and Block-ID traces already used by the proposed method. No additional parser is fitted or substituted. The benchmark template inventory is treated as a fixed preprocessing resource. TF–IDF statistics, semantic transforms, and detector parameters are estimated within the fitting partition. Test labels do not enter any fitting or calibration step. DeepLog and ADS are paper-guided reimplementations. PLELog is adapted from the authors’ public implementation.

DeepLog is trained by next-key cross-entropy on disclosed normal fitting sessions. Disclosed anomalous labels enter development selection and threshold calibration but are excluded from normal-sequence training. Contexts remain within their own session and are left-padded at session starts. Let J denote the fixed number of benchmark templates. The observed key receives its rank in the descending predicted-probability ordering of these templates. Probability ties are resolved by template ID. An unmatched key receives rank J+1. The session score is the maximum observed rank divided by J+1. The same denominator is used across folds. Thresholding this score retains the interpretation that a session is flagged when at least one event falls outside the accepted next-key candidates. The parameter-value and online-feedback components are excluded.

PLELog retains GloVe semantic embeddings, TF–IDF aggregation, FastICA, HDBSCAN probability estimation, and the attention-based GRU. Its clustering representation is the sum of semantic event vectors within each fitting session. Normal anchors and estimated probabilities are constructed from fitting records only. Clusters without a disclosed normal anchor retain the original preliminary anomalous interpretation. Unassigned HDBSCAN noise records receive anomaly probability 0.5. Disclosed fitting labels replace estimated probabilities for their own records. This use of disclosed anomalous labels is an explicit adaptation to the common class-wise budget. The GRU anomaly-class output supplies the session score.

ADS retains the detector-severity bank of 117 configurations described in [36], CPLE, and a random-forest base classifier. Each severity configuration supplies its mean and maximum over the evaluated KPI window. The resulting representation contains 234 severity coordinates. A further 117 indicators identify configurations with incomplete history within the window. Available severity values are aggregated without using window labels. An entirely unavailable aggregate is replaced by its fitting-data median. A coordinate unavailable throughout fitting is assigned zero and retains its missing-history indicator. Window labels supervise complete window vectors. They are not expanded into timestamp labels.

Calendar-based severity configurations retain their timestamp-based horizons. Detector histories are bounded at seven days and restarted at observation gaps. Parameters and reference statistics are estimated from fitting data. Cross-fitting applies a common purge to all compared KPI methods using the union of raw-data supports required by their descriptors. This includes severity histories and the complete support of overlapping regime windows. The development/test boundary and evaluation records remain fixed. Historical inputs available before a test window are used causally without updating the fitted detector.

The ADS shape-grouping component follows the normalisation, baseline extraction, shape-based distance, and density-clustering organisation of ROCKA. For each series, its longest uninterrupted fitting segment supplies the shape representation. Values are standardised, the 5% largest absolute deviations are replaced by interpolation within that segment, and a trailing 60-sample mean extracts the baseline. Baselines are resampled to 1,024 equally spaced relative-time positions and standardised again. DBSCAN uses shape-based distance with radius 0.2 and minimum samples 4. Noise series form singleton groups. Constant baselines have distance zero to one another and distance one to non-constant baselines. These shape-grouping settings are fixed across label budgets.

A source series is eligible for supervised transfer if its disclosed fitting windows contain both classes. Within each shape group, the representative is the eligible series minimising the sum of squared shape distances to group members. Ties are resolved by series ID. If the group contains no eligible source, its target series uses the nearest eligible source across groups. If no individual source contains both classes, the disclosed fitting pool supplies the supervised reference. For each target, CPLE combines the representative’s disclosed windows with the target’s own disclosed windows and unlabelled fitting windows. Duplicate records are included once. No additional representative-stream labels are disclosed.

Configuration selection uses five-fold development cross-fitting and F1 on pooled disclosed out-of-fold records. DeepLog searches context lengths {5,10,20}, hidden sizes {64,128}, and layer counts {1,2}. Adam uses learning rate 0.001, batch size 128, and 20 epochs. The selected 1% configuration has context length 10, hidden size 64, and two layers. The selected 5% configuration has context length 10, hidden size 128, and two layers.

PLELog searches GRU hidden sizes {50,100,150}, requested FastICA dimensions {10,20}, and HDBSCAN minimum cluster sizes {20,50,100}. The effective FastICA dimension is capped by the numerical rank of the centred fitting representation. HDBSCAN minimum samples equals its selected minimum cluster size. The GRU has one layer and uses Adam learning rate 0.002, batch size 64, and 20 epochs. Both budgets select hidden size 100 and effective FastICA dimension 20. Minimum cluster size is 100 at 1% and 50 at 5%.

ADS searches forest sizes of 100 and 300 trees, maximum depths of 10 and unrestricted depth, and minimum leaf sizes of 1 and 5. Both budgets select 300 trees and unrestricted depth. Minimum leaf size is 5 at 1% and 1 at 5%. CPLE stops at a relative objective change below $10^{-3}$ or after 20 iterations. Configuration ties favour lower model complexity and then a fixed configuration order. All baseline stochastic seeds are fixed at 42.

Each fitted method uses one nominal-reference CDF pooled over its disclosed normal fitting regimes. For ADS, this CDF is pooled across KPI series rather than estimated separately for each target stream. Decision cutoffs maximise F1 on pooled disclosed out-of-fold percentile scores. Ties favour the higher cutoff. Selected configurations are refitted on development data and their cutoffs are fixed before test evaluation. The selected DeepLog cutoffs correspond to accepted next-key ranks g=11 at 1% and g=10 at 5%. These are development-selected candidate ranks rather than test anomaly-rate constraints. The 20 repetitions varying spherical k-means initialisation do not represent independent neural-baseline retraining.

Published HDFS headline values concern different experimental configurations. DeepLog reported F1 of approximately 0.96 using about 4,855 normal training sessions [21]. Its successful use of a small training set means that label scarcity alone cannot explain the present difference. The benchmark preprocessing, selected training sessions, session-start treatment, and calibration differ. PLELog reported HDFS F1 of 0.957 with a much larger known-normal training fraction [22]. Its normal-anchor coverage therefore also differs from the present budgets. The individual contributions of these protocol differences are not identified by the headline comparison.

A diagnostic DeepLog control applies the original fixed g=9 candidate rule to the same fitted models and test sessions. It changes only the decision cutoff. Its F1 values are 0.645 and 0.736 at the 1% and 5% budgets. The corresponding development-calibrated values are 0.635 and 0.724. Restoring the fixed candidate rule therefore changes F1 by approximately 0.010 and 0.012. It does not recover the original paper’s headline performance. The diagnostic identifies cutoff selection as a limited contributor to the difference within these fitted models. It does not isolate preprocessing or training effects. The control is reported descriptively in Table 3 and does not enter configuration selection or the primary paired-comparison family.

The original ADS evaluation additionally used anomaly-segment adjustment and test-label selection of the best threshold [36]. The present evaluation uses fixed development-calibrated window decisions without segment adjustment. The quantitative claims consequently concern the specified common weak-supervision protocol. The within-framework ablations provide the more direct evidence concerning the contribution of the proposed mathematical components.

Figs 1–3 provide complementary diagnostic views of the HDFS execution-regime representation. Fig 1 examines neighbourhood organisation, Fig 2 adds invariant consistency, and Fig 3 displays pairwise geometry directly. Their common sample allows these properties to be examined together. Detection performance on HDFS and AIOps is evaluated separately using the complete held-out test sets. The figures use the final HDFS model from the 5% weak-supervision setting with k-means initialization seed 42. A fixed diagnostic sample contains 300 normal and 90 anomalous test regimes, selected uniformly without replacement within each class using sampling seed 2026. The resulting anomaly proportion is 90/390 = 23.1%. This deliberate overrepresentation makes anomalous patterns visible and does not reproduce the approximately 2.93% anomaly prevalence of the complete HDFS test set. Ground-truth test labels are used for diagnostic sampling, visual annotation, and subsequent descriptive calculations. They do not enter model fitting, model selection, or threshold calibration. Figs 1 and 2 use all 390 sampled regimes. Fig 3 uses a balanced subsample defined below. Geometric calculations use the regime vectors $\mu_{\delta_{r}}$ and the learned feature weights of the selected model. Under q=2, the weighted coordinates $x_{r}^{\left(i\right)}=\sqrt{w_{i}}\mu_{\delta_{r}}^{\left(i\right)}$ satisfy ${\|x_{r}-x_{s}\|}_{\text{2}}=d_{w}\left(\mu_{\delta_{r}},\mu_{\delta_{s}}\right)$. Distances are calculated in this original weighted space. Concentration statistics use assignments from the fitted clustering model. The projections provide visual coordinates and do not redefine either the pseudometric or cluster membership.

**Fig 1:**
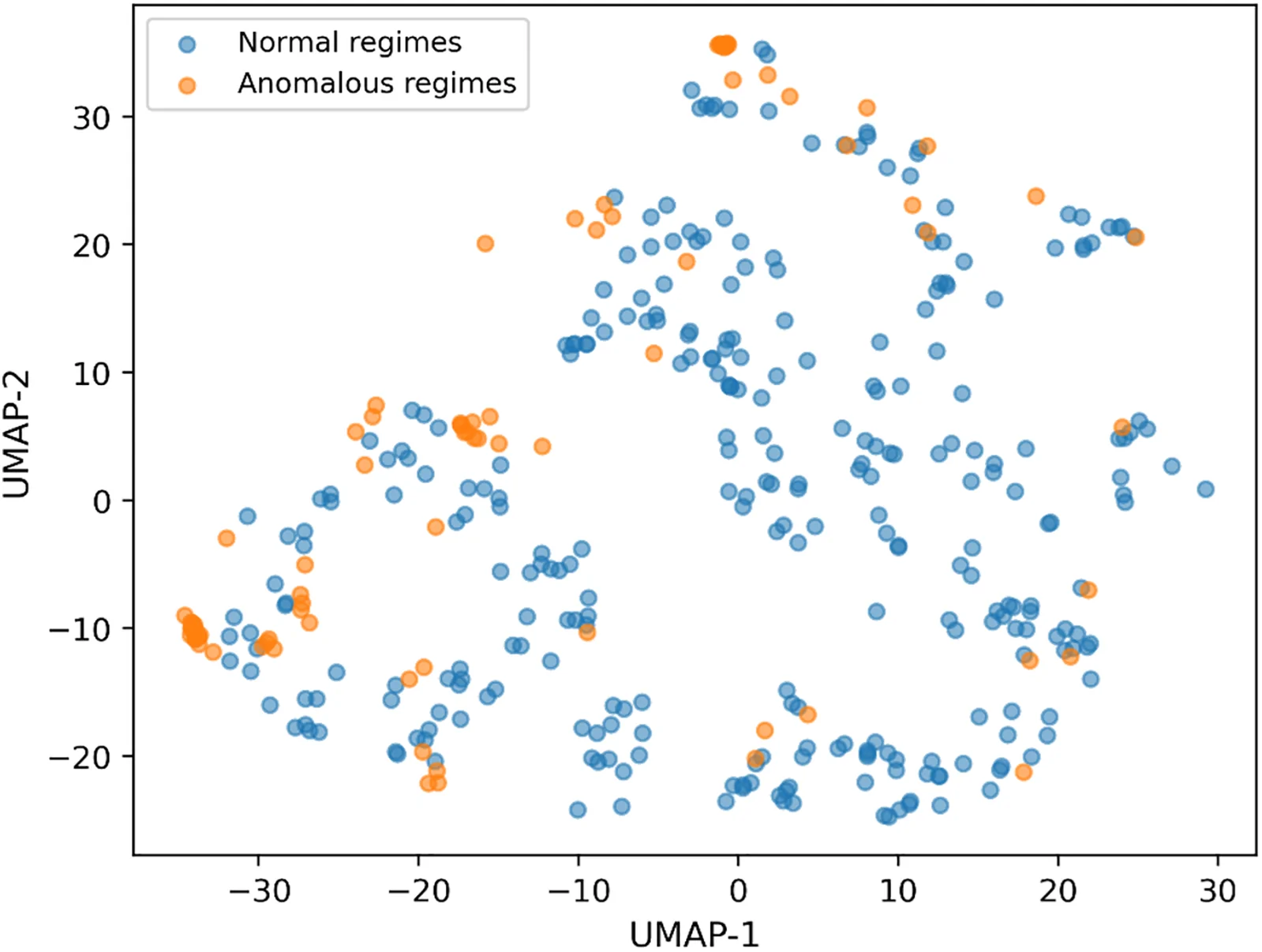
Two-dimensional projection of the HDFS diagnostic sample.

**Fig 2:**
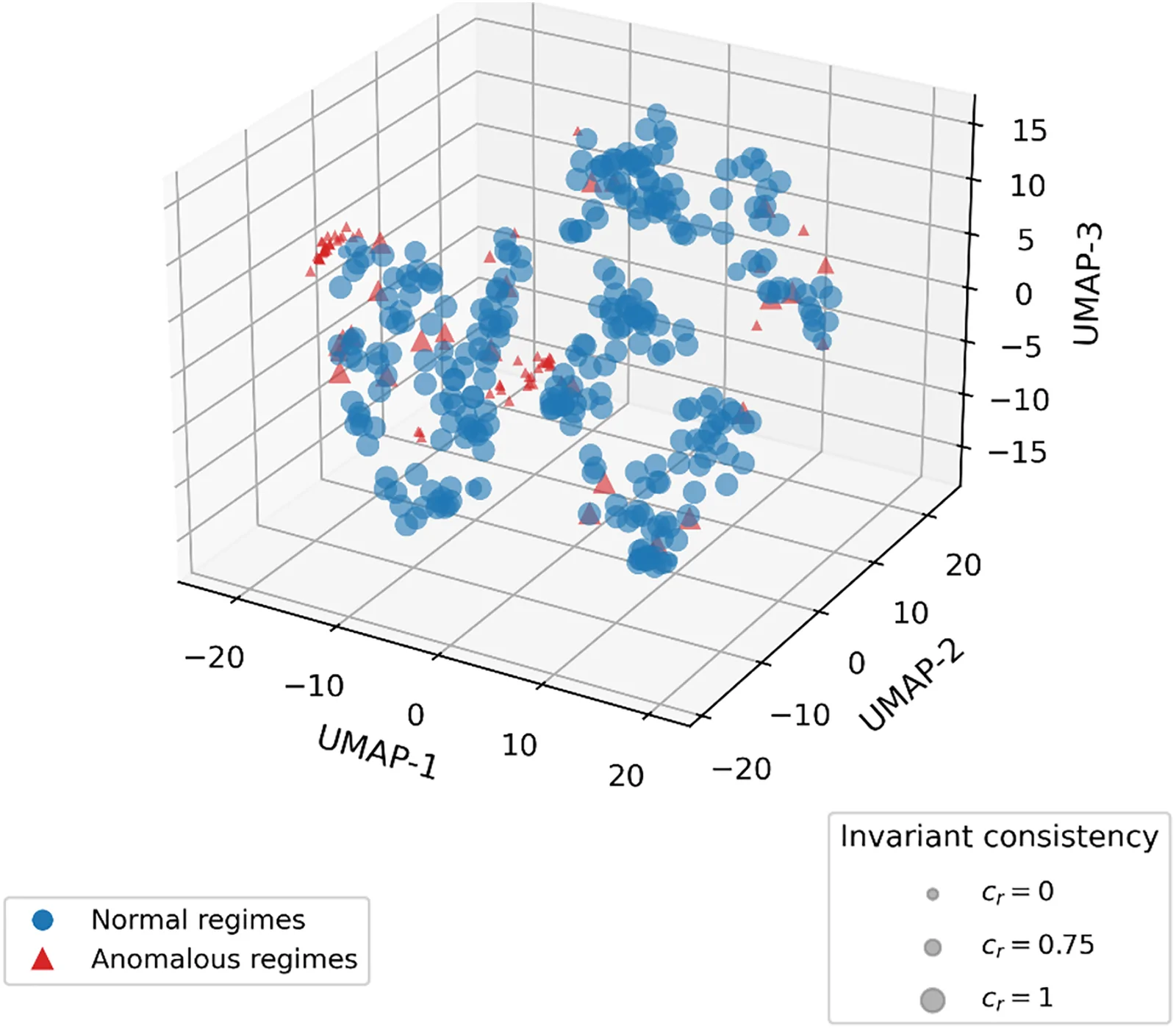
HDFS diagnostic regimes with invariant-consistency encoding.

**Fig 3:**
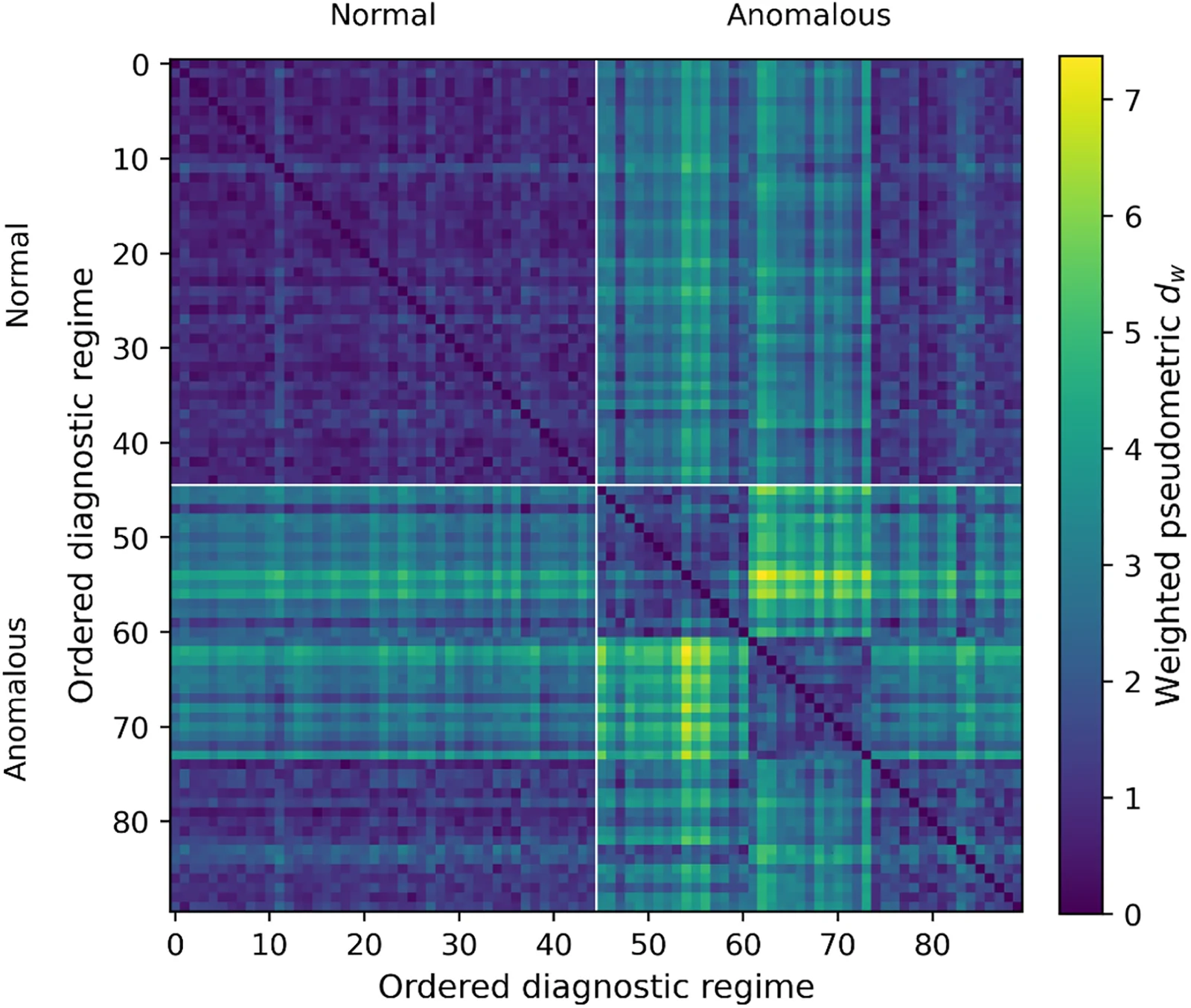
Weighted-pseudometric matrix for a balanced HDFS diagnostic subsample.

Fig 1 shows a two-dimensional UMAP projection of the weighted regime coordinates. A reference set of 600 development regimes is sampled uniformly without replacement using seed 42. The fixed reference size limits the computational cost of the full-pair embedding objective. It is used only to construct the visualisation. The detector remains fitted to the development data specified in the evaluation protocol.

The reference set includes members of all six fitted HDFS clusters. This coverage connects the projection to the model’s operational groups without implying exhaustive coverage of every rare execution pattern. The embedding is fitted to the reference set, and the diagnostic test regimes are subsequently transformed with the reference coordinates held fixed.

The implementation uses a UMAP fuzzy neighbourhood graph and low-dimensional cross-entropy objective, optimized with L-BFGS-B. It is a custom implementation rather than the reference umap-learn package. The settings are 15 neighbours, a minimum distance of 0.1, two output dimensions, Euclidean input distance, and fitting and transformation seeds of 42. Spectral initialization is used for the reference embedding. Optimization permits up to 650 fitting iterations and 400 transformation iterations, with a relative objective tolerance of $10^{-6}$. These settings are fixed before examining the diagnostic test labels. Normal regimes are represented by blue circles and anomalous regimes by orange circles. The axes are labelled UMAP-1 and UMAP-2 and have no direct physical interpretation.

The numerical analysis distinguishes fitted cluster membership from apparent separation in the projection. Let N and A denote the sampled normal and anomalous sets. The two fitted clusters containing the largest numbers of sampled anomalies are denoted $C_{a}$ and $C_{b}$, with ties resolved by cluster identifier. They are selected after fitting for descriptive analysis and are not defined by drawing boundaries in the embedding. Concentration is calculated separately within each operational class, $p_{A}=\frac{\left|A\cap \left(C_{a}\cup C_{b}\right)\right|}{\left|A\right|}$, $\text{\textbackslash{}hspace\{0.5em}\}p_{N}=\frac{\left|N\cap \left(C_{a}\cup C_{b}\right)\right|}{\left|N\right|}$. The ratio $p_{A}/p_{N}$ describes enrichment in these selected groups.

Since the groups are selected using anomaly counts, this ratio is a descriptive summary rather than an independent performance measure or significance test. Geometric displacement is measured in the original weighted space, ${\overset{―}{x}}_{N}=\frac{\text{1}}{\left|N\right|}\sum_{r\in N}x_{r}$, $\text{\textbackslash{}hspace\{0.5em}\}{\overset{―}{x}}_{A}=\frac{\text{1}}{\left|A\right|}\sum_{r\in A}x_{r}$, $D={\|{\overset{―}{x}}_{A}-{\overset{―}{x}}_{N}\|}_{\text{2}}$, $R_{N}=\frac{\text{1}}{\left|N\right|}\sum_{r\in N}{\|x_{r}-{\overset{―}{x}}_{N}\|}_{\text{2}}$. The ratio $D/R_{N}$ expresses class-centroid displacement relative to the mean spatial radius of sampled normal regimes.

The two selected clusters contain 36 and 24 anomalous regimes and 50 and 46 normal regimes, respectively. They therefore account for $p_{A}=\left(36+24\right)/90=66.7\%$ of sampled anomalies and $p_{N}=\left(50+46\right)/300=32.0\%$ of sampled normal regimes. The concentration ratio is $p_{A}/p_{N}=2.08$. Thus, approximately two thirds of the sampled anomalies occur in operational groups containing approximately one third of the sampled normal class. The selected groups remain mixed. Their enrichment describes where anomalies occur within the fitted operational partition rather than identifying anomaly-only clusters.

The class-centroid displacement is D=1.04, and the mean normal radius is $R_{N}=0.70$. Their ratio is $D/R_{N}=1.04/0.70=1.49$, or approximately one and a half normal radii. The absolute values are expressed in the weighted representation units fixed by development preprocessing. The ratio is unchanged by a common positive rescaling of those coordinates and is independent of projection distortion.

Concentration and displacement describe different aspects of abnormal execution. Concentration indicates that particular feature configurations recur preferentially within a subset of operational groups. Displacement indicates a shift in the average weighted representation of the anomalous class. Neither observation requires anomalous regimes to form isolated regions in UMAP. Mixed memberships are consistent with abnormal executions sharing substantial structure with admissible executions. The diagnostic summaries therefore explain the relevance of operational context without equating projected separation with detection accuracy.

Fig 2 adds invariant consistency to a three-dimensional view of the same HDFS diagnostic sample. A separate three-dimensional UMAP embedding is fitted to the same development reference set using the implementation and settings specified for Fig 1, with the number of output dimensions changed to three. The axes are labelled UMAP-1, UMAP-2, and UMAP-3. Normal regimes are shown as blue circles and anomalous regimes as red triangles.

Invariant consistency is calculated from the three HDFS violation functionals, $c_{r}=1-\frac{\text{1}}{\text{3}}\sum_{m=1}^{\text{3}}v_{m}\left(\delta_{r}\right)$. The bounded violations defined above ensure $0\leq c_{r}\leq 1$. Larger values indicate closer agreement with the selected nominal constraints. Marker area is set to $s_{r}=12+48c_{r}^{\text{4}}$ square points. The mapping retains a visible minimum marker size and emphasizes differences near the nominal-consistency range. The legend includes grey reference markers for $c_{r}=0,0\mathrm{.75}$, and 1. Marker opacity is held constant and depth shading is disabled, making marker area the sole consistency encoding. For the numerical analysis, consistency comparable to nominal behaviour is defined by $c_{r}\geq t_{c}$. The reference $t_{c}$ is the fifth percentile of consistency among the disclosed normal development regimes. It is estimated before examining the test sample and is used only for this diagnostic interpretation. It does not replace the detector’s calibrated decision threshold.

The development-derived consistency reference is $t_{c}=0.9561$. In the diagnostic sample, 18 of 90 anomalous regimes satisfy $c_{r}\geq t_{c}$, giving 18/90 = 20.0%. This calculation supports the observation that approximately one fifth of sampled anomalies retain consistency comparable to nominal behaviour.

Template-occurrence, frequency, and composition relationships constrain specific aspects of execution. Agreement with these relationships does not determine whether the complete execution is operationally normal. An anomalous regime can preserve the selected relationships while exhibiting an unusual combination of otherwise admissible features. Reduced consistency, in turn, identifies departures from the selected constraints without identifying a particular failure mechanism.

The separate encodings of operational class and consistency make this distinction explicit. The 18 high-consistency anomalies show that an invariant-consistency cutoff alone would not identify every labelled anomaly in this sample. They provide a concrete rationale for combining invariant evidence with geometric and cluster context in the quality functional (11). Whether the combined detector identifies these regimes, and how much each component contributes, is established by the prediction results and ablation analysis.

Fig 3 examines pairwise geometry directly without using embedding coordinates. For readability, 45 normal and 45 anomalous regimes are selected uniformly without replacement from the diagnostic sample using sampling seed 2027. Normal regimes precede anomalous regimes, with record identifiers determining the ordering within each class. Identical ordering is applied to rows and columns. The two equally sized diagonal regions therefore correspond to deliberately balanced display groups and do not represent HDFS anomaly prevalence or the sizes of fitted clusters.

Each matrix entry is calculated from the original regime vectors and the selected feature weights, $D_{rs}=d_{w}\left(\mu_{\delta_{r}},\mu_{\delta_{s}}\right)={\left(\sum_{i=1}^{p}w_{i}|\mu_{\delta_{r}}^{\left(i\right)}-\mu_{\delta_{s}}^{\left(i\right)}{|}^{\text{2}}\right)}^{1/2}$. Every unordered pair is evaluated once and its value is entered in both symmetric positions. Diagonal entries are zero by definition. Nonnegative weights ensure the pseudometric properties, including the triangle inequality. Distinct vectors can have zero distance when their differences occur only in zero-weight coordinates.

The matrix is checked for a maximum diagonal magnitude and maximum absolute asymmetry below $10^{-12}$. No independent cell perturbations, row-wise normalisation, or cell-wise clipping are applied. The colour bar displays unmodified $d_{w}$ values in the same weighted representation units used in the preceding analysis. A line marks the boundary between the normal and anomalous display groups.

Distance summaries are calculated separately for normal–normal, anomalous–anomalous, and normal–anomalous pairs. Within-class summaries use unordered pairs of distinct regimes and exclude diagonal entries. Each within-class group contains $\left(\begin{array}{c} @c@45\\ 2 \end{array}\right)=990$ pairs. The between-class group contains 45×45=2,025 pairs. These summaries are descriptive because distances sharing a regime are dependent.

Residual cross-class proximity is measured by $\omega =\frac{\#\{(r,sinN_{45}\times A_{45}:D_{rs}\leq Q_{0.75}^{NN}\}}{45\times 45}$, where $N_{45}$ and $A_{45}$ are the two displayed class groups and $Q_{0.75}^{NN}$ is the upper quartile of their normal–normal distances. This statistic measures how often a cross-class pair falls within the central distance scale of normal executions.

The regenerated matrix has a zero diagonal and is symmetric within the stated numerical tolerance. The interquartile ranges are 0.63–1.16 for normal–normal distances, 1.77–3.73 for anomalous–anomalous distances, and 1.40–2.98 for normal–anomalous distances. The central normal–normal and normal–anomalous ranges are separated. Nevertheless, ω=17.6% of normal–anomalous distances do not exceed the upper quartile of normal–normal distances. Separation of the central ranges therefore coexists with residual cross-class proximity.

The anomalous–anomalous range extends above the normal–anomalous range. Anomalous regimes are consequently not represented as a single compact class. Different deviations in feature occurrence, frequency, or composition can place two anomalous executions farther apart than an anomalous and a normal execution. This geometric heterogeneity does not identify the underlying failure mechanisms, but it explains why an operational anomaly label need not correspond to one coherent region of the representation.

The matrix and the preceding concentration analysis address different levels of organisation. Fig 3 summarizes distances across operational classes. The concentration calculation concerns association with fitted clusters, each of which can contain both classes. A dispersed anomalous class can therefore coexist with preferential membership in a small number of operational groups.

The equal dimensions of the displayed groups follow from balanced diagnostic sampling. They carry no implication that half of HDFS sessions are anomalous. The observed cross-class proximity motivates interpreting distances relative to operational neighbourhoods. Detection performance and the contribution of invariant and geometric regularisation remain established by the complete-test results and ablation analysis.

Fig 4 evaluates how annotation availability affects execution-regime detection across the two telemetry modalities. Panels 4a and 4c present F1 and precision for AIOps KPI data, comparing the proposed method with ADS; panels 4b and 4d present the corresponding HDFS results, comparing the proposed method with DeepLog and PLELog. The horizontal axis denotes the disclosed development-label budget defined above. Only the evaluated 1% and 5% settings are included; connecting segments identify the methods and link these settings without implying measurements at intermediate budgets.

**Fig 4:**
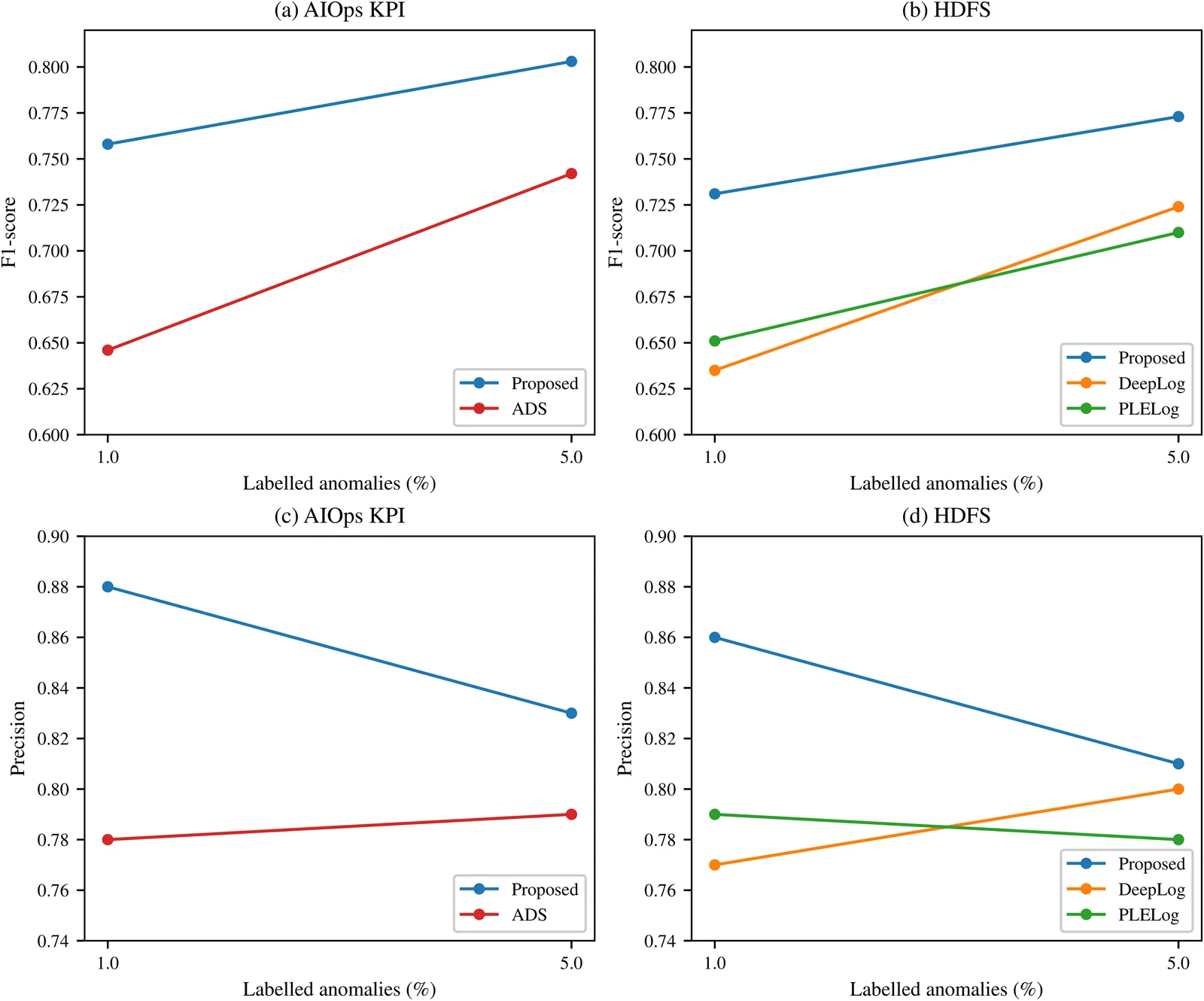
Execution-regime detection under limited supervision: AIOps F1 (a), HDFS F1 (b), AIOps precision (c), and HDFS precision (d).

The plotted values are calculated directly from the reference-initialisation predictions on the complete test manifests using the common regime-level evaluator. Each point represents observed test F1 or precision for the corresponding dataset, method, and annotation budget. The F1 values agree with Table 2 at the reported three-decimal precision. The proposed-method values are supported by the confusion matrices reported below. Bootstrap resampling is used separately to estimate uncertainty in between-method F1 differences. No dataset pooling, point adjustment, or separate recalibration is introduced for the figure.

The panels use consistent method colours and circular markers, separate legends for each dataset, and identical vertical scales within each metric. ADS is shown only for KPI data, and DeepLog and PLELog only for HDFS. Table 2 reports paired confidence intervals for F1 differences between the proposed method and the strongest applicable baseline in each setting. These intervals concern between-method differences and are therefore not displayed as error bars around the individual curves.

Fig 4 shows that annotation scarcity affects representation quality and decision coverage differently. Increasing supervision raises F1 for the proposed method on both datasets, although precision decreases. The additional labels therefore improve the balance between recovered anomalies and false alarms rather than increasing precision alone. This behaviour is consistent with a detector whose feature weights and regime geometry are calibrated jointly with a decision threshold: the annotation budget changes which departures are recognised, while the resulting alert fraction remains an independently observed quantity.

The advantage of the proposed method persists across both telemetry modalities, but its magnitude depends on the information available to the competing representations. KPI regimes are represented through coordinated level, slope, variability, and inter-window-change descriptors, whereas HDFS regimes retain event-template composition and structural variation. Consequently, the comparison tests whether explicit regime organisation remains useful under sparse labels in two different observation spaces. The detection metrics are computed from these original representations, not from the low-dimensional projections in Figs 1–3.

On AIOps, the proposed method achieves F1 values of 0.758 and 0.803 at 1% and 5% supervision, respectively, compared with 0.646 and 0.742 for ADS. Reducing the annotation budget therefore decreases F1 by 0.045 for the proposed method and by 0.096 for ADS, increasing the between-method difference from 0.061 to 0.112. This pattern is consistent with the role of regime-level constraints in KPI analysis: coordinated changes across several window descriptors can retain an identifiable structure even when few anomalous windows are labelled. The result supports the usefulness of the combined representation under annotation scarcity; the separate contributions of invariant regularisation, geometric regularisation, and learned weights require the component-wise ablation analysis.

On HDFS, the proposed method achieves F1 values of 0.731 and 0.773 at 1% and 5% supervision. DeepLog achieves 0.635 and 0.724, while PLELog achieves 0.651 and 0.710. The strongest baseline consequently changes from PLELog at 1% to DeepLog at 5%, matching Table 2. This ordering is consistent with the different treatment of scarce supervision: probabilistic label estimation can support log-sequence discrimination when confirmed anomalies are few, whereas increased annotation availability improves the calibration of sequence-based detection. The proposed method retains an advantage of 0.080 and 0.049 over the strongest baseline at the respective budgets, indicating that event-composition and regime-level organisation preserve useful anomaly information beyond the baseline sequence representations. The smaller advantage at 5% reflects stronger baseline performance rather than a reversal of the proposed method’s benefit.

A separate experiment tests sensitivity to the composition of the disclosed weak labels. Five additional class-specific random orderings are generated using seeds 101–105. Each ordering produces a 1% subset nested within its corresponding 5% subset, with the annotation counts unchanged. Every subset is evaluated using four common initialisation seeds, 42–45. The development and test manifests, cross-fitting folds, feature definitions, search ranges, and stopping criteria remain fixed. Representation calibration, model selection, and decision calibration are repeated for every configuration. All applicable baselines use the same disclosed subsets. The experiment therefore adds 20 complete evaluations per method, dataset, and annotation budget. No subset or initialisation is selected according to test performance.

Test F1 is first averaged over the four initialisations within each subset. The reported mean and sample standard deviation are then calculated across the five subset averages. This dispersion measures variability between annotation subsets after averaging over initialisations. It retains a residual initialisation contribution and is not interpreted as a pure variance component attributable to label selection. Baseline comparisons are paired within each subset. The strongest baseline is determined separately for each subset from its initialisation-averaged F1.

On AIOps, the proposed method achieves 0.752±0.018 at 1% supervision and 0.799±0.011 at 5%. The corresponding ADS averages are 0.646 and 0.741. On HDFS, the proposed-method results are 0.724±0.025 and 0.768±0.016. DeepLog averages 0.637 and 0.724, while PLELog averages 0.651 and 0.712. The minimum paired F1 advantages over the strongest applicable baseline are 0.079 and 0.039 on AIOps, and 0.047 and 0.027 on HDFS, at 1% and 5% supervision, respectively. These results are reported separately from the fixed-subset comparison in Fig 4 and Table 2.

The larger dispersion at 1% supervision indicates that the identity of the disclosed regimes remains consequential. This pattern is consistent with uneven coverage of anomalous regime configurations by a small annotation sample. The greater HDFS dispersion is also consistent with differences in event composition among labelled sessions, although failure-type coverage is not measured separately. The strongest HDFS baseline changes in some subsets, while the proposed method retains a positive paired advantage in every tested subset. Its benefit therefore does not depend on preserving a particular baseline ranking or selecting one favourable weak-label composition.

The proposed-method precision estimates in panels 4c and 4d decrease from 0.880 to 0.830 on AIOps and from 0.860 to 0.810 on HDFS as supervision increases from 1% to 5%. The reference confusion matrices clarify the operational meaning of this change: recall increases from 0.665 to 0.778 and from 0.636 to 0.739, respectively, while the alert fractions increase from 4.035% to 5.000% and from 2.165% to 2.673%. At 1%, the detector identifies a smaller subset with a higher proportion of true anomalies; at 5%, it recovers additional anomalies at the cost of admitting more nominal regimes. In production monitoring, these settings therefore represent different achieved balances between incident coverage and investigation workload. Higher precision under fewer labels does not indicate a better overall representation, because the corresponding F1 and recall are lower. The baseline precision trends remain method-specific.

Statistical comparison evaluates the difference in test F1 between completed development fits. Each evaluation record contains its regime identifier, original transition constituents, ground-truth label, and predictions from all applicable methods. Point estimates are calculated on the complete test manifest. At the reported three-decimal precision, they agree with the descriptive summaries in Fig 4. Bootstrap replicate b uses identical sampled identifiers and multiplicities for both methods. The paired difference is $\Delta F_{\text{1}}^{\left(b\right)}=F_{\text{1}}\;\left(y^{\left(b\right)},{\hat{y}}_{\text{proposed}}^{\left(b\right)}\right)-F_{\text{1}}\;\left(y^{\left(b\right)},{\hat{y}}_{\text{baseline}}^{\left(b\right)}\right)$. All feature mappings, weights, prototypes, anomaly scores, and development-calibrated thresholds remain fixed. The bootstrap therefore evaluates test-sample uncertainty conditional on the completed fits. It does not reinterpret resampled test labels as training information.

Paired moving-block bootstrap uses 10,000 replicates. Blocks retain consecutive evaluation records in the order used to construct the original regime changes. For KPI data, resampling is performed separately within each series and uninterrupted test segment. For HDFS, the original source and destination session identifiers remain attached to each transition record. Consecutive records can share constituent sessions, so the induced dependence is retained within sampled blocks. No new transition is constructed between resampled blocks.

Block lengths are determined from development data before examining test-label comparisons. The minimum dependence span accounts for shared raw observations. For the 60-sample KPI windows with a 15-sample stride, changes between consecutive windows can share observations through lag four. For adjacent HDFS session differences, neighbouring transitions share a constituent session, giving a minimum span of one lag.

Additional dependence is assessed from the development out-of-fold anomaly-score and binary-decision sequences of the compared methods. Autocorrelations are calculated within uninterrupted sequences. Constant sequences are omitted. Lags are examined up to the smaller of 100 and one tenth of the sequence length. Let q be the largest lag with absolute autocorrelation exceeding 0.10, subject to the minimum span imposed by shared observations. The dataset-level value is the maximum over the applicable methods, annotation budgets, and sequences. Block length is L=2(q+1). This rule gives q=9 and L=20 for KPI data, and q=15 and L=32 for HDFS.

Blocks are sampled uniformly from all admissible starting positions within each test segment. Sampling continues until its original record count is reached, and the last block is truncated when necessary. Segments shorter than L are resampled as complete segments. Series boundaries and manifest gaps are never crossed. Labels remain attached to sampled records, and the anomalous fraction is allowed to vary between replicates. Identical sampled records are used for every method.

Pointwise 95% intervals use the 0.025 and 0.975 quantiles of the paired difference distribution. The comparison family contains six comparisons. These are ADS at both AIOps budgets and DeepLog and PLELog at both HDFS budgets. Bonferroni-adjusted intervals use quantiles 0.05/(2×6) and 1−0.05/(2×6), providing nominal 95% familywise coverage. Table 2 displays the strongest baseline identified in the original comparison for each setting. Baseline identities are fixed during bootstrap resampling.

Block-length sensitivity is evaluated at L/2, L, and 2L. This check does not change the development-selected primary length. The same bootstrap index sets are used across methods and annotation budgets within each dataset.

Bootstrap replicates are used to estimate uncertainty, not as independent experimental observations for a Wilcoxon test. Cliff’s δ and qualitative effect-size categories are replaced by the absolute F1 difference and relative gain. Sensitivity to recalibration is reported separately through the 20 initialisation repetitions, the weak-label-subset experiment, and the component ablation in Table 5.

Table 2 reports observed test F1 for the reference fits and paired-bootstrap intervals for the corresponding between-method differences. Its point estimates are calculated on the complete test manifests using the same predictions and evaluator as Fig 4. ADS is the strongest AIOps baseline at both annotation budgets. PLELog and DeepLog are the strongest HDFS baselines at 1% and 5%, respectively. Absolute improvement is the proposed-method F1 minus the corresponding baseline F1. Relative gain is this difference divided by baseline F1 and expressed as a percentage. Both gains are calculated before rounding. The pointwise and familywise-adjusted intervals estimate test-sample uncertainty conditional on the completed fits and fixed thresholds. Sensitivity to representation fitting and model selection is assessed separately through the initialisation repetitions, weak-label-subset experiment, and component ablation.

To make the fitted-model results directly auditable, the confusion matrices below report predictions from the reference k-means initialisation, with seed 42, on the unchanged test sets before bootstrap resampling. Rows correspond to actual nominal and anomalous regimes, and columns to predicted nominal and anomalous regimes; each matrix therefore has the form $\left(\begin{array}{cc} @cc@TN & FP\\ FN & TP \end{array}\right)$. These counts describe one specified fitted-model evaluation and are distinguished from the across-initialisation variability and bootstrap estimates in Table 2.

For HDFS, the matrices are $C_{\text{HDFS},1\%}=\left(\begin{array}{cc} @cc@\text{111296} & \text{349}\\ \text{1227} & \text{2141} \end{array}\right)$, $C_{\text{HDFS},5\%}=\left(\begin{array}{cc} @cc@\text{111061} & \text{584}\\ \text{878} & \text{2490} \end{array}\right)$. For AIOps, the matrices are $C_{\text{AIOps},1\%}=\left(\begin{array}{cc} @cc@\text{18836} & \text{97}\\ \text{357} & \text{710} \end{array}\right)$, $C_{\text{AIOps},5\%}=\left(\begin{array}{cc} @cc@\text{18763} & \text{170}\\ \text{237} & \text{830} \end{array}\right)$. The resulting HDFS F1 scores are 0.731 and 0.773 at 1% and 5% supervision, respectively; the AIOps scores are 0.758 and 0.803. The alert fractions are calculated as $(TP+FP)/N_{\text{test}}$, while prevalence is calculated as $(TP+FN)/N_{\text{test}}$. Consequently, prevalence remains fixed across supervision settings even though the alert fraction and recall change.

The paired F1 differences are positive in all four displayed settings, and their familywise-adjusted intervals exclude zero. This conclusion is based on the distribution of each difference rather than on overlap between separate marginal intervals. Shared difficult regimes can affect both methods, so pairing preserves information relevant to uncertainty in their comparison. The intervals condition on the completed development fits and thresholds. They do not measure variation from refitting the representation. That variation is addressed by the separate initialisation, weak-label-subset, and ablation experiments. These analyses distinguish test-sample uncertainty from sensitivity of the learning procedure.

The largest observed advantage occurs on AIOps at 1% supervision. The difference is 0.112 F1, with a familywise-adjusted interval of [0.060–0.166]. This pattern is consistent with nominal-reference constraints supporting discrimination when disclosed labels are sparse. The representation combines changes in level, trend, and variability within each KPI series. The invariant ablation in Table 5 provides complementary evidence that these constraints contribute to the complete calibration procedure.

The smallest observed advantage occurs on HDFS at 5% supervision. The difference against DeepLog is 0.049 F1, with a familywise-adjusted interval of [0.027–0.072]. Greater label availability narrows the gap while retaining a positive difference. This is consistent with sequential and regime-composition representations sharing part of the anomaly information available in event traces. The remaining advantage concerns the integrated representation rather than an isolated contribution of a particular invariant family.

Observed improvements range from 0.049 to 0.112 F1. Their median is 0.071, and the median relative gain is 10.3%. These summaries describe four evaluated settings rather than independent samples from a population of datasets. The larger gains at 1% supervision are consistent with the contribution of nominal-reference constraints under scarce labels. At half and twice the development-selected block lengths, the four displayed comparisons retain positive familywise-adjusted lower bounds. This supports stability of the conclusion to the specified resampling-scale alternatives. The primary block lengths remain those selected before test comparison.

The comparisons use common test manifests and development annotation budgets for methods applicable to each dataset. Their paired intervals support positive F1 differences after adjustment for the six-comparison family. Absolute and relative gains describe the magnitude directly. Operational consequences additionally depend on recall, false-positive rates, and the costs of missed and false alerts. The methodological contribution combines the observed detection advantage with explicit regime geometry and nominal-reference-guided metric calibration.

Table 3 complements the paired F1 differences with detection coverage, false-positive burden, and score-ranking performance. It uses the same seed-42 fitted models and test records as the primary comparisons in Table 2. Precision, recall, F1, and false-positive rate use the fixed decision cutoffs. Recall is TP/(TP+FN), and false-positive rate is FP/(FP+TN). AUROC uses the unthresholded native regime scores and trapezoidal ROC integration. AUPRC is reported as average precision, computed as the sum of precision weighted by each increase in recall. Equal scores are processed together. Ranking metrics are calculated before nominal-percentile transformation. The pooled nominal CDF does not reverse score ordering between KPI series. AIOps metrics pool all test windows across the 29 series. No point or anomaly-segment adjustment is applied. Fixed-g=9 DeepLog rows reuse the corresponding fitted scores and differ only in their cutoff. Values are rounded after calculation.

On AIOps, the proposed method increases recall and reduces false-positive rate relative to ADS at both budgets. Its F1 advantage combines greater anomaly coverage with fewer false alerts. On HDFS at 1%, comparison with PLELog gives 277 additional true positives and 147 fewer false positives. At 5%, comparison with calibrated DeepLog gives 263 additional true positives and 27 additional false positives. The latter improvement reflects increased anomaly coverage with a limited increase in normal sessions flagged. It does not imply uniformly lower false-positive rates.

The fixed g=9 DeepLog control increases recall relative to its development-calibrated decision rule. At 1%, this adds 638 true positives and 1,256 false positives. At 5%, it adds 543 true positives and 833 false positives. These additional false alerts constrain the resulting F1 improvement despite the larger anomaly coverage. The proposed method retains higher F1 than both DeepLog decision variants. Its advantage over the fixed-g=9 control is associated with a more favourable balance between detection coverage and false-positive burden.

The relevance of false-positive burden follows from the low anomaly prevalence. Precision satisfies $P=\frac{\pi R}{\pi R+(1-\pi textFPR}$, where π is the test anomaly rate and FPR is expressed as a fraction. When normal regimes dominate, a small increase in FPR can substantially change the composition of the alert set. Reporting recall and FPR separately therefore reveals an operational trade-off that F1 alone does not describe.

The higher AUPRC values of the proposed method indicate better aggregate precision–recall ranking under the observed class imbalance. They do not imply that its PR curve dominates at every cutoff. AUROC gives the same method ordering. The unchanged ranking metrics in the two DeepLog decision variants confirm that their detection differences arise from cutoff selection rather than different fitted representations. Together with the component ablations in Table 5, these results support the role of calibrated regime geometry while distinguishing ranking quality from the selected operating point.

To extend the comparison beyond the methodologically related baselines considered above, an additional controlled HDFS experiment was conducted with recent deep-learning and foundation-model-based log anomaly detectors. Unlike the 1% and 5% weak-supervision settings, which restrict the development labels available for model construction and cross-fitted decision calibration, this experiment uses complete development labels and a separate validation partition under a common HDFS session-level protocol. The two settings therefore assess complementary aspects of the framework: performance under annotation scarcity and discriminative capacity with complete development supervision.

The experiment used the HDFS_v1 corpus distributed through LogHub. Log records were grouped by Block ID into execution sessions while preserving their temporal order; records without a valid Block ID were excluded, and events referring to multiple valid Block IDs were assigned to the corresponding sessions. The resulting corpus contained 575061 sessions, comprising 558223 nominal and 16838 anomalous sessions. Partitioning was performed at the Block-ID level before any model-specific preprocessing and stratified by the binary session label. With random seed 42, the corpus was divided into 72% fitting, 8% validation, and 20% test partitions containing 414043, 46005, and 115013 sessions, respectively. Their nominal/anomalous compositions were 401920/12123, 44658/1347, and 111645/3368. The same Block-ID split manifest was used by every method, so performance differences are not attributable to differences in test-session membership or class prevalence.

The fitting partition was used for model fitting, representation calibration, and parameter estimation. Validation data were used for hyperparameter selection, early stopping where required, and decision calibration. The test partition remained untouched until final evaluation. No test labels entered feature construction, weight estimation, clustering, neural adaptation, hyperparameter selection, or threshold determination. Three recent neural baselines were evaluated from their reference implementations. LogGPT [28] retained its GPT-based next-event modelling and reinforcement-learning adaptation. LogLLM [41] retained its BERT semantic encoder, projection module, and Llama sequence classifier. Llama3-ReFT retained the Llama 3 8B backbone and representation fine-tuning procedure from the LogADReFT implementation of Lim et al. [42]. Apart from enforcing the common Block-ID split, method-specific preprocessing, pretrained checkpoints, and reference hyperparameters were preserved.

Internal representations were intentionally left method-specific. LogGPT operated on event sequences, LogLLM and Llama3-ReFT on their native semantic language-model representations, and the proposed framework on the event-template, directed structural-change, weighted-geometry, and invariant representation defined in Sect 2. Thus, the controlled quantities were the underlying sessions, partition membership, labels, evaluation unit, and metrics rather than the internal representation itself. For the proposed framework, q=2, $k_{nn}=10$, and the equal ρ, η, and γ coefficients were retained from the principal experiments, whereas k, $\lambda_{\text{1}}$, $\lambda_{\text{2}}$, and w were recalibrated using only the fitting and validation partitions according to the model-selection procedure defined above.

All predictions were assessed by a common external session-level evaluator with anomalous sessions treated as the positive class. Precision, Recall, and F1 were calculated as $P=\frac{TP}{TP+FP}$, $R=\frac{TP}{TP+FN}$, $\text{\textbackslash{}hspace\{0.5em}\}F1=\frac{2PR}{P+R}$. A common evaluator was necessary because the reference implementations do not all use identical internal averaging conventions. Each method therefore produced one binary decision per Block ID for final evaluation. For the proposed continuous anomaly score, the decision threshold was selected exclusively on the validation partition by maximising validation F1 and then fixed for the test partition. Each complete experiment was repeated for 3 independent random seeds while retaining identical data partitions; Table 4 reports mean values and standard deviations across repetitions.

The controlled experiment reveals a systematic relationship between detection performance and representational capacity. The proposed approach achieves an F1 score of 0.921 ± 0.015, compared with 0.904 ± 0.031 for LogGPT, 0.995 ± 0.002 for LogLLM, and 0.986 ± 0.005 for Llama3-ReFT. It therefore exceeds LogGPT by 0.017 F1 while trailing LogLLM and Llama3-ReFT by 0.074 and 0.065, respectively. This ordering is consistent with the information retained by the competing representations. LogLLM and Llama3-ReFT preserve high-capacity pretrained semantic and contextual representations throughout the detection pipeline, whereas the proposed framework deliberately compresses each session into an explicit structural representation governed by event composition, directed regime change, weighted geometry, and invariant consistency. The observed gaps therefore characterise the predictive component of the difference between high-capacity latent representation learning and constrained structural modelling.

The comparison with LogGPT is particularly informative because model recency alone does not determine the ranking. LogGPT primarily identifies departures from learned next-event regularity, whereas the proposed framework directly models relationships among complete execution regimes. Its F1 of 0.921 ± 0.015 versus 0.904 ± 0.031 for LogGPT indicates that explicit session-level structural modelling can recover anomaly-relevant information without a large pretrained semantic backbone. Conversely, the higher performance of LogLLM and Llama3-ReFT indicates that lexical, semantic, and long-range contextual information provides additional discriminative capacity under a labelled session-classification protocol. The F1 gaps of 0.074 and 0.065 delimit the predictive cost of the proposed method’s more constrained representation on this benchmark; they do not quantify interpretability, computational cost, robustness, or deployment suitability, which are separate properties.

The higher performance of the proposed method in Table 4 relative to Table 2 reflects different supervision and calibration protocols. Table 2 reports HDFS test F1 values of 0.731 and 0.773 with 1% and 5% of development labels disclosed for representation and cross-fitted decision calibration. Table 4 uses complete development labels and a separate validation partition for threshold selection. Both protocols exclude test labels from construction and calibration. Its reported F1 of 0.921 ± 0.015 therefore concerns the more extensively supervised setting within the same execution-regime framework. Table 2 evaluates performance under label scarcity, whereas Table 4 compares discriminative performance with recent neural detectors under the fully labelled development protocol.

Taken together, the additional comparison narrows rather than overextends the empirical claim. The proposed framework does not universally outperform contemporary foundation-model detectors; instead, it occupies an intermediate predictive position while preserving an explicitly defined regime space in which structural changes, distances, neighbourhood relations, and invariant inconsistencies remain directly represented. The experiment therefore identifies not only the magnitude of the predictive differences but also the representational assumption associated with them.

A component ablation evaluates invariant regularisation, geometric regularisation, and learned metric weights under a common base representation. Within each development fitting partition, descriptor mappings, preprocessing statistics, and descriptor-aggregation coefficients are estimated in the common feature-construction stage before metric calibration. These quantities are then shared by the full and ablated variants. The experiment therefore isolates components of metric calibration rather than replacing the underlying telemetry descriptors.

Three interventions are applied separately. The variant without $R_{\text{inv}}$ sets $\lambda_{\text{2}}=0$. The variant without $R_{\text{geom}}$ sets $\lambda_{\text{1}}=0$. The uniform-weight variant fixes $w_{i}=1/p$ and removes projected coordinate search for w. The last intervention concerns the metric-weight vector. It does not change the descriptor-aggregation specification or the fixed weights of invariant families and quality components.

All variants use the same development and test manifests, disclosed weak-label subsets, cross-fitting folds, and 20 initialisation seeds as the full-model experiment. In the shared-configuration comparison, each ablated run uses the discrete configuration selected by its paired full-model run, except for the coefficient explicitly removed. Remaining trainable metric weights are recalibrated from uniform initialisation. Cluster prototypes are refitted, and decision thresholds are recalibrated using development predictions. For uniform w, prototypes and thresholds are refitted with the metric weights fixed. Full-model prototypes, thresholds, and test predictions are not transferred.

In the retuned comparison, each variant repeats development selection over its remaining operative parameters. Without $R_{\text{inv}}$, the original grids are searched for k and $\lambda_{\text{1}}$. Without $R_{\text{geom}}$, they are searched for k and $\lambda_{\text{2}}$. For uniform w, development selection determines k. Regularisation coefficients are not tuning dimensions in this variant because, once the base representation and w are fixed, they cannot change the spherical k-means fit or its predictions. All selection and threshold calibration exclude test labels.

Table 5 reports mean test F1 and sample standard deviation across the 20 initialisations. Differences from the full model are calculated from paired run-level F1 values. The reported dispersion describes sensitivity of the complete calibration procedure to its initialisation, including downstream changes in selected configurations, weights, prototypes, and thresholds. It is conditional on the fixed split and disclosed label subset. These across-initialisation summaries differ from the observed test metrics of the reference fits in Fig 4 and Table 2. The paired-bootstrap intervals in Table 2 instead estimate test-sample uncertainty while keeping the fitted models and thresholds fixed.

Removing invariant regularisation reduces mean F1 by 0.028–0.050 under the shared configurations. Retuning recovers 0.004–0.009, leaving losses of 0.024–0.041. These residual losses indicate that the remaining geometric criterion and cluster-count selection do not fully replace the calibration information supplied by nominal-reference constraints. The larger losses at 1% supervision are consistent with these constraints supplementing sparse disclosed labels. For HDFS, the constraints collectively address template occurrence, frequency, and joint composition. The experiment does not separate the contributions of these three invariant families.

Removing geometric regularisation reduces mean F1 by 0.012–0.021 under shared configurations. The corresponding standard deviations increase from 0.010 to 0.019 and from 0.008 to 0.013 on AIOps, and from 0.015 to 0.026 and from 0.011 to 0.018 on HDFS. This pattern supports a stabilising role within the complete calibration procedure. It does not attribute the increased dispersion exclusively to cluster assignment because configuration selection and threshold calibration also respond to initialisation.

Retuning the variant without geometric regularisation improves mean test F1 by 0.004 on AIOps at 5% supervision and by 0.009 on HDFS at 1%. At the other two settings, test F1 decreases by 0.001. The remaining mean losses relative to the full model are 0.008–0.017. Thus, development-based retuning does not uniformly improve held-out performance or reduce initialisation dispersion. Its mixed effect nevertheless leaves the full model with the highest mean F1 in all four settings.

Uniform metric weights produce the largest mean losses. These are 0.035–0.066 under shared configurations and 0.030–0.057 after cluster-count selection. Equal weighting prevents the metric from adapting to differences in descriptor relevance and redundancy, which is particularly consequential for sparse HDFS template changes. Fixing w also removes the channel through which the regularisation terms influence metric calibration. The resulting loss therefore concerns learned metric adaptation within the integrated model rather than an independent additive contribution of weighting.

Across the evaluated settings, the full model combines the highest mean F1 with lower initialisation dispersion than the ablated variants. The results support complementary roles for nominal-reference constraints, geometric regularisation, and learned metric adaptation under the common base representation. Retuning compensates for part of the detection loss in several settings but does not recover full-model mean performance. These are numerical comparisons for the specified split and weak-label subsets.

## 4 Conclusions

The relevance of this study follows from the operational reality of cloud-hosted, distributed platforms, where tightly coupled components and continuous change produce persistent degradations that are difficult to localise, while telemetry is heterogeneous and labels are scarce [1,3,6,7]. Hence, a regime-oriented approach that can identify sustained behavioural departures from logs and KPI streams under limited supervision is needed, as developed in Sects 2–3 [9,19,26,27].

In Sect 2, we introduce a regime-level formalisation of execution behaviour in which admissible states are defined through invariants and are observed only indirectly via an observation space that separates log events from metric signals. The framework then constructs an empirical regime representation together with a weighted pseudometric structure, and embeds clustering into the optimisation of a hierarchical quality functional that couples geometric regularisation with invariant consistency. This yields a regime space in which regime boundaries and regime transitions are modelled as structural changes in geometry, rather than treated as isolated event anomalies.

The experiments establish separate log-based and metric-based implementations of this formalisation. Joint evaluation remains future work and requires synchronized telemetry from the same system, with both modalities assigned to common execution entities and temporal supports. Log-only, metric-only, and combined representations will be compared using identical regime labels, data splits, weak-label subsets, and threshold-selection rules. Model parameters will be calibrated separately for each configuration using development data. This comparison will assess whether the combined representation improves detection relative to the stronger single-modality configuration.

The experimental results in Sect 3 support execution-regime detection under limited supervision in the separately evaluated KPI and log settings. The component ablation in Table 5 links detection performance to the mechanisms used to calibrate metric geometry under a common base representation. After development-based retuning, removal of invariant regularisation reduces mean F1 by 0.024–0.041. Removal of geometric regularisation produces mean losses of 0.008–0.017 and greater sensitivity of the complete calibration procedure to initialisation. Replacing learned metric weights with uniform weights reduces mean F1 by 0.030–0.057. The full model retains the highest mean F1 in every evaluated setting. These results support the combined use of nominal-reference constraints, geometric regularisation, and learned metric adaptation within the proposed framework.

From a practical perspective, the approach provides an operationally usable way to detect persistent behavioural departures as regime shifts using telemetry that is routinely available in production, including KPI streams and large-scale logs. The weak-supervision results demonstrate detection with restricted development-label availability, while explicitly separating annotation budgets from test prevalence, calibrated decision thresholds, and observed alert fractions. The resulting regime structure can support monitoring and post-incident triage, because it exposes stable regimes and transitional zones explicitly, rather than returning only isolated anomaly flags.

The principal limitations of the present study arise from the static nature of its offline formulation. The regime geometry, cluster configuration, feature weights, and invariant-guided regularisation are calibrated from a fixed observation set, and the current formulation does not define incremental updates of k, w, cluster representatives, or neighbourhood relations as new telemetry arrives. Consequently, online adaptation cannot be inferred from the reported offline results.

Security-oriented evaluation constitutes a separate direction for future work. Malicious activity and benign operational disruptions can produce similar departures from nominal telemetry, so successful operational anomaly detection alone does not establish security-specific discrimination. A security evaluation would require independently verified attack labels together with benign faults, workload changes, and configuration-related disruptions. It should assess whether the regime representation distinguishes attack-related behaviour from both nominal execution and benign operational anomalies. False security alerts on benign disruptions should be reported at a decision threshold fixed independently of the test labels. This evaluation would determine whether invariant-guided regime geometry provides discriminative information beyond general operational anomaly detection.

Concept drift introduces a related limitation. Persistent changes in workload, software configuration, deployment state, or nominal execution behaviour may alter both the distribution of regime features and the empirical validity of previously calibrated invariant relationships. Without an explicit drift-detection and recalibration mechanism, such changes may be represented as anomalous structural transitions even when they correspond to a new nominal operating regime, or may progressively reduce the separation between nominal and anomalous regimes.

Streaming operation would therefore require mechanisms that are outside the present formulation: online construction of regime transitions from incoming telemetry, bounded-state or incremental computation of feature and invariant statistics, dynamic maintenance of neighbourhood relations and cluster representatives, and a criterion for distinguishing transient anomalies from persistent distributional drift before recalibration. This distinction is essential because indiscriminate adaptation to recent observations could incorporate anomalous behaviour into the nominal regime geometry.

These limitations also delimit the present real-time deployment claim. The current framework does not define incremental optimisation of functional (11) or real-time maintenance of regime geometry under continuous telemetry ingestion. It should therefore be regarded as complementary to existing production observability and operational-management infrastructures rather than as a replacement for telemetry acquisition, orchestration, scheduling, infrastructure management, or incident-response automation. Extending the framework towards streaming operation requires jointly addressing incremental geometry, drift detection, controlled recalibration, and computational latency.

The present experiments were designed to validate the analytical framework in an offline setting rather than to benchmark a production implementation. Consequently, wall-clock latency, peak memory consumption, throughput, and telemetry-ingestion overhead were not measured under a live cloud-native workload, and the present results should not be interpreted as demonstrating real-time production scalability. The complexity analysis above instead identifies exhaustive pairwise geometry as the principal worst-case scaling constraint and indicates where sampled, indexed, approximate, or incremental computations would be required in a production-oriented implementation. Application to production telemetry requires privacy-aware preprocessing because logs can contain personal identifiers, credentials, or confidential application values. Data minimisation and appropriate redaction should precede feature construction and sharing. Parsed templates and aggregated descriptors should also be checked for residual sensitive information because aggregation alone does not guarantee anonymisation.

Further research should be directed towards extending the model to online scenarios by developing incremental updating procedures for weighted pseudometrics and invariant-violation functionals, adaptive maintenance of execution-regime geometry under concept drift, and bounded-memory optimisation capable of supporting low-latency processing of continuously evolving telemetry streams. Additional research will also consider integrating the temporal evolution of transitions $\Delta_{r}$, evaluating sliding-window updates of regime prototypes, and conducting cross-environment validation on independently collected telemetry from heterogeneous industrial architectures and production observability stacks. Such validation is required to determine whether the regime geometry and invariant relationships remain stable under changes in system architecture, workload, instrumentation, and organisational operating conditions.
