# Supplementary material for: An invariant-guided model for automated detection of anomalous execution regimes from production telemetry
Source: PLoS One. 2026 Nov 12;21(11):e0362583. doi: 10.1371/journal.pone.0362583 (PMC13759071; doi:10.1371/journal.pone.0362583)
Supplement: Supplementary file 1 [file pone.0362583.s002.pdf]

RESPONSE TO COMMENTS

Response to the Academic Editor

We sincerely thank the Academic Editor for the careful evaluation of our manuscript and for the detailed and constructive comments. We greatly appreciate the time and effort devoted to assessing our work. The comments have been invaluable in improving the scientific quality, methodological clarity, and overall presentation of the manuscript.

In preparing the revised version, we carefully addressed every comment and substantially revised the manuscript accordingly. The revision extends well beyond editorial corrections and includes significant improvements to the conceptual positioning of the study, methodological transparency, experimental validation, statistical analysis, and academic presentation. In particular, we revised the title, Abstract, Introduction, Sections 2.1 and 2.2, Section 3, and the Conclusions; clarified the operational implementation of the proposed mathematical framework; strengthened the experimental validation through additional statistical analysis; refined the positioning of the proposed framework with respect to modern cloud observability infrastructures; and comprehensively revised the manuscript to ensure a consistent formal academic style throughout.

Comment 1

The paper is written in an unacceptably informal and conversational register, falls well below the standard of academic English…

This is not written to the standards expected at PLOS One. Kindly revise the paper with proper use of language as expected from a scientific paper. Use of terms like "They're", "we're" and so on are not befitting of scientific writing at least not in PLOS One.

Response

We thank the reviewer for this important comment. We agree that the original version contained conversational language and informal stylistic constructions that were not appropriate for a scientific manuscript. In response, we performed a comprehensive linguistic revision of the entire paper to ensure a consistent academic register throughout.

The revision extended well beyond grammatical editing. The title, Abstract, Introduction, Sections 2.1 and 2.2, Section 3, and the Conclusions were substantially rewritten to achieve a precise and formal scientific style. Informal contractions and conversational expressions (e.g., "they're", "we're", "fall apart", "all over the place", "figure out", "weird", and similar wording) were removed throughout the manuscript and replaced by precise scientific terminology.

The revision also included a systematic harmonisation of terminology across the manuscript. Concepts are now referred to consistently using formal expressions such as execution regimes, anomalous execution regimes, production telemetry, invariant-guided representation, weighted pseudometric, geometric regularisation,

and weak supervision, thereby eliminating stylistic and terminological inconsistencies present in the original version.
In addition, the Abstract was completely rewritten to follow a standard scientific structure, clearly presenting the research motivation, methodological contribution, experimental validation, and principal findings in formal academic English. The Introduction was substantially revised to provide a more rigorous and technically accurate description of cloud-native production environments, observability infrastructures, execution regimes, and the scope of the proposed framework. The methodological and experimental sections were likewise edited to ensure consistent terminology, precise definitions, and an objective scientific style, while the Conclusions were revised to present the limitations and practical implications using formal academic language.
Overall, the manuscript has been thoroughly revised to replace conversational narrative with precise scientific exposition. We believe that the revised version is now consistent with the academic writing standards expected for publication in PLOS ONE.

Comment 2
The paper … devotes excessive space to elementary material while leaving its core formal constructs -- including the local feature functions l_i and m_j, the weighting functions α_k and β_k, and the invariant violation functionals ψ_m -- entirely unspecified in operational terms, rendering the experiments irreproducible and the claimed contributions unverifiable.

Response
We sincerely appreciate this important observation. We agree that, although the mathematical framework presented in the original manuscript was complete at the formal level, the operational interpretation of several mathematical constructs was described too implicitly. As a consequence, the correspondence between the mathematical model introduced in Section 2 and its implementation within the experimental pipeline was insufficiently explicit.
To address this concern, we substantially revised Section 3 by introducing a new subsection that provides a complete operational instantiation of the proposed mathematical framework. Rather than modifying the mathematical model itself, we explicitly specify how each formal construct is realised during experimental evaluation for every benchmark dataset.
Specifically, the revised manuscript now establishes an explicit mapping between the mathematical entities defined in Equations (1)–(11) and their operational implementation. The construction of elementary observations, execution transitions Δ_r^L and Δ_r^M, local feature functions l_i and m_j, weighting functions α_k, β_k, and w_i, invariant violation functionals ψ_m, invariant weights η_m, geometric regularisation coefficients ρ_j, regularisation parameters λ_1 and λ_2, neighbourhood parameter k_{nn}, pseudometric parameter q, clustering parameter k, and the hierarchical quality functional are now described through explicit operational rules.

In particular, the revised Section 3 now reports the adopted operational configuration: $q = 2$, $k_{nn} = 10$, $\rho_1 = \rho_2 = \rho_3 = 1/3$, $\eta_1 = \eta_2 = \eta_3 = 1/3$, $\gamma_1 = \gamma_2 = \gamma_3 = 1/3$, $k \in \{2, \ldots, 12\}$, and $\lambda_1, \lambda_2 \in \{0, 0.05, 0.10, 0.20, 0.50\}$, together with the weak-supervision levels of 5% and 1%.

The revised manuscript also specifies how these constructs are instantiated for each benchmark individually. For the HDFS benchmark, the framework is instantiated through the log component because synchronised KPI streams are unavailable. Conversely, for the 2018 AIOps Challenge KPI benchmark, only the metric component is instantiated because aligned execution logs are not provided. This distinction eliminates ambiguity regarding the relationship between the abstract hybrid formulation and the dataset-specific experimental realisations.

Importantly, these revisions do not alter the proposed mathematical framework, optimisation procedure, theoretical analysis, or experimental results. Their purpose is to make explicit the implementation-level information that was previously implicit. Consequently, every principal mathematical construct participating in the experimental pipeline is now associated with an explicit operational definition, parameterisation rule, data source, and dataset-specific implementation. We believe that these revisions substantially improve the transparency, reproducibility, and verifiability of the proposed methodology while preserving its original scientific contribution.

Comment 3

The experimental comparisons are restricted to outdated baselines, evaluated on aging and unrepresentative datasets under an offline protocol that is inconsistent with the production monitoring use case the paper purports to address.

Response

We appreciate this comment. We agree that the original manuscript did not sufficiently explain the methodological rationale behind the choice of baselines, datasets, and evaluation protocol. The revised manuscript therefore clarifies that the objective of the study is not to provide a production leaderboard over heterogeneous anomaly-detection architectures, but to formulate and reproducibly validate an interpretable mathematical framework for execution-regime modelling.

To address the concern about baselines, Section 1.2 has been substantially rewritten as a paradigmatic review of contemporary approaches, including rule-based, statistical, sequential log-based, transformer/language-model, graph-based observability, KPI-oriented AutoML, and root-cause-analysis methods. Table 1 has also been revised to compare these paradigms according to the mathematical object they model. This revision clarifies that the proposed approach addresses a different scientific question from black-box representation-learning detectors: it explicitly models execution regimes using feature mappings, weighted pseudometrics, invariant functionals, geometric regularisation, and optimisation criteria. Accordingly, DeepLog, PLELog, and ADS are retained as experimental baselines because they are the closest methodological analogues under comparable

supervision assumptions: sequential log modelling, semi-supervised log anomaly detection, and KPI-oriented detection. The purpose of the comparison is therefore not to rank incompatible deep-learning architectures, but to evaluate alternative formulations of anomaly representation under limited supervision.

The dataset rationale has also been clarified in Section 3. The selected datasets are large-scale, real-world, publicly available benchmarks used for reproducible evaluation in AIOps and log-analysis research, where proprietary industrial telemetry is rarely accessible for independent verification. The AIOps Challenge dataset provides 29 labelled KPI streams from large Internet-company systems, while the HDFS benchmark contains approximately 11.2 million log records organised into about 575 thousand labelled execution sessions. These datasets cover the two principal observability modalities formalised in the model: continuous KPI dynamics and discrete log-event structure. We also clarified that HDFS remains part of the LogHub/LogHub-2.0 benchmark ecosystem and is therefore used not merely as a historical dataset, but as a public reference benchmark for reproducible log-analysis evaluation.

Finally, the revised manuscript explicitly distinguishes mathematical validation from engineering deployment. The adopted offline protocol is now presented as a deliberate validation stage for the proposed mathematical apparatus rather than as a claim of complete production-monitoring implementation. Its purpose is to isolate whether the proposed feature mappings, weighted pseudometric, invariant regularisation, and optimisation criterion can construct a stable and interpretable execution-regime space under reproducible conditions. A complete online production system would additionally require streaming ingestion, incremental feature maintenance, concept-drift adaptation, latency control, bounded-memory processing, distributed state synchronisation, and online recalibration. These mechanisms are important for deployment, but they concern the engineering architecture rather than the mathematical formulation evaluated in the present paper.

Overall, the revised manuscript now explicitly delimits the scope of the contribution: it proposes and validates an interpretable mathematical model of anomalous execution regimes, not a complete production observability platform. This clarification, together with the updated literature review, dataset justification, and offline-protocol explanation, directly addresses the concern raised in this comment.

Comment 4

… the reported performance improvements are modest and unsupported by any test of statistical significance.

Response

We thank the reviewer for raising this important issue. We agree that the original manuscript relied primarily on point estimates of Precision, Recall, and F1 score and therefore did not provide sufficient statistical evidence to support the reported performance improvements. To address this concern, we substantially revised

Section 3 by introducing a dedicated statistical validation subsection centred on a newly added Table 2, which extends the comparative analysis originally presented in Figure 4 with formal statistical inference, uncertainty quantification, and effect-size analysis.

Table 2 constitutes the central element of this revision. It summarises the complete statistical validation of the comparative experiments and is accompanied by a detailed methodological description and an expanded interpretation of the obtained results. The revised manuscript now evaluates the robustness of the reported performance by means of 1000 stratified bootstrap resamples, where an execution regime is treated as the elementary resampling unit after completion of the feature-construction, invariant-mapping, weighting, and clustering procedures defined in Section 2.

To provide a comprehensive assessment of model stability, two complementary bootstrap protocols were introduced. The fixed-geometry protocol evaluates the stability of the final decision layer under a fixed execution-regime representation, whereas the full-pipeline protocol additionally reconstructs the complete invariant-guided execution-regime space for every bootstrap sample, including feature-space construction, weighted pseudometric estimation, invariant regularisation, clustering, and anomaly-score computation. Consequently, the statistical validation assesses not only the stability of the final anomaly decision but also the robustness of the invariant-guided execution-regime representation, weighted pseudometric, and geometric regularisation introduced in Section 2.

Accordingly, Table 2 reports 95% bootstrap confidence intervals, Holm-adjusted paired Wilcoxon signed-rank tests, Cliff's δ effect-size estimates, and both absolute and relative performance improvements for every experimental setting. The full-pipeline improvements range from 0.041 to 0.105 F1, with a median improvement of 0.063 F1 and a median relative improvement of 9.2%. After Holm correction, all pairwise comparisons remain statistically significant (adjusted $p = 0.006–0.043$), while Cliff's δ ranges from 0.37 to 0.64, corresponding to small-to-medium through large practical effects.

The revision also substantially expands the interpretation of these statistical results. Rather than treating statistical significance as an isolated property of the reported metrics, the revised discussion explains why the magnitude of improvement varies across datasets in terms of the proposed mathematical framework. The largest improvements are observed for KPI telemetry under 1% supervision, where invariant-guided geometric regularisation compensates for the scarcity of labelled anomalies by preserving structural relationships among execution regimes before the final anomaly decision is made. Conversely, the smaller improvements observed for HDFS under 5% supervision are consistent with the characteristics of that benchmark, where specialised sequential models such as DeepLog already exploit highly regular local event dependencies. Thus, the observed variability follows the expected interaction between telemetry modality, supervision level, and the mathematical assumptions underlying the competing approaches rather than reflecting inconsistent behaviour of the proposed method.

Importantly, the additional statistical analysis demonstrates that the observed gains originate primarily from the invariant-oriented execution-regime representation rather than from favourable sampling or threshold selection. The small difference between the fixed-geometry and full-pipeline bootstrap protocols indicates that repeated reconstruction of the weighted pseudometric, invariant functionals, and regime geometry introduces only limited additional variability, providing independent evidence for the robustness of the proposed mathematical framework.
Finally, we also addressed the reviewer's concern regarding the practical importance of the reported improvements. Although the absolute F1 gains range from 0.041 to 0.105, they were obtained under a particularly challenging weak-supervision setting (1–5% labelled anomalous regimes) and against strong specialised baselines for KPI-oriented and sequential log-based anomaly detection. Under these conditions, statistically supported relative improvements of 5.7–16.3%, accompanied by small-to-large effect sizes, represent meaningful practical advances rather than marginal numerical fluctuations. Moreover, these improvements are achieved while simultaneously preserving explicit execution-regime semantics, invariant consistency, interpretability, and the deterministic geometric structure of the proposed model.
Taken together, the new Table 2, the accompanying methodological description, and the expanded interpretation of the experimental results directly address both aspects of the reviewer's comment. The revised manuscript now complements the original point estimates with formal statistical validation, uncertainty quantification, effect-size analysis, and a detailed explanation of the practical significance of the observed improvements, thereby providing substantially stronger experimental evidence for the proposed invariant-oriented framework.

Comment 5
Most fundamentally, the paper misrepresents the state of cloud computing practice, ignores the sophisticated observability, scheduling, and DCIM tooling already deployed in production environments, and conflates performance degradation with security incidents in a manner that renders its central security claims technically incohere.

Response
We thank the reviewer for raising this important concern. We agree that the original version did not sufficiently distinguish the proposed mathematical framework from the mature observability, orchestration, scheduling, autoscaling, and infrastructure-management tooling already deployed in modern cloud-native environments. We have therefore revised the manuscript to clarify the scope of the proposed approach and to avoid any implication that it replaces existing production systems.
First, Section 1.1 has been revised to explicitly acknowledge the current state of cloud computing practice. The revised manuscript now states that modern cloud-native production environments already rely on mature observability, orchestration, scheduling, autoscaling, and infrastructure-management tooling, and

that such systems support telemetry acquisition, aggregation, visualisation, alerting, resource control, and operational automation. The same paragraph clarifies that the proposed framework should be interpreted as “an invariant-guided analytical layer above existing observability and operational-management stacks rather than as an alternative monitoring, scheduling, or DCIM platform.”

Second, Section 1.2 now further specifies the relationship between the proposed framework and existing production tooling. The revised text explains that observability platforms, schedulers, autoscaling mechanisms, DCIM systems, SIEM/SOAR tools, and AIOps pipelines solve operational tasks such as telemetry collection, infrastructure control, alert routing, visualisation, incident management, and remediation support. The proposed model does not duplicate these functions; instead, it focuses on the formal representation, invariant-guided organisation, and geometric analysis of execution regimes derived from telemetry already collected by such systems.

Third, Section 2.1 has been revised to remove the ambiguity concerning the relationship between performance degradation and security incidents. The formal definition now treats security relevance as a possible application context rather than as an automatic consequence of performance degradation. In particular, the revised manuscript states that anomalous execution regimes are deviations from nominal execution invariants and that “such deviations are not interpreted as security incidents per se and do not constitute sufficient evidence of malicious activity.” It further clarifies that these deviations represent operational evidence that may support reliability assessment, operational diagnosis, or security investigation depending on context.

Finally, the Conclusions have been extended to make the limitation explicit. The revised manuscript now states that the proposed framework complements existing production observability and operational-management infrastructures by providing an invariant-guided analytical representation of execution regimes derived from their telemetry, rather than replacing functions such as telemetry acquisition, orchestration, scheduling, infrastructure management, or incident-response automation.

Taken together, these changes reposition the contribution of the paper more precisely. The revised manuscript now avoids presenting the proposed framework as a substitute for modern cloud observability, scheduling, DCIM, SIEM/SOAR, or AIOps tooling. Instead, it defines the contribution as a complementary mathematical analytical layer operating on telemetry produced by such infrastructures. The security-related claims have also been reformulated so that anomalous execution regimes are not equated with security incidents, while their potential security relevance is retained only as a context-dependent interpretation requiring further analysis. We believe that these revisions directly address the reviewer’s concern by clarifying both the technological scope of the proposed framework and the status of security relevance in the formal model.

Response to Reviewer #1

We sincerely thank the Reviewer for the careful evaluation of our manuscript and for the positive overall assessment of our work. We are especially grateful for the constructive suggestion regarding the future development of the proposed framework. We appreciate the reviewer's recognition that the principal limitation of the current study is already transparently acknowledged and that the suggested improvement concerns further strengthening the discussion rather than the scientific validity of the proposed methodology.
In response to this valuable recommendation, we have expanded the discussion of limitations and future research directions in the Conclusions by explicitly outlining the principal computational challenges associated with extending the proposed mathematical framework to online, real-time streaming environments.

Comment 1
The primary limitation of your study—that it currently relies on an offline, static analysis protocol without streaming data updates—is transparently acknowledged in your conclusion. To strengthen the paper further, you might briefly expand in your discussion on the specific computational challenges that will need to be overcome to adapt this weighted pseudometric and clustering optimization into an online, real-time streaming environment in your future work.

Response
We sincerely thank the reviewer for this valuable suggestion. We agree that a more explicit discussion of the computational challenges associated with extending the proposed framework to online deployment improves the completeness of the manuscript.
Accordingly, we have expanded the limitations and future-work discussion in the Conclusions. The revised text now outlines the principal algorithmic challenges that must be addressed for future online implementations, including incremental model updating, adaptation to concept drift, bounded-memory optimisation, and low-latency processing of continuously evolving telemetry streams.
We appreciate this constructive recommendation, which has strengthened the discussion of future research directions and the practical evolution of the proposed framework.
