# Supplementary material for: An invariant-guided model for automated detection of anomalous execution regimes from production telemetry
Source: PLoS One. 2026 Nov 12;21(11):e0362583. doi: 10.1371/journal.pone.0362583 (PMC13759071; doi:10.1371/journal.pone.0362583)
Supplement: Supplementary file 2 [file pone.0362583.s004.pdf]

RESPONSES

Response to the Academic Editor

Dear Dr Atoum,

We thank you for your assessment and for identifying consistency of the reported results and experimental reproducibility as priorities for this revision. We have addressed these concerns through corrections to the figures and tables, explicit specification of the evaluation protocol, and public archiving of a reference implementation.

"Kindly review and address all comments related to inconsistencies in the figures and tables, as well as concerns regarding the reproducibility of the experiments."

Response: Table 2 and Figure 4 now use the same reference-fit predictions and explicitly defined test sets. Section 3 reports their sizes, class compositions, anomaly rates, and confusion matrices, and distinguishes label-disclosure budgets from decision thresholds. Figure 4 has been regenerated as dataset-specific panels, and its interpretation now agrees with the tabulated results. Figures 1–3 specify their diagnostic samples and construction procedures. Figure 3 has been recalculated from the original weighted regime vectors, with symmetry and a zero diagonal verified within numerical tolerance. The accompanying quantitative interpretations are supported by explicit calculations. These changes are detailed in our responses to Reviewer #4, comments 1–3 and 9.

The revised experimental specification provides invariant formulas, nominal-reference estimation rules, selected hyperparameters, seeds, baseline configurations, and calibration procedures. Component-ablation results are included in Table 4, while Table 2.1 reports complementary detection and ranking metrics. Paired moving-block bootstrap intervals on F1 differences replace the previous significance testing. The manuscript distinguishes test-sample uncertainty from variation caused by initialisation and weak-label selection. These revisions are documented in our responses to comments 3 and 5–8.

A Python reference implementation is publicly archived on Zenodo at https://doi.org/10.5281/zenodo.23218142 under the GNU General Public License, version 3 or later. The package includes installation instructions, runnable examples, and 50 automated tests. The public dataset sources, feature-construction rules, partitioning procedures, and experimental settings are identified in the manuscript. Together, these materials provide an executable basis for inspecting the proposed method and conducting independent evaluations. Our responses to comments 11–12 explain the implementation and execution environment.

If feasible within the journal's review process, we would be grateful for a timely assessment of this revision. The study supports one of the original research contributions in Frederik Huranyk's PhD dissertation, which is scheduled for preliminary defence later this month. We mention this circumstance to explain the timing of our request and fully respect the time required for a thorough evaluation.

Responses to the Journal Requirements:

We have corrected citation mismatches and revised the reference list to support the statements and methods for which the sources are cited. DeepLog, PLELog, and ADS now refer to [21], [22], and [36], respectively. References [9], [15], and [16] have been replaced with relevant publications on automated log analysis, anomaly detection, and deep learning for anomaly detection.

The missing LogLLM and LogADReFT references have been added as [41] and [42]. The experimental description distinguishes the LogADReFT method from the evaluated Llama3-ReFT configuration. After assessing its methodological relevance, we have also added the invariant-mining study by Lou et al. as [40] and discussed its relationship to the proposed framework.

These changes supply the missing methodological sources and align the citations with the revised text. They are documented in our response to Reviewer #4, main comment 8 and minor comments 1–3.

Responses to Reviewer #4

We thank Dr Tetiana Afanasieva for her careful assessment of the revised manuscript and for the precise, technically substantive comments. Her scrutiny of the evaluation protocol, statistical reporting, and diagnostic figures helped us strengthen the consistency and traceability of the results. We also appreciate her recognition of the improvements already made. Below, we address each comment and question and explain the corresponding revisions.

If feasible within the journal's review process, we would be grateful for a timely assessment of this revision. The study supports one of the original research contributions in Frederik Huranyk's PhD dissertation, which is scheduled for preliminary defence later this month. We mention this circumstance solely to explain the timing of our request and fully respect the time required for a thorough evaluation.

1. "Some of the HDFS numbers don't seem to fit the protocol as described. The paper says the top 1% (or 5%) of scores are flagged, and by the authors' own counts about 2.9% of HDFS sessions are anomalous. If only 1% is flagged, recall can't exceed about 0.34, which caps F1 at roughly 0.51. The text reports about 0.73. A similar back-of-the-envelope check on AIOps gives two different implied anomaly rates for the same data, about 5.4% and 1.3%. I suspect the evaluation set or threshold is different from what's written. Please state exactly which set the metrics come from, give its size and anomaly rate, and include confusion matrices. Please also clarify whether "1%" and "5%" mean the share of labelled anomalies, the flagging threshold, or both."

Response: Thank you for identifying the inconsistency between the stated alert quota and the reported detection results. Your calculation is correct: with approximately 2.9% anomalous HDFS sessions, flagging only 1% of the test set limits recall to approximately 0.34 and F1 to approximately 0.51.

We have revised Section 3 to distinguish the annotation budget from the decision rule. The 1% and 5% settings denote the proportions of development anomaly labels disclosed to the pipeline; the same proportions of nominal labels are disclosed. They do not specify the fraction of test regimes flagged as anomalous. Threshold calibration uses five-fold out-of-fold predictions within this same annotation budget, without an additional fully labelled validation set. Fold-specific scores are mapped to nominal-reference percentiles, and the selected threshold is fixed before test evaluation.

The HDFS test set contains 115,013 sessions, including 3368 anomalies, with a prevalence of 2.928%. The AIOps test set contains 20,000 regimes, including 1067 anomalies, with a prevalence of 5.335%. Each dataset uses identical test membership and prevalence at both supervision levels. We have specified the partitioning, window-labelling, annotation-count, and calibration procedures.

We have also added confusion matrices for the reference initialisation and distinguished these fitted-model counts from the repeated-initialisation and bootstrap estimates. At 1% supervision, HDFS has TP = 2141, FP = 349, FN = 1227, and TN = 111,296, yielding Precision = 0.860, Recall = 0.636, and F1 = 0.731. The observed alert fraction is 2.165%, rather than 1%. At 5% supervision it is 2.673%. The corresponding AIOps alert fractions are 4.035% and 5.000%; its prevalence remains 5.335% in both settings.

These explicit counts reconcile the reported precision, recall, and F1 without requiring different evaluation prevalences. We have removed the conflicting upper-tail descriptions and revised the related interpretations and comparison with Table 3. The central claim remains detection under limited development supervision, now supported by an explicit separation of annotation availability, decision calibration, and independent test evaluation.

2. "Figure 4 doesn't match the text or Table 2:

- In Fig. 4b, precision goes up as supervision increases. The text says the opposite.
- The baseline F1 values at 1% in Fig. 4a (about 0.73–0.76) are well above those in Table 2 (0.646 and 0.651).
- It's unclear which dataset is shown. ADS, DeepLog and PLELog share one set of axes even though they apply to different data.
- There is a 2% point that the protocol doesn't mention.

Please regenerate the figure per dataset, from the same runs as Table 2."

Response: Thank you for identifying the inconsistencies between Figure 4, the text, and Table 2.

We have regenerated Figure 4 as four dataset-specific panels: AIOps F1 and precision, and HDFS F1 and precision. ADS appears only for AIOps, while DeepLog and PLELog appear only for HDFS. The unsupported 2% point has been removed; the figure now contains only the evaluated 1% and 5% development-label budgets.

Figure 4 and Table 2 now use the same reference-initialisation predictions, complete test manifests, and regime-level evaluator. Each plotted point is an observed test metric calculated before bootstrap resampling. The F1 values agree

with Table 2 at the reported three-decimal precision, and the proposed-method values are supported by the reported confusion matrices. Table 2 estimates uncertainty in paired F1 differences using 10,000 moving-block bootstrap replicates with the fitted models and thresholds held fixed. Sensitivity to recalibration is reported separately through the initialisation repetitions, weak-label-subset experiment, and component ablation.

The plotted F1 values match Table 2: the proposed method achieves 0.758 and 0.803 on AIOps and 0.731 and 0.773 on HDFS. ADS achieves 0.646 and 0.742; the strongest HDFS baselines are PLELog at 1%, with F1 = 0.651, and DeepLog at 5%, with F1 = 0.724.

We have corrected the precision panels and replaced the previous interpretation. Proposed-method precision decreases from 0.880 to 0.830 on AIOps and from 0.860 to 0.810 on HDFS as supervision increases, while recall and F1 increase. The revised analysis explains this as increased anomaly coverage accompanied by a larger investigation workload, rather than as a predetermined effect of an alert quota. Dataset-specific differences are discussed in relation to KPI regime descriptors and log-sequence representations.

The methodological details are now provided before the figure, the caption identifies the four panels concisely, and the following analysis explains their operational and representational implications.

3. “Please describe the evaluation set for the weak-supervision experiment. The weak labels enter model selection through the label-consistency term, and the bootstrap is described as resampling "the test execution regimes." However, I couldn't find where that test set is defined, or whether the labelled regimes are excluded from scoring. It would also help to note that the 20 repetitions vary only the k-means initialization. Ideally, add a few runs with different weak-label subsets.”

Response: Thank you for requesting clarification of the evaluation population and the sources of variability.

Section 3 now explicitly distinguishes disclosed development regimes from the independent test set. The HDFS evaluation manifest contains 115,013 sessions, including 3368 anomalies, with a prevalence of 2.928%. The AIOps manifest contains 20,000 regimes, including 1067 anomalies, with a prevalence of 5.335%. Any construction-related exclusions are applied before the evaluation manifests are fixed. Their membership remains unchanged between supervision settings and repetitions.

Disclosed labels enter the development label-consistency term and cross-fitted decision calibration. They are excluded from test metrics by partition membership. Within each calibration fold, held-out regimes and their labels are excluded from fitting and model selection. Concealed development labels and test labels do not enter model selection or threshold calibration. The fixed-geometry bootstrap resamples only completed test prediction records.

We have clarified that the original 20 repetitions vary only spherical k-means initialisation. Weak-label identities, partitions, cross-fitting folds, and optimisation

settings remain fixed. Variation in fitted weights, selected configurations, and thresholds is a downstream consequence of initialisation.
We have added a separate experiment with five independently sampled weak-label subsets and four common initialisations per subset. Annotation counts are unchanged, and each 1% subset is nested within its corresponding 5% subset. The complete calibration procedure is repeated for every configuration using the same test manifest. Results are averaged within each subset before calculating the mean and sample standard deviation across subsets. This dispersion retains residual initialisation variability and is not presented as a pure label-selection variance component.
The proposed method achieves 0.752 ± 0.018 and 0.799 ± 0.011 on AIOps, and 0.724 ± 0.025 and 0.768 ± 0.016 on HDFS, at 1% and 5% supervision, respectively. The minimum paired advantages over the strongest applicable baseline are 0.079, 0.039, 0.047, and 0.027 in those four settings. The strongest HDFS baseline changes in some subsets, while the proposed method retains an advantage in every tested subset.
These additional results are reported separately from Figure 4 and Table 2. They support robustness across the tested annotation compositions while explicitly acknowledging greater sensitivity at the smaller label budget.

4. “The title and parts of the text claim security relevance that the experiments don't show. Both datasets are labelled for operational problems: failed HDFS blocks and operator-marked KPI anomalies. Section 2.1 itself says invariant violations aren't evidence of a security incident, which I agree with. I'd suggest adjusting the title and phrases such as "fundamentally important for information security tasks" to reflect operational anomaly detection. Alternatively, the authors could clearly mark security application as future work.”
Response: We thank the reviewer for identifying the mismatch between the security framing and the evaluated targets. We agree that anomalous HDFS block sessions and operator-labelled KPI anomalies support an operational anomaly-detection evaluation. Their labels do not establish malicious activity.
We have changed the title to “An Invariant-Guided Model for Automated Detection of Anomalous Execution Regimes from Production Telemetry.” We have revised the Introduction, Section 2.1, and Results and Discussion to align the claims with the operational ground truth. The dataset description now explicitly states that neither benchmark provides attack-specific labels for the present evaluation. The phrase “fundamentally important for information security tasks” has been removed.
The revised manuscript distinguishes detection of departures from nominal execution from attribution of their underlying cause. This distinction defines the scope of the reported experiments without imposing a general limitation on the information that the regime representation might provide in other settings. The retained empirical claim concerns operational anomaly detection under limited supervision using an explicit invariant-guided regime geometry.

We have also identified security-oriented evaluation as future work in the Conclusions. The proposed evaluation includes independently verified attacks and benign operational disruptions. It would assess discrimination between these categories and report false security alerts on benign disruptions at a threshold fixed independently of the test labels. This would establish whether the representation contributes security-specific information beyond general operational anomaly detection.

5. “The invariants need to be specified. I found the names of three invariant families per dataset, but not the functionals φ_m themselves, nor which data the "nominal" reference values are estimated from. Please give the formulas. Please also report the selected k, λ1 and λ2 for each setting. Without them, readers can't tell whether the invariant term was actually active (λ2 > 0).”

Response: We thank the reviewer for requesting explicit invariant functionals, nominal-reference sources, and selected configurations. We have expanded Section 3 to specify all three invariant families for each dataset, retaining the manuscript notation ψ_m.

For HDFS, the functionals evaluate the nominal rarity of template-occurrence changes, marginal departures in relative-frequency changes, and departures from their joint trace composition. For KPI data, they evaluate changes in window level, temporal slope, and variability. Each family has an explicit departure statistic, nominal tolerance, and bounded monotone violation function. The manuscript identifies these constraints as soft nominal consistency relationships rather than hard correctness guarantees.

Nominal references are estimated from disclosed nominal regimes in the permitted development fitting partition. We clarify that these references concern changes associated with nominal-labelled regimes and do not require both constituent regimes to have disclosed nominal labels. Concealed labels are not consulted. Within cross-fitting, held-out observations are excluded from reference estimation. Test observations and labels are excluded throughout fitting and selection. The reference statistics remain fixed during feature-weight calibration.

For KPI series with limited nominal annotations, we use a continuous combination of series-specific and equally weighted pooled development references. This avoids an abrupt fallback at a sample-count boundary and prevents longer series from dominating the pooled reference.

We have also clarified configuration selection. The integrated criterion calibrates the representation for fixed $(k, \lambda_1, \lambda_2)$. Candidate configurations are compared using held-out development predictions. Their penalised training-objective values are not compared across different coefficients. Importantly, $\lambda_2 = 0$ remains an eligible candidate.

The reference configurations are (3,0.05,0.10) and (4,0.05,0.10) for AIOps at 1% and 5% supervision. The corresponding HDFS configurations are (4,0.10,0.20) and (6,0.05,0.20). These configurations are also the modes of the 20-initialisation

experiment, selected in 17, 18, 16, and 17 repetitions, respectively. The fully labelled HDFS comparison in Table 3 uses (6,0.05,0.10).
All reported reference configurations therefore have $\lambda_2 > 0$. Positive fitting-regime violation values also give a non-zero invariant penalty, confirming that the term participates in representation calibration. We distinguish this evidence of an active term from evidence of its predictive contribution, which is assessed by ablation.

6. "The Introduction and Conclusions both refer to an ablation study, including its outcome, but I couldn't find it in the manuscript. Please include those results. Removing R_inv, R_geom and the learned weights one at a time would be the most informative."
Response: We thank the reviewer for identifying the missing ablation results. We have added the component-ablation experiment in Section 3, reported its results in Table 4, and revised the corresponding statements in the Introduction and Conclusions.
The experiment separately removes R_{inv}, removes R_{geom}, and replaces learned metric weights with $w_i = 1/p$. All variants share the same development-derived base descriptors and aggregation specification. They also use identical development and test manifests, disclosed weak-label subsets, cross-fitting folds, and 20 paired initialisation seeds. Remaining trainable metric weights, cluster prototypes, and development-calibrated thresholds are refitted.
Table 4 includes a shared-configuration comparison and a development-retuned comparison. The first retains the discrete configuration selected by the paired full-model run, apart from the removed coefficient. The second reselects the remaining operative parameters without using test labels. This tests both component removal under a controlled configuration and the extent to which its loss can be compensated by retuning.
After retuning, removing invariant regularisation reduces mean test F1 by 0.024–0.041. Removing geometric regularisation reduces it by 0.008–0.017 and increases the sensitivity of the complete calibration procedure to initialisation. Uniform metric weights reduce mean F1 by 0.030–0.057. Retuning has mixed effects on test performance, and the full model retains the highest mean F1 in every evaluated setting.
We clarify that the weight ablation concerns metric calibration within the integrated framework. Fixing w also removes the mechanism through which the regularisation terms adapt metric geometry. Its contribution is therefore not interpreted as additive and independent of regularisation. Likewise, the invariant ablation evaluates the three invariant families collectively.
The revised Conclusions refer explicitly to Table 4 and distinguish mean detection performance from initialisation dispersion. The earlier statement about a measured deterioration in regime separability has been replaced by conclusions supported by the reported ablation metrics.

7. “The significance testing should be revised. A Wilcoxon test on bootstrap replicates doesn't give a meaningful p-value, because the replicates aren't independent. In Table 2, non-overlapping AIOps intervals sit next to Cliff's δ values of 0.64 and 0.50, which doesn't seem consistent. Reporting a paired bootstrap interval on the F1 difference, or McNemar's test on per-regime decisions, would be more appropriate.”
Response: We thank the reviewer for identifying the inappropriate significance procedure. We have removed the Wilcoxon-derived p-values, Holm-adjusted p-value row, Cliff’s δ, and qualitative effect-size categories. The revised analysis directly estimates uncertainty in the paired test F1 difference.
Each bootstrap replicate uses identical test-regime identifiers and multiplicities for both methods. The completed development fits and thresholds remain fixed. Moving blocks retain dependence from overlapping KPI observations and shared constituents of adjacent HDFS transitions. Block lengths are selected using development information before test-label comparison, and sensitivity is checked at half and twice those lengths.
Table 2 now reports observed test F1, absolute and relative gains, pointwise paired intervals, and Bonferroni-adjusted intervals for a six-comparison family. This family includes ADS at both AIOps budgets and DeepLog and PLELog at both HDFS budgets. The displayed strongest-baseline differences are 0.112, 0.061, 0.080, and 0.049 F1. Their adjusted intervals are [0.060–0.166], [0.018–0.103], [0.057–0.103], and [0.027–0.072], respectively.
The previous Cliff’s δ values were inconsistent with the reported non-overlapping percentile intervals if both summaries referred to the same bootstrap distributions. We have corrected this reporting by replacing them with direct F1 differences and their paired uncertainty intervals. Bootstrap replicates are no longer treated as independent experimental observations for hypothesis testing.
We have also separated test-sample uncertainty from sensitivity to learning. Table 2 concerns completed fits. Recalibration sensitivity is assessed through the separately reported initialisation repetitions, weak-label-subset experiment, and component ablation. The earlier full-pipeline bootstrap rows and the conclusions based on their small mean shifts have been removed. The revised statistical conclusions concern the paired comparisons under the specified evaluation protocol.

8. “Please describe how DeepLog, PLELog and ADS were run, tuned, and adapted to the 1%/5% labels and the quantile threshold. Please also comment on why their HDFS F1 values are well below those in their original papers. Invariant Mining (Lou et al., USENIX ATC 2010) is the classic invariant-based HDFS detector and very close to this paper's idea, so it should at least be cited and discussed. Recall, false-positive rate and AUROC/AUPRC would also help readers judge the comparison.”
Response: Thank you for requesting a more explicit description of the baseline experiments and their relationship to the original studies.

We have expanded the experimental section to document implementation provenance, retained components, regime-level score construction, use of disclosed and unlabelled development records, configuration searches, selected settings, and stochastic seeds. DeepLog evaluates the log-key prediction component using the existing LogHub templates. PLELog retains semantic representations, probabilistic label estimation, and the attention-based GRU. ADS retains shape-based transfer, detector-severity features, and CPLE with a random-forest classifier. Its adaptation to window-level labels, representative-stream selection, missing-history handling, and cross-fitting purge is now specified.

All methods use the common partitions and disclosed-label budgets. The 1% and 5% values do not denote test flagging quotas. Decision cutoffs are selected using pooled disclosed out-of-fold predictions after nominal-reference percentile calibration and are fixed before test scoring. The manuscript also clarifies that the k-means repetitions do not constitute independent neural-baseline retraining.

We have distinguished the present HDFS protocol from the original studies. We explicitly acknowledge that DeepLog originally performed well with a small normal training set. Label scarcity is therefore not presented as a sufficient explanation of the performance difference. The discussion identifies differences in preprocessing, training-session selection, session-start treatment, normal-anchor coverage, and calibration without attributing the entire gap to an untested individual mechanism.

We have added a diagnostic control applying the original fixed $g = 9$ decision rule to the same fitted DeepLog models. Its F1 values are 0.645 and 0.736, compared with 0.635 and 0.724 under development calibration. This identifies cutoff selection as a limited contributor within these models. It does not recover the published headline result or isolate preprocessing and training effects. The control is descriptive and does not enter test-based configuration selection or the primary paired-comparison family.

We have cited and discussed Invariant Mining by Lou et al. The text recognises invariant-based HDFS detection as established prior work. It specifies the present contribution through tolerance-based nominal-reference functionals, feature-weight calibration, and regime geometry.

Table 2.1 now reports precision, recall, false-positive rate, F1, AUROC, and AUPRC for all compared methods. It uses the same fitted models and test records as the primary Table 2 comparisons. The metric definitions, pooling, score-tie handling, and average-precision convention are specified. The accompanying analysis distinguishes anomaly coverage from false-positive burden and score-ranking performance from threshold-dependent decisions. The original ADS evaluation’s segment adjustment and test-selected best threshold are also distinguished from the present evaluation.

9. “Please state which data and sample Figures 1–3 show, and how they were produced.

- Fig. 3 is presented as the matrix of d_w, but its diagonal isn't zero and it doesn't appear symmetric, as a pseudometric should be. It also shows two equal-sized blocks, which doesn't fit a roughly 3% anomaly rate.
- The Fig. 2 legend mentions invariant-consistency encoding that I couldn't see in the plot.
- Statements such as "two thirds" and "a factor of one and a half" should be backed by the calculation."

Response: We thank the reviewer for identifying the insufficient documentation and inconsistencies in Figures 1–3. We have revised the figures and their accompanying descriptions to specify their data source, diagnostic sampling, construction, and numerical interpretation. We have retained their single-view format because they examine complementary properties of the same HDFS representation. Dataset comparisons are reported separately in the tables and Figure 4.

Figures 1 and 2 show 300 normal and 90 anomalous held-out HDFS regimes, sampled uniformly without replacement within each class using seed 2026. The representations use the selected 5% weak-supervision model with k-means initialization seed 42. Anomalies are deliberately overrepresented for visibility. The diagnostic proportion of 23.1% does not estimate the approximately 2.93% prevalence of the complete HDFS test set. Test labels are used for diagnostic sampling and interpretation, without entering model fitting, model selection, or threshold calibration.

The two-dimensional and three-dimensional projections use a documented custom UMAP implementation. Each is fitted to the same 600-regime development reference set, with 15 neighbours, a minimum distance of 0.1, and seed 42. The reference size limits visualization cost and does not reduce the detector's training set. The test regimes are transformed with the reference embedding held fixed. The axes are now labelled. Figure 2 visibly encodes $c_r = 1 - \frac{1}{3}\sum_m v_m(\delta_r)$ through marker area, with an explicit size mapping and numerical legend.

Figure 3 has been regenerated directly from the original weighted vectors using d_w with $q = 2$. Its zero diagonal and symmetry have been verified within 10^{-12}. It contains 45 normal and 45 anomalous regimes drawn from the diagnostic sample using seed 2027. Identical class ordering is applied to both axes. The equal-sized groups therefore reflect balanced display sampling rather than anomaly prevalence or cluster sizes. The revised analysis separately reports the three pair types and quantifies residual cross-class proximity.

The numerical descriptions now follow explicit calculations in the original representation space. The two clusters containing the largest numbers of sampled anomalies contain 60/90=66.7% of anomalies and 96/300=32.0% of normal regimes. Their concentration ratio is 2.08. This is identified as a descriptive calculation for post hoc selected groups. The centroid displacement divided by the mean normal radius is 1.04/0.70=1.49, supporting 'approximately one and a half'.

The statement concerning invariant-consistent anomalies is supported by 18/90=20.0%, using a nominal-development reference of $t_c = 0.9561$.

The revised interpretation distinguishes these diagnostic observations from performance evidence. It explains mixed cluster membership and heterogeneous anomalous representations without attributing specific failure mechanisms or inferring detection effectiveness from projected separation. Quantitative performance and component contributions are supported by the complete-test and ablation results.

10. "The paper emphasizes combining logs and metrics, but each experiment uses only one modality (p. 30). Please tone down this claim, or clearly mark joint evaluation as future work."

Response: We agree that the reported experiments evaluate one modality at a time. We have revised the Abstract, contribution statement, dataset description, implementation overview, and Conclusions to distinguish the common mathematical formulation from its separately evaluated implementations. Ambiguous references to simultaneous use, joint use, and cross-modality validation have been replaced with explicit descriptions of separate log-based and metric-based evaluation.

HDFS evaluates the log-derived representation, whereas AIOps evaluates the metric-derived representation. Both implementations use the proposed principles of regime construction, weakly supervised feature weighting, weighted geometry, and invariant regularisation. Their features, nominal references, calibrated weights, and fitted models are determined separately. The results support applicability of the framework to both observation modalities without establishing an advantage from combining them.

Joint evaluation is now explicitly identified as future work. It requires synchronized logs and metrics from the same system. We specify a comparison of log-only, metric-only, and combined representations using identical regime labels, splits, weak-label subsets, and threshold-selection rules, with each configuration calibrated on development data. This design will assess the incremental detection benefit of the combined representation relative to the stronger single-modality configuration.

11. "11. A few pipeline details need clarifying:
- For HDFS, how are the independent block sessions ordered to form "transitions," and how is a transition labelled when one side is normal and the other anomalous?
- How are KPI windows labelled?
- How are zero difference vectors handled in the angular similarity?
- How does the method avoid giving low scores to anomalies that form their own compact cluster?"

Response: We thank the reviewer for requesting these pipeline details. We have expanded the implementation description to specify session ordering, transition labels, window labels, initial references, and degenerate-vector handling. We have also corrected the interpretation of prototype-based scoring.

HDFS sessions are ordered separately within each partition by their first raw-event timestamp. Ties are resolved by original log position and numerical Block ID. This sequence compares successive observed block executions rather than representing a causal transition of the same block. Each difference inherits the destination-session label. Thus, normal-to-anomalous pairs are anomalous and anomalous-to-normal pairs are normal. A large difference does not itself determine the ground-truth label.

The first session is compared with a nominal reference estimated from disclosed normal development session representations. Cross-validation uses training-fold references, and final test evaluation uses a fixed development reference. This preserves one representation per session without forcing the first difference to zero or pairing individual sessions across partition boundaries.

For AIOps, a 60-sample window is anomalous if it contains at least one supplied anomalous point. Differences are constructed between consecutive windows of the same series within the same partition and inherit destination-window labels. Overlapping windows remain separate evaluation records, without point adjustment. Initial windows use development-derived nominal descriptor references, as specified in the revised implementation.

Angular degeneracy is checked on the final weighted representation using a norm tolerance of 10^{-12}. A zero raw difference need not produce a zero final representation. Directionally degenerate regimes remain in the evaluation set. Their final score is twice the mean of the three bounded invariant violations, placing the fallback in the angular score's [0,2] range. The complete scoring rule is included in development threshold calibration. The revised description also defines assignment ties and reinitialization of degenerate prototypes.

We agree that a compact anomalous development cluster can receive low prototype-proximity scores. Invariant regularisation and weak-label consistency affect the learned representation but do not guarantee high scores for every anomalous cluster. We have stated this limitation and removed the interpretation that every fitted prototype necessarily represents nominal behaviour. The experimental results support detection performance in the evaluated settings. A nominal-prototype restriction or calibrated cluster-risk component would constitute an extension requiring separate validation.

12. "Please share the code and derived data: processed features, labels, weak-label subsets, splits, selected hyperparameters and per-run results. Please also report runtime and hardware. Without these the tables and figures can't be checked, which the PLOS data policy expects."

Response: Thank you for this recommendation. The computational materials are now supported by a publicly archived Python reference implementation at https://doi.org/10.5281/zenodo.23218142, distributed under the GNU General Public License, version 3 or later. The Data Availability Statement identifies this software deposit together with the public sources of the experimental observations and labels.

The implementation includes observation representation, weighted geometry, spherical clustering, geometric and invariant regularisation, dataset-specific invariant functionals, weak-label cross-fitting, threshold calibration, and evaluation utilities. Installation instructions, two runnable examples, and 50 automated tests accompany the package. Its modular structure allows individual mathematical components to be inspected and tested before executing the complete workflow.

The experimental inputs are available from the 2018 AIOps Challenge KPI repository at https://github.com/NetManAIOps/KPI-Anomaly-Detection and the HDFS collection in LogHub at https://github.com/logpai/loghub/tree/master/HDFS. Section 3 identifies the relevant HDFS input files and specifies event-template coordinates, occurrence and frequency descriptors, directed changes, and label assignment. For KPI observations, it defines window length, stride, robust standardisation, descriptor calculation, and transition labels. The nominal-reference estimation rules and all six invariant functionals are also specified. These definitions make the transformation from public observations to model inputs explicit.

The revised experimental protocol provides development and test sizes, class compositions, disclosed-label counts, nested annotation budgets, cross-fitting exclusions, candidate grids, stopping criteria, and threshold calibration. Selected reference configurations and their frequencies across initialization repetitions are reported separately from the additional weak-label-subset experiment. These details identify the conditions under which the comparisons were conducted and distinguish label availability from test prevalence and the proportion of flagged regimes.

The processed features are computational representations of the public benchmark observations. Section 3 specifies their construction through event-template statistics for HDFS and window-level descriptors for KPI data, followed by directed differences and development-fitted transformations. The archived implementation exposes these operations and supports exporting the resulting representations with record identifiers, allowing their construction to be independently inspected.

The software supports exporting processed features, disclosed labels, preprocessing parameters, invariant references, learned weights, prototypes, selected configurations, thresholds, scores, predictions, and evaluation metrics. Input manifests identify records and their partition membership. Prediction files associate decisions with record identifiers and evaluation labels. Run metadata retain calibration outputs and convergence information. Researchers can therefore execute the workflow on the public inputs and retain intermediate quantities for independent inspection.

The manuscript additionally provides direct numerical checks on the reported results. The reference-fit confusion matrices determine the proposed-method precision, recall, and F1 values. Figure 4 and Tables 2–2.1 use the same reference-fit predictions and evaluation definitions. Initialization variability, weak-label-subset sensitivity, and component ablation are reported as separate analyses. The paired-bootstrap description specifies matched resampling, segment boundaries,

block-length selection, and interval construction. This separation prevents variation from refitting from being conflated with uncertainty conditional on completed models.
The proposed-method experiments used a workstation with an AMD Ryzen 9 7950X processor, 64 GB RAM, and NVMe storage, running Ubuntu 22.04 LTS and Python 3.11. The proposed method used CPU execution. These hardware details concern the proposed method and do not imply identical execution environments for the neural baselines.
The archived implementation records elapsed execution time and environment information in its run metadata, allowing runtime to be measured for the supplied inputs and configuration. Section 2 provides the complementary complexity analysis. It distinguishes the quadratic worst case of exhaustive pairwise geometry from prototype-based fitting and scoring. The study evaluates an offline analytical workflow, and its numerical results are not presented as evidence of streaming latency or production throughput.
The public observations and labels, the operational specification in Section 3, and the archived implementation provide an executable basis for independent investigation. The supplied examples demonstrate the execution path, while the manuscript defines the dataset-specific experimental settings used to assess the framework.

Response to the Minor Commrnts:

1. Incorrect baseline citations

Thank you for identifying the citation mismatch. DeepLog, PLELog, and ADS are now linked to references [21], [22], and [36], respectively. Their descriptions distinguish the original methods from the adaptations used in the common evaluation protocol.

2. Missing LogLLM and LogADReFT references

We have added the references for LogLLM and the parameter-efficient log anomaly detection study implemented in LogADReFT. The experimental description now distinguishes the implementation name LogADReFT from the evaluated Llama3-ReFT configuration. This preserves the correspondence between the cited method and the specific model reported in Table 3.

3. References that do not support their citations

We have replaced the references on educational learning environments, social-science research methods, and stock-price prediction with sources addressing automated system-log analysis, anomaly detection, and deep anomaly detection. The telemetry paragraph also cites the relevant metric- and trace-based studies. The revised references therefore support the methodological statements for which they are used.

4. Multivariate versus univariate KPI data

The dataset is now consistently described as 29 individually labelled univariate KPI series. Pooling their regime-level predictions for evaluation does not make the input to each series-specific descriptor calculation multivariate. The revised wording separates dataset composition from the dimensionality of the constructed regime vectors.

5. KPI sampling intervals

We have clarified how the native sampling interval is identified for each KPI series and how it determines the physical duration of the evaluated windows. The native intervals are 60 or 300 seconds. Larger timestamp gaps are distinguished from the native cadence when defining uninterrupted segments. Window length and stride remain 60 and 15 observations, respectively. They therefore correspond to 60-minute windows with 15-minute strides or 300-minute windows with 75-minute strides. The 75% overlap is unchanged. This clarification makes explicit that the descriptor construction uses a common observation count while the physical duration of a regime depends on the series cadence.

6. Leftover LaTeX fragments

We have removed the residual LaTeX fragments and corrected the remaining escaped brace in the ADS parameter description. Mathematical symbols are presented as formatted notation, and discrete parameter alternatives are written directly in the text.

7. Reference to the overall optimisation scheme

The reference has been corrected to Eq. (11). Equation (10) defines the inner clustering problem, whereas Eq. (11) incorporates that problem into the overall representation-calibration and model-selection formulation.

8. HDFS session count

The manuscript now consistently reports 575,061 HDFS sessions. The reported nominal and anomalous counts, as well as the partition counts, sum to this total. The correction therefore aligns the corpus description with the experimental accounting.

9. Figure axes and embedding method

Figure 1 now labels its axes UMAP-1 and UMAP-2. The preamble to Figure 2 explicitly identifies a separate three-dimensional UMAP embedding fitted to the same development reference sample. The implementation, fitting settings, and transformation procedure are described in the figure preambles. The numerical geometric analysis uses the original weighted regime coordinates rather than distances measured in the projections.

10. Interpretability rating in Table 1

We have replaced the qualitative interpretability ratings with descriptions of the explicit analytical structure exposed by each approach. For the proposed

framework, these quantities are invariant violations, feature weights, distances, and regime prototypes. Table 1 consequently compares defined methodological properties rather than presenting an unevaluated ranking of human interpretability.

11. Section 2.2 heading
The heading has been changed to "Invariant-Guided Geometric Model for Detection of Anomalous Execution Regimes." This matches the evaluated target. An anomalous execution regime describes a departure in observed operational behaviour and does not, by itself, establish a particular software error.

12. Use of "static analysis"
We have replaced "static analysis" with "offline batch evaluation." The revised description specifies that recorded observations are processed without online adaptation or incremental parameter updates. This terminology distinguishes the experimental execution mode from source-code static analysis.

13. Invariant-regulariser notation and zero violations
The invariant regulariser is consistently expressed as $R_{\text{inv}}(\mathbf{M})$, where $\mathbf{M}$ is the empirical collection of regime vectors produced by Φ. We have also made the condition $\varepsilon > 0$ explicit. A family with zero violation values contributes zero because its numerator vanishes while its denominator remains positive. When every family has zero violations, the complete invariant regulariser is zero. The formulation therefore remains defined without introducing an artificial invariant contribution.

14. Keyword "automated software testing"
We have removed "automated software testing" and revised the keywords to reflect operational anomaly detection, execution regimes, weighted geometry, weak supervision, and log and KPI analysis.

15. Privacy
We have added a paragraph on privacy-aware preprocessing of production telemetry. It identifies the need for data minimisation and appropriate redaction before feature construction and sharing. It also notes that templates and aggregated descriptors require inspection because aggregation alone does not guarantee anonymisation. This addresses privacy at the input and representation stages without assuming that the public benchmark observations establish privacy protection for a future deployment.

Responses to the Questions for the Authors:

1. Which set are the Table 2 and Fig. 4 metrics computed on, and what is its anomaly rate?
Table 2 and Figure 4 use the same independent test sets and reference-fit predictions. The HDFS test set contains 115,013 Block-ID sessions, comprising

111,645 normal and 3,368 anomalous sessions. Its anomaly rate is 2.928%. The AIOps test set contains 20,000 window-based regimes, comprising 18,933 normal and 1,067 anomalous regimes. Its anomaly rate is 5.335%. Test membership and ground-truth labels remain identical at both supervision levels. Disclosed development regimes are excluded from test scoring by partition membership. The reported confusion matrices provide the underlying counts for the proposed-method precision, recall, and F1 values.

2. Do “1%” and “5%” mean the labelled fraction, the threshold, or both?
They denote development-label disclosure budgets. The specified fraction is applied separately to anomalous and nominal development records, with counts rounded down. Neither value specifies the fraction of test regimes flagged as anomalous.
Decision thresholds are selected using pooled, cross-fitted development predictions after nominal-reference percentile calibration. They are fixed before test evaluation. The resulting test alert fractions are 2.165% and 2.673% for HDFS, and 4.035% and 5.000% for AIOps, at the respective 1% and 5% disclosure budgets. The 5.000% AIOps alert fraction is an observed outcome rather than an imposed quota.

3. Which k, λ1 and λ2 were selected? Was λ2 above zero?
The selected reference configurations (k,λ_1,λ_2) are (3,0.05,0.10) for AIOps at 1%, (4,0.05,0.10) for AIOps at 5%, (4,0.10,0.20) for HDFS at 1%, and (6,0.05,0.20) for HDFS at 5%. Here, k is the number of clusters, λ_1 controls geometric regularisation, and λ_2 controls invariant regularisation. These configurations were selected in 17, 18, 16, and 17 of the 20 initialisation repetitions, respectively. The fully labelled HDFS comparison in Table 3 uses (6,0.05,0.10).
Thus, $\lambda_2 > 0$ in every reported reference configuration. Positive fitting-regime violations also yield a non-zero invariant penalty. This establishes that the term participates in calibration. Its predictive contribution is assessed separately through component ablation.

4. Where are the ablation results mentioned in the Conclusions?
The results are now included in Section 3, Table 4, with the experimental specification preceding the table and the interpretation following it. The Conclusions explicitly refer to these results.
The experiment separately removes invariant regularisation, removes geometric regularisation, and replaces learned metric weights with uniform weights. The uniform-weight intervention concerns the metric-weight vector. Descriptor-aggregation coefficients and invariant-family aggregation remain shared across variants. Each intervention is evaluated under both the paired full-model configuration and development-based retuning, using identical partitions, disclosed-label subsets, cross-fitting folds, and 20 initialisation seeds.

After retuning, removal of invariant regularisation reduces mean test F1 by 0.024–0.041 across the four settings. Removal of geometric regularisation produces losses of 0.008–0.017. Uniform metric weights produce losses of 0.030–0.057. The full model retains the highest mean F1 in every evaluated setting. These results support the revised Conclusions concerning the contribution of the three calibration components.

5. Why doesn't Fig. 3 have a zero diagonal, and what data does it show?
The previous rendering did not correctly represent the stated pseudometric matrix and has been replaced. Figure 3 is now computed directly from the original weighted regime vectors using d_w with $q = 2$. Its zero diagonal and symmetry have been verified within 10^{-12}.
The figure contains 45 normal and 45 anomalous held-out HDFS regimes drawn from the diagnostic sample used for Figures 1–2. The representation is produced by the 5% supervision model with initialisation seed 42. Identical regime ordering is used on both axes.
Balanced sampling makes within-normal, within-anomalous, and cross-class distances readable within one matrix. The equal-sized blocks therefore describe the display composition. They do not represent cluster sizes or the approximately 2.93% anomaly prevalence of the complete HDFS test set. The revised preamble specifies the sampling and construction procedure, and the accompanying analysis reports the three pairwise-distance categories separately.

6. How were Cliff's δ values of 0.50–0.64 obtained when the intervals don't overlap?
The previous reporting did not establish a reproducible correspondence between the stated Cliff's δ values and the bootstrap distributions underlying the reported intervals. Those values therefore do not provide a defensible effect-size summary for the comparison. We have removed them, their qualitative effect-size categories, and the Wilcoxon-derived p-values.
Table 2 now reports observed paired F1 differences and moving-block bootstrap intervals. Each replicate uses the same resampled test-regime identifiers and multiplicities for both methods, while fitted models and thresholds remain fixed. Bootstrap replicates are used to estimate uncertainty and are not treated as independent experimental observations.
For AIOps, the observed F1 advantages over ADS are 0.112 and 0.061 at 1% and 5% supervision. Their pointwise 95% paired intervals are [0.075, 0.150] and [0.033, 0.092]. The corresponding intervals adjusted for the six-comparison family are [0.060, 0.166] and [0.018, 0.103]. The revised analysis directly quantifies the performance difference and its uncertainty without relying on the previous rank-based summaries.
