# Supplementary material for: An invariant-guided model for automated detection of anomalous execution regimes from production telemetry
Source: PLoS One. 2026 Nov 12;21(11):e0362583. doi: 10.1371/journal.pone.0362583 (PMC13759071; doi:10.1371/journal.pone.0362583)
Supplement: Supplementary file 3 [file pone.0362583.s003.doc]

**RESPONSES TO THE ACADEMIC EDITOR AND REVIEWERS**

**Responses to the Academic Editor**

We sincerely thank the Academic Editor for the careful consideration of our manuscript and for the thoughtful synthesis of the Reviewers’ comments. We greatly appreciate the clear and constructive editorial guidance, which helped us identify the principal areas requiring further clarification, methodological transparency, empirical strengthening, and more precise delimitation of the scope of our conclusions. The Editor’s recommendations provided a coherent direction for the revision and enabled us to address the individual Reviewers’ concerns within a more consistent methodological and practical perspective. We have carefully considered each of the points raised and have revised the manuscript accordingly, as summarised below.

The reviewers requested a clearer description of the framework's objectives, the integration of heterogeneous telemetry sources, the evaluation methodology, and the framework's role within existing observability infrastructures. They also emphasized the need for comprehensive citation of all data sources, methods, and related studies, together with a more transparent description of feature construction, weight calibration, and hyperparameter selection to improve reproducibility.

In addition, the manuscript should provide a stronger assessment of computational complexity, runtime overhead, scalability, and deployment feasibility in large-scale cloud-native environments. The evaluation would benefit from comparisons with additional recent deep learning and foundation model-based approaches, while the limitations section should explicitly address challenges related to online adaptation, concept drift, streaming telemetry, and real-time deployment. Finally, the reviewers recommended reducing unnecessary mathematical detail, emphasizing the practical workflow, and validating the framework on more diverse industrial datasets and production settings to strengthen evidence for its generalizability and practical applicability.

**Response**: We sincerely thank the Academic Editor for synthesising the Reviewers’ comments and for identifying the principal areas in which the manuscript required clarification and strengthening. We have carefully addressed these points throughout the revised manuscript.

First, we have clarified the principal objective, telemetry-integration mechanism, and practical positioning of the proposed framework. The revised manuscript now makes explicit that heterogeneous telemetry is integrated at the level of structural execution-regime representation rather than by homogenising raw logs and KPI observations, and that the framework operates as an interpretable analytical layer above existing production observability infrastructures rather than replacing their acquisition, monitoring, orchestration, or incident-management functions.

Second, we have substantially strengthened methodological transparency and reproducibility. The revised experimental section now provides deterministic definitions of KPI- and log-side features, the complete processing sequence from regime construction to the final regime vector, the feature-weight calibration procedure, parameter constraints, stopping criteria, hyperparameter search spaces, model-selection rules, and deterministic tie-breaking procedures. We additionally evaluated sensitivity to spherical k-means initialisation over 20 independent repetitions. Source attribution was also re-checked throughout the manuscript to ensure that datasets, baseline methods, methodological components, and externally derived claims are appropriately referenced.

Third, we have added an explicit computational-complexity analysis and more carefully delimited the scalability and deployment claims. The revised manuscript identifies exhaustive pairwise geometry as the principal worst-case scalability bottleneck of a naïve implementation and distinguishes analytical complexity from production runtime performance. Since wall-clock latency, peak memory consumption, throughput, telemetry-ingestion overhead, and live cloud-native scalability were not measured, the manuscript now explicitly avoids inferring real-time production feasibility from the reported offline experiments.

Fourth, the empirical evaluation has been extended with a separate controlled HDFS comparison against LogGPT, LogLLM, and Llama3-ReFT under a common session-level protocol. The results broaden the contemporary empirical context while also narrowing the comparative claim: the proposed framework exceeds LogGPT under this protocol but does not outperform the higher-capacity LogLLM and Llama3-ReFT models. The revised discussion reflects this result without claiming universal predictive superiority.

Fifth, the limitations section now explicitly addresses online adaptation, concept drift, streaming telemetry, controlled recalibration, dynamic maintenance of regime geometry, and the additional computational validation required for real-time deployment. The theoretical presentation has also been reviewed for concision, while the mathematical definitions required for methodological completeness and reproducibility have been retained. To strengthen practical readability, we now present the framework explicitly as a sequential workflow from production telemetry through regime construction, weighted geometry and invariant-guided regularisation to clustering and anomaly scoring.

Finally, we have clarified the empirical boundary of the present validation. The AIOps and HDFS experiments demonstrate that the same mathematical formalism can be operationalised across continuous KPI dynamics and discrete log-event structure, but they do not establish unrestricted cross-environment generalisability. The revised manuscript now states this distinction explicitly and identifies validation on independently collected telemetry from heterogeneous industrial architectures and production observability stacks as an important next stage.

We believe that these revisions have substantially strengthened the methodological transparency, reproducibility, empirical positioning, practical interpretation, and delimitation of the proposed framework, while ensuring that the conclusions remain commensurate with the evidence provided by the present study. We sincerely thank the Academic Editor and the Reviewers for their careful assessment and constructive guidance.

**Responses to Reviewer #2**

We sincerely thank Reviewer #2 for the careful, constructive, and technically insightful assessment of our manuscript. The comments helped us improve the reproducibility, experimental validation, computational analysis, practical interpretation, and limitations of the proposed framework. We have addressed each point carefully and believe that the resulting revisions have substantially strengthened the rigour, clarity, and empirical positioning of the manuscript.

1. The manuscript should better quantify the computational complexity, runtime overhead, and scalability of the proposed framework, particularly for large-scale cloud-native production environments.

**Response**: We thank the Reviewer for this important comment. We agree that the original manuscript did not distinguish sufficiently between the computational complexity of the mathematical framework and the runtime characteristics of a production implementation. We have revised the manuscript accordingly.

First, we have added an explicit complexity analysis of the principal computational stages. For $N$ regime vectors of dimensionality $p$, one weighted-pseudometric evaluation requires $O(p)$ operations, whereas exhaustive evaluation of all regime pairs has a worst-case cost of $O(N^{2}p)$ and requires $O(N^{2})$ memory if the complete distance matrix is materialised. We therefore identify exhaustive pairwise geometry, rather than feature representation or prototype-based scoring, as the principal scalability bottleneck of a naïve implementation. The revised manuscript also specifies how restricting the regularisation set to $m$ sampled pairs changes the corresponding distance-evaluation cost to $O(mp)$, and reports the complexity of spherical k-means and prototype-based anomaly scoring as $O(NkpT)$ and $O(Nkp)$, respectively. We have also clarified that neighbourhood-based formulations reduce the number of retained pairwise relations, while the cost of constructing those neighbourhoods depends on the neighbour-search strategy.

Second, we have explicitly separated these analytical bounds from empirical runtime claims. The experiments in this study were designed as an offline validation of the proposed mathematical framework and did not measure wall-clock latency, peak memory consumption, throughput, or telemetry-ingestion overhead under a live cloud-native workload. We therefore do not claim that the present implementation has demonstrated real-time production scalability. Instead, the revised manuscript identifies the computational stage that would require sampled, indexed, approximate, or incremental treatment in a production-oriented implementation.

Finally, to ensure that the scope of the conclusions is consistent with the available evidence, we have replaced the qualitative statement “High for offline batch analysis” in Table 1 with “Suitable for offline batch analysis; production-scale computational performance not evaluated.

2. Although the mathematical framework is rigorous, several implementation details (e.g., feature construction, weight calibration, and hyperparameter selection) should be described more explicitly to improve reproducibility.

**Response**: We thank the Reviewer for this constructive comment. We agree that, although the mathematical framework was formulated in considerable detail, the original manuscript did not describe several implementation-level procedures with sufficient operational specificity for independent reproduction. We have therefore substantially revised Section 3 to establish a direct and deterministic correspondence between the formal constructs introduced in Equations (1)–(11) and their experimental implementation.

First, the feature-construction procedure has been specified explicitly for both experimental domains. For the AIOps KPI benchmark, each execution regime is represented by a fixed temporal window, and the revised manuscript now defines the four metric-side descriptors operationally. The within-window level is calculated as the median of the robustly standardised KPI values; the inter-window deviation as the difference between the medians of two consecutive windows; the local temporal slope as the least-squares regression coefficient of the standardised values against their within-window time indices; and within-window variability as the interquartile range. For HDFS, the revised manuscript now specifies the normalised event-template occurrence representation, the signed change in corresponding template frequencies between consecutive block-trace representations, the preservation of template identity through fixed coordinates, and the treatment of absent template occurrences as zero-valued coordinates. We have also clarified the exact processing order from regime construction and directed structural differencing to descriptor evaluation, logarithmic compression where applicable, robust standardisation, and assembly of the final regime vector.

Second, the feature-weight calibration procedure has been made explicit. Feature weights are initialised uniformly on the probability simplex and calibrated using deterministic projected coordinate search. Starting from $w_{i}=1/p$, each coordinate is perturbed successively while preserving the constraints $w_{i}\geq0$ and $\sum_{i} w_{i}=1$. For every admissible candidate weight vector, the inner spherical k-means problem is refitted and the complete integrated criterion in Equation (11) is recomputed. Candidate updates are accepted only when they decrease the criterion. The revised manuscript now specifies an initial coordinate step of $h=0.10$, step halving after a complete pass without improvement, termination at $h<10^{-3}$ or after 100 complete coordinate passes, and a fixed feature-processing order. This removes the ambiguity present in the original statement that the weights were merely “updated under the constraints”.

Third, we have made the hyperparameter-selection procedure fully explicit. The number of clusters is evaluated over $k\in\{2,\ldots,12\}$, while the regularisation coefficients $\lambda_{1}$ and $\lambda_{2}$ are evaluated over the Cartesian product of $\left\{0,0.05,0.10,0.20,0.50\right\}$. For every candidate triplet $(k,\lambda_{1},\lambda_{2})$, the feature-weight vector is recalibrated from the same uniform initialisation and the corresponding spherical k-means model is fitted. The candidate configuration attaining the lowest observed value of the complete integrated criterion is retained. The revised manuscript additionally specifies deterministic rules for resolving numerically equivalent configurations. We have also clarified that the geometric-component weights $\rho_{j}$, invariant-family weights $\eta_{m}$, and quality-component weights $\gamma_{c}$ are fixed at equal values in the reported experiments and are not subjected to additional undocumented optimisation.

To verify that the resulting implementation is not unduly dependent on clustering initialisation, we additionally repeated the complete calibration and model-selection procedure over 20 independent spherical k-means initialisations for each dataset and weak-supervision level, while holding all feature-construction rules, initial feature weights, hyperparameter grids, stopping criteria, and weak-label subsets fixed. The same discrete model configuration $(k,\lambda_{1},\lambda_{2})$ was recovered in 85% of repetitions for AIOps at 1% supervision, 90% for AIOps at 5%, 80% for HDFS at 1%, and 85% for HDFS at 5%. The corresponding standard deviations of F1 were 0.010, 0.008, 0.015, and 0.011, respectively. The mean absolute deviation of the calibrated feature weights from their repetition-wise mean vectors was 0.024, 0.018, 0.033, and 0.026, respectively. These results indicate that both the selected hyperparameter configuration and the resulting detection performance remain stable with respect to spherical k-means initialisation.

Finally, we have revised the previous general statement that the experimental specification eliminated implementation ambiguity. The revised manuscript now states more precisely that the experimental instantiation defines the data representation, deterministic feature-construction rules, fixed parameters and admissible search ranges, weight-calibration algorithm, stopping criteria, and model-selection procedure associated with Equations (1)–(11). We believe that these additions substantially improve the reproducibility of the proposed framework while preserving the intended generality of its mathematical formulation.

3. The experimental evaluation would be strengthened by including additional recent deep learning and foundation-model-based anomaly detection methods as baselines.

**Response**: We thank the Reviewer for this important suggestion. We agree that the original comparison, although designed around methodological proximity and scarce-supervision conditions, did not establish how the proposed execution-regime framework compares with recent high-capacity neural and foundation-model-based detectors. We have therefore substantially extended the experimental evaluation by adding three recent methods representing complementary contemporary architectures: LogGPT, a GPT-based generative event-sequence detector with reinforcement-learning adaptation; LogLLM, which combines BERT-based semantic encoding with a Llama-based sequence classifier; and Llama3-ReFT, which applies representation fine-tuning to a Llama 3 8B backbone.

Rather than inserting these methods into the original 1% and 5% weak-supervision experiment, we introduced a separate controlled HDFS comparison immediately after the analysis associated with Table 2. This distinction is deliberate. The original experiment evaluates robustness when labelled anomalies are scarce and the operating point is constrained by fixed upper-tail anomaly rates, whereas the new experiment evaluates session-level discriminative capacity under a common labelled HDFS protocol. Combining the two would confound supervision availability and decision calibration with differences in model architecture and representational capacity.

To make the new comparison controlled and reproducible, all methods operate on the same HDFS_v1 corpus and the same Block-ID execution sessions. Partitioning is performed before model-specific preprocessing and is stratified by the binary session label. A common 72/8/20 fitting–validation–test split is used, with identical Block-ID membership for every method. Model-specific representations and reference preprocessing are retained because they constitute integral parts of the respective detectors, while the underlying sessions, labels, partition membership, evaluation unit, and final metrics are held constant. No test labels are used for feature calibration, model fitting, hyperparameter selection, neural adaptation, clustering, or decision-threshold selection. For the proposed method, its formal regime representation is unchanged; $q=2$, $k_{nn}=10$, and the equal $\rho$, $\eta$, and $\gamma$ coefficients are retained, while $k$, $\lambda_{1}$, $\lambda_{2}$, and $w$ are recalibrated using only fitting and validation data.

We also introduced a common external session-level evaluator because the reference implementations do not all employ identical internal averaging conventions. Anomalous sessions are treated as the positive class, and Precision, Recall, and F1 are calculated identically from one binary prediction per Block ID. For the proposed continuous anomaly score, the operating threshold is selected exclusively on validation data and then frozen for test evaluation. Each complete experiment is repeated over 3] independent random seeds while retaining identical data partitions.

The results are now reported in the new Table 3. Under this common protocol, the proposed framework achieves an F1 score of 0.921 ± 0.015, compared with 0.904 ± 0.031 for LogGPT, 0.995 ± 0.002 for LogLLM, and 0.986 ± 0.005 for Llama3-ReFT. Thus, the proposed method exceeds LogGPT by 0.017 F1, while trailing LogLLM and Llama3-ReFT by 0.074 and 0.065, respectively. We have explicitly interpreted this result without claiming universal superiority: the higher performance of the Llama-based methods is consistent with their ability to retain high-capacity pretrained semantic and long-range contextual representations, whereas the proposed framework deliberately constrains the representation to explicit event composition, directed regime change, weighted geometry, and invariant consistency. The observed F1 differences therefore characterise the predictive component of this representational difference; they are not used to infer interpretability, computational efficiency, robustness, or deployment suitability.

We further clarify in the revised manuscript why the proposed method attains a higher HDFS F1 in Table 3 than in Table 2. The values of 0.722 and 0.765 in Table 2 correspond to deliberately restrictive 1% and 5% weak-supervision conditions with fixed upper-tail anomaly-rate decision rules, whereas Table 3 uses the labelled development partition for calibration and an independently selected validation operating point. The two experiments consequently quantify complementary properties: robustness under label scarcity and constrained alert rates in Table 2, and discriminative capacity against recent high-capacity neural detectors under a common HDFS session-level protocol in Table 3.

Accordingly, the revision both broadens and narrows the empirical positioning of the proposed framework. It broadens the evidence by adding recent GPT- and Llama-based comparators, but narrows the comparative claim by showing explicitly that the proposed framework does not universally outperform foundation-model-based detectors. Instead, it remains competitive with the recent generative LogGPT baseline while providing an explicitly defined execution-regime space in which structural changes, distances, neighbourhood relations, and invariant inconsistencies remain directly represented. We believe that this additional experiment addresses the Reviewer’s concern more rigorously than a direct insertion of recent architectures into the original weak-supervision comparison, because the competing systems are evaluated under a common observational population and metric definition while retaining their native representational mechanisms.

4. The manuscript should discuss the limitations of the proposed approach more explicitly, particularly regarding online adaptation, concept drift, streaming telemetry, and real-time deployment.

**Response**: We thank the Reviewer for this important comment. We have substantially expanded the discussion of limitations in the Conclusions to distinguish explicitly between the offline analytical formulation validated in this study and the additional mechanisms that would be required for streaming and real-time operation.

Specifically, the revised manuscript now clarifies that the regime geometry, cluster configuration, feature weights, and invariant-guided regularisation are calibrated from a fixed observation set, while the present formulation does not define incremental updates of $k$, $w$, cluster representatives, or neighbourhood relations as new telemetry arrives. We therefore explicitly state that online adaptation cannot be inferred from the reported offline results.

We have also added a separate discussion of concept drift. Persistent changes in workload, software configuration, deployment state, or nominal execution behaviour may alter both the distribution of regime features and the empirical validity of previously calibrated invariant relationships. The revision identifies two corresponding failure modes: a new nominal operating regime may initially be interpreted as an anomalous structural transition, while progressive drift may reduce the separation between nominal and anomalous regimes. We therefore identify explicit drift detection and controlled recalibration as necessary extensions of the current framework.

The revised manuscript further specifies what streaming adaptation would require: online construction of regime transitions, bounded-state or incremental computation of feature and invariant statistics, dynamic maintenance of neighbourhood relations and cluster representatives, and a criterion for distinguishing transient anomalies from persistent distributional drift before recalibration. Importantly, we also clarify that continual adaptation cannot simply be implemented by updating the geometry from every recent observation, because indiscriminate adaptation could incorporate anomalous behaviour into the nominal regime geometry.

Finally, we have delimited the real-time deployment claim more explicitly. The present framework does not define incremental optimisation of functional (11) or real-time maintenance of regime geometry under continuous telemetry ingestion. This methodological limitation is distinguished from the computational limitations discussed separately in the revised Conclusions, where we state that wall-clock latency, peak memory consumption, throughput, and telemetry-ingestion overhead have not been evaluated under a live cloud-native workload. Accordingly, the manuscript no longer implies that the reported offline results establish real-time production scalability.

We therefore position the current framework as an offline invariant-guided analytical layer that complements existing production observability and operational-management infrastructure. Incremental geometry, drift detection, controlled recalibration, streaming computation, and computational latency are now identified explicitly as interconnected requirements for future real-time deployment. We believe that these revisions address the Reviewer’s concern while avoiding unsupported claims about online capabilities that have not been experimentally evaluated in the present study.

5. Several theoretical sections are unnecessarily long and mathematically dense; condensing these sections while emphasizing the practical workflow would improve readability.

**Response**: We thank the Reviewer for this observation and agree that the connection between the formal construction and its practical analytical workflow should be more immediately visible. We have therefore reviewed the theoretical section for concision and strengthened the operational interpretation of the mathematical framework. In particular, repetitive explanatory material has been reduced where this could be done without removing definitions required by subsequent equations and by the experimental implementation.

We have, however, retained the core mathematical construction in Equations (1)–(11). This decision is deliberate because these expressions do not constitute independent theoretical elaborations: they form a sequential dependency chain from directed structural change and regime mapping, through weighted geometry and invariant regularisation, to clustering, model selection, and anomaly scoring. Removing individual components would make later quantities dependent on definitions that are no longer explicit and would reduce the self-contained character and reproducibility of the proposed method. This became particularly important in the revision because, in response to the Reviewers’ requests for greater reproducibility, the implementation of feature construction, weight calibration, invariant specification, and hyperparameter selection is now tied explicitly to the corresponding formal quantities.

To address the readability aspect of the comment directly, we have added a concise operational summary that presents the framework as a practical sequence: production telemetry → execution states and directed structural changes → regime vectors → weighted regime geometry → invariant-guided regularisation → calibrated clustering → anomaly scoring. This makes clear that Equations (1)–(11) describe successive computational stages rather than a collection of abstract mathematical constructions.

We also note that the experimental section has necessarily become more detailed in the revised manuscript. This expansion is not intended to compensate for, or extend, the theoretical exposition; it results directly from addressing the Reviewers’ requests for additional implementation details, reproducibility analysis, computational-complexity discussion, recent deep-learning and foundation-model baselines, a controlled common-protocol HDFS comparison, and fuller interpretation of the resulting evidence. Several of these additions cannot be represented adequately by a figure or table alone because their validity depends on the experimental protocol, controlled variables, evaluation conditions, and interpretation of the observed differences.

We have therefore sought to improve readability through structural compression and a clearer theory-to-workflow mapping rather than by deleting mathematical definitions required for methodological completeness. We believe this preserves the self-contained formulation of the method while making its practical sequence substantially easier to follow.

6. Additional validation on more diverse industrial datasets and production environments would provide stronger evidence of the framework's generalizability and practical applicability.

**Response**: We agree with the Reviewer that validation across additional industrial systems and production environments would provide stronger evidence of cross-environment generalisability. We have therefore revised the manuscript to delimit more explicitly what can and cannot be concluded from the present experimental design.

The two datasets used in this study were selected deliberately rather than as interchangeable benchmark instances. Both contain real operational or quasi-operational telemetry and avoid synthetic data generation, but they represent complementary observational modalities and operational tasks. The 2018 AIOps Challenge dataset provides 29 labelled KPI time series representing continuous metric-centred monitoring of resource, workload, and service-quality behaviour, whereas HDFS provides large-scale event-centred telemetry comprising more than 11 million log messages organised into approximately 575,000 labelled execution sessions. Their joint use was intended to test whether the same mathematical formulation can be operationalised across continuous KPI dynamics and discrete log-event structure rather than to reproduce the full diversity of contemporary production environments.

We have retained this two-domain design because adding a further dataset is not methodologically equivalent to adding another sample to an otherwise unchanged experiment. The framework is instantiated through modality-specific regime construction, feature mappings, structural differences, and invariant predicates. A genuinely different industrial environment or additional telemetry modality would therefore require corresponding operational definitions, preprocessing and invariant specification, together with an appropriate baseline and evaluation protocol. Introducing such an extension solely as an additional benchmark within the present revision would materially broaden the experimental design rather than provide an independent replication of the experiments already reported.

At the same time, we agree that the current evidence should not be interpreted as establishing unrestricted industrial generalisability. The revised manuscript now distinguishes explicitly between cross-modality validation, which is supported by the KPI and HDFS experiments, and cross-environment generalisation, which remains to be established. In particular, the Conclusions now identify independently collected telemetry from heterogeneous industrial architectures and production observability stacks as a required next validation stage, including assessment of whether the learned regime geometry and invariant relationships remain stable under changes in architecture, workload, instrumentation, and organisational operating conditions.

We have therefore chosen not to add a heterogeneous third dataset without the corresponding methodological redesign, but instead to narrow the generalisability claim and state explicitly the empirical boundary of the present study. We believe this provides a more rigorous interpretation of the available evidence: the current experiments demonstrate applicability across two substantially different operational telemetry modalities, while broader industrial and production-level generalisation remains an open empirical question requiring dedicated cross-environment validation.

**Responses to Reviewer #3**

We sincerely thank Reviewer #3 for the careful and constructive assessment of our manuscript and for the thoughtful questions and recommendations. The comments provided a valuable opportunity to clarify the principal objective of the proposed framework, the integration of heterogeneous telemetry within the execution-regime space, the scope and interpretation of the experimental validation, and the relationship between our approach and existing production observability infrastructures. We also appreciate the Reviewer’s attention to the completeness and relevance of the literature supporting the study. We have carefully considered each comment and provide our detailed responses below.

1. What is the main objective of the proposed invariant-guided geometric framework for execution-regime analysis?

**Response**: We thank the Reviewer for this question. The main objective of the proposed framework is to transform heterogeneous operational telemetry into an explicit, invariant-guided geometric space of execution regimes in which persistent behavioural deviations can be identified as structural regime transitions under limited supervision. Rather than treating individual log events, KPI values, or latent embeddings as the primary analytical objects, the framework models execution regimes themselves through feature mappings, weighted pseudometrics, invariant-guided regularisation, and geometric relationships. This makes it possible to analyse anomalous behaviour as a structured change in system execution while preserving explicit regime-level semantics and compatibility with weakly supervised operational settings. This objective is stated explicitly in the Introduction and operationalised mathematically in Section 2.

2: How does the proposed framework integrate heterogeneous telemetry data, such as logs and KPI observations, into a unified execution-regime space?

**Response**: We thank the Reviewer for this question. The framework integrates heterogeneous telemetry at the level of structural regime representation rather than by forcing raw logs and KPI signals into a common native format. Each telemetry modality is first transformed through modality-specific deterministic descriptors. For logs, these descriptors capture event-template occurrence, normalised template frequency, sequential variation, and template identity; for KPI observations, they capture normalised level, inter-window deviation, local temporal slope, and within-window variability.

Directed structural differences are then formed between consecutive regime representations, after which the resulting descriptor values are normalised and assembled into a fixed-dimensional regime vector $\mu_{\delta_{r}}$. These vectors are embedded into the same weighted geometric framework through the pseudometric $d_{w}$, geometric regularisation, and invariant-guided constraints. Thus, heterogeneous telemetry is unified not by homogenising the raw observations themselves, but by mapping modality-specific structural changes into a common execution-regime representation on which the same geometric and invariant-based analysis can operate.

In the present experiments, the HDFS benchmark instantiates the log branch of the framework, whereas the AIOps benchmark instantiates the KPI branch; unavailable modalities are not synthetically imputed. This preserves the integrity of each dataset while demonstrating that the same mathematical regime-space formalism can be operationalised across distinct telemetry types.

3. What datasets and baseline methods were used to evaluate the performance of the proposed framework, and what improvements were observed?

**Response**: We thank the Reviewer for this question. The framework was evaluated on two complementary operational telemetry benchmarks: the 2018 AIOps Challenge dataset, comprising 29 labelled KPI time series, and the HDFS dataset from LogHub, comprising more than 11 million log messages organised into labelled execution sessions. These datasets represent continuous KPI dynamics and discrete log-event structure, respectively.

In the principal weak-supervision experiments, the proposed framework was compared with ADS on AIOps and with DeepLog and PLELog on HDFS. Under full-pipeline evaluation, it improved F1 over the strongest corresponding baseline by 0.105 and 0.055 on AIOps at 1% and 5% supervision, and by 0.071 and 0.041 on HDFS, corresponding to relative improvements of 16.3%, 7.4%, 10.9%, and 5.7%, respectively. All four differences remained statistically significant after Holm correction.

The revised manuscript additionally reports a controlled HDFS comparison with LogGPT, LogLLM, and Llama3-ReFT. Under this common labelled session-level protocol, the proposed framework achieved an F1 score of 0.921 ± 0.015, exceeding LogGPT by 0.017 while trailing LogLLM and Llama3-ReFT by 0.074 and 0.065, respectively. Thus, the experiments show consistent gains over the methodologically closest baselines under scarce supervision, while the additional comparison also establishes the predictive performance boundary relative to recent high-capacity language-model-based detectors.

4. How does the proposed framework complement existing production observability infrastructures while providing interpretable execution-regime representations?

**Response**: We thank the Reviewer for this question. The proposed framework is designed to complement, rather than replace, existing production observability infrastructures. It assumes that logs, KPI measurements, and other operational telemetry have already been acquired through the existing monitoring stack and introduces an analytical layer above these mechanisms. Accordingly, functions such as telemetry collection, visualisation, alert routing, orchestration, infrastructure control, and incident-response automation remain within the existing production infrastructure.

The framework adds a different capability by transforming the collected telemetry into explicit execution-regime representations. Instead of returning only isolated anomaly flags or latent detection scores, it represents regimes through defined feature mappings, weighted pseudometrics, invariant relationships, neighbourhood structure, and geometric organisation. This makes stable regimes, transitional regions, and structurally anomalous regime shifts directly analysable within the resulting regime space.

Thus, the complementarity is functional: existing observability infrastructure provides and operationalises telemetry, whereas the proposed framework provides an interpretable mathematical layer for analysing persistent structural changes in the execution behaviour represented by that telemetry.

5. Ensure that all sources and data are properly cited throughout the manuscript. For instance, the following papers could be helpful in improving the literature…

**Response**: We thank the Reviewer for emphasising the importance of complete and appropriate source attribution. We have carefully re-checked the manuscript to ensure that the datasets, baseline methods, methodological components, and externally derived claims are supported by the corresponding references.

We also examined the publications suggested by the Reviewer. These studies primarily concern fractional-order modelling of magnetohydrodynamic and non-Newtonian fluid flows, nanofluid heat and mass transfer, thermal radiation, and related fractional differential operators. Their research objects and methodological objectives differ substantially from those of the present study, which focuses on anomaly detection from production telemetry, invariant-guided geometric modelling of execution regimes, and weakly supervised analysis of logs and KPI observations.

For this reason, we did not incorporate these references, as they do not directly support the theoretical, methodological, or empirical claims made in the manuscript. We instead retained and verified references that are directly relevant to production observability, log and KPI anomaly detection, representation learning, weak supervision, and execution-regime analysis.
