# Supplementary material for: An invariant-guided model for automated detection of anomalous execution regimes from production telemetry
Source: PLoS One. 2026 Nov 12;21(11):e0362583. doi: 10.1371/journal.pone.0362583 (PMC13759071; doi:10.1371/journal.pone.0362583)
Supplement: Peer review history

# Peer review: Decision Letter 0

**DOI:** 10.1371/journal.pone.0362583.r001

**Authors:** Shrisha Rao

29 Jun 2026

PONE-D-26-08960

A Model for Automated Detection of Security-Relevant Errors Based on Structural Analysis of Program Execution Regimes

PLOS One

Dear Dr. Kovtun,

Thank you for submitting your manuscript to PLOS ONE. After careful consideration, we have decided that your manuscript does not meet our criteria for publication and must therefore be rejected.

Specifically:

==============================

**ACADEMIC EDITOR:**

The paper is written in an unacceptably informal and conversational register, falls well below the standard of academic English, and devotes excessive space to elementary material while leaving its core formal constructs -- including the local feature functions $l_i$ and $m_j$, the weighting functions $\alpha_k$ and $\beta_k$, and the invariant violation functionals $\psi_m$ -- entirely unspecified in operational terms, rendering the experiments irreproducible and the claimed contributions unverifiable. The experimental comparisons are restricted to outdated baselines, evaluated on aging and unrepresentative datasets under an offline protocol that is inconsistent with the production monitoring use case the paper purports to address, and the reported performance improvements are modest and unsupported by any test of statistical significance. Most fundamentally, the paper misrepresents the state of cloud computing practice, ignores the sophisticated observability, scheduling, and DCIM tooling already deployed in production environments, and conflates performance degradation with security incidents in a manner that renders its central security claims technically incoherent.

==============================

I am sorry that we cannot be more positive on this occasion, but hope that you appreciate the reasons for this decision.

Kind regards,

Shrisha Rao, Ph.D.

Academic Editor

PLOS One

**Additional Editor Comments:**

This is not written to the standards expected at PLOS One. Kindly revise the paper with proper use of language as expected from a scientific paper. Use of terms like "They're", "we're" and so on are not befitting of scientific writing at least not in PLOS One.

[Note: HTML markup is below. Please do not edit.]

Reviewers' comments:

Reviewer's Responses to Questions

**Comments to the Author**

1. Is the manuscript technically sound, and do the data support the conclusions?

Reviewer #1: Yes

**********

2. Has the statistical analysis been performed appropriately and rigorously?

Reviewer #1: Yes

**********

3. Have the authors made all data underlying the findings in their manuscript fully available?

The PLOS Data policy

Reviewer #1: Yes

**********

4. Is the manuscript presented in an intelligible fashion and written in standard English?

Reviewer #1: Yes

**********

Reviewer #1: This is an excellent, well-conceived manuscript that makes a meaningful contribution to the field of cloud-native observability and automated anomaly detection. Your shift towards analyzing "execution regimes" geometrically is a highly effective way to handle the noisy, heterogeneous telemetry found in production environments.

Your experimental protocol is highly commendable. By evaluating the model under "baseline" (5%) and "strict" (1%) supervision limits, you successfully proved that your geometric approach is significantly more robust to label scarcity than state-of-the-art alternatives like DeepLog and PLELog, maintaining tight statistical stability and high precision.

The primary limitation of your study—that it currently relies on an offline, static analysis protocol without streaming data updates—is transparently acknowledged in your conclusion. To strengthen the paper further, you might briefly expand in your discussion on the specific computational challenges that will need to be overcome to adapt this weighted pseudometric and clustering optimization into an online, real-time streaming environment in your future work.

**********

what does this mean?). If published, this will include your full peer review and any attached files.

If you choose “no”, your identity will remain anonymous but your review may still be made public.

**Do you want your identity to be public for this peer review?**  For information about this choice, including consent withdrawal, please see our Privacy Policy

Reviewer #1: No

**********

[NOTE: If reviewer comments were submitted as an attachment file, they will be attached to this email and accessible via the submission site. Please log into your account, locate the manuscript record, and check for the action link "View Attachments". If this link does not appear, there are no attachment files.]

- - - - -

For journal use only: PONEDEC3

# Author comment: Author response to Decision Letter 1

**DOI:** 10.1371/journal.pone.0362583.r002

7 Jul 2026

A detailed point-by-point response to all comments raised by the Academic Editor and reviewers is provided in the uploaded "Response to Reviewers" document. The manuscript has been revised accordingly, and all requested files have been included with this resubmission.

Attachment Submitted filename: Response.pdf

# Peer review: Decision Letter 1

**DOI:** 10.1371/journal.pone.0362583.r003

**Authors:** Issa Atoum

9 Sep 2026

Dear Dr. Kovtun,

Thank you for submitting your manuscript to PLOS One. After careful consideration, we feel that it has merit but does not fully meet PLOS One’s publication criteria as it currently stands. Therefore, we invite you to submit a revised version of the manuscript that addresses the points raised during the review process.

==============================

Please submit your revised manuscript by Oct 23 2026 11:59PM. If you will need more time than this to complete your revisions, please reply to this message or contact the journal office at plosone@plos.org. When you're ready to submit your revision, log on to https://www.editorialmanager.com/pone/ and select the 'Submissions Needing Revision' folder to locate your manuscript file.

- A letter that responds to each point raised by the academic editor and reviewer(s). You should upload this letter as a separate file labeled 'Response to Reviewers'.
- A marked-up copy of your manuscript that highlights changes made to the original version. You should upload this as a separate file labeled 'Revised Manuscript with Track Changes'.
- An unmarked version of your revised paper without tracked changes. You should upload this as a separate file labeled 'Manuscript'.

If you would like to make changes to your financial disclosure, please include your updated statement in your cover letter. Guidelines for resubmitting your figure files are available below the reviewer comments at the end of this letter.

If applicable, we recommend that you deposit your laboratory protocols in protocols.io to enhance the reproducibility of your results. Protocols.io assigns your protocol its own identifier (DOI) so that it can be cited independently in the future. For instructions see: https://journals.plos.org/plosone/s/submission-guidelines#loc-laboratory-protocols. Additionally, PLOS One offers an option for publishing peer-reviewed Lab Protocol articles, which describe protocols hosted on protocols.io. Read more information on sharing protocols at https://plos.org/protocols?utm_medium=editorial-email&utm_source=authorletters&utm_campaign=protocols.

As the corresponding author, your ORCID iD is verified in the submission system and will appear in the published article. PLOS supports the use of ORCID, and we encourage all coauthors to register for an ORCID iD and use it as well. Please encourage your coauthors to verify their ORCID iD within the submission system before final acceptance, as unverified ORCID iDs will not appear in the published article. *Only*  the individual author can complete the verification step; PLOS staff *cannot*  verify ORCID iDs on behalf of authors.

We look forward to receiving your revised manuscript.

Kind regards,

Issa Atoum

Academic Editor

PLOS One

Journal Requirements:

When submitting your revision, we need you to address these additional requirements.

1. Please ensure that your manuscript meets PLOS ONE's style requirements, including those for file naming. The PLOS ONE style templates can be found at

https://journals.plos.org/plosone/s/file?id=wjVg/PLOSOne_formatting_sample_main_body.pdf and

https://journals.plos.org/plosone/s/file?id=ba62/PLOSOne_formatting_sample_title_authors_affiliations.pdf.

2. Please note that PLOS One has specific guidelines on code sharing for submissions in which author-generated code underpins the findings in the manuscript. In these cases, we expect all author-generated code to be made available without restrictions upon publication of the work. Please review our guidelines at https://journals.plos.org/plosone/s/materials-and-software-sharing#loc-sharing-code and ensure that your code is shared in a way that follows best practice and facilitates reproducibility and reuse.

3. Please provide a complete Data Availability Statement in the submission form, ensuring you include all necessary access information or a reason for why you are unable to make your data freely accessible. If your research concerns only data provided within your submission, please write "All data are in the manuscript and/or supporting information files" as your Data Availability Statement.

If the reviewer comments include a recommendation to cite specific previously published works, please review and evaluate these publications to determine whether they are relevant and should be cited. There is no requirement to cite these works unless the editor has indicated otherwise.

Additional Editor Comments (if provided):

Comments from the Editorial Office: Please note there is no expectation to include any of the references requested by reviewer 3. You should assess their relevance to your work.

[Note: HTML markup is below. Please do not edit.]

Reviewers' comments:

Reviewer's Responses to Questions

**Comments to the Author**

Reviewer #2: All comments have been addressed

Reviewer #3: All comments have been addressed

**********

2. Is the manuscript technically sound, and do the data support the conclusions?

Reviewer #2: Yes

Reviewer #3: Partly

**********

3. Has the statistical analysis been performed appropriately and rigorously?

Reviewer #2: Yes

Reviewer #3: Yes

**********

4. Have the authors made all data underlying the findings in their manuscript fully available?

The PLOS Data policy

Reviewer #2: Yes

Reviewer #3: No

**********

5. Is the manuscript presented in an intelligible fashion and written in standard English?

Reviewer #2: Yes

Reviewer #3: Yes

**********

Reviewer #2: The manuscript should better quantify the computational complexity, runtime overhead, and scalability of the proposed framework, particularly for large-scale cloud-native production environments.

Although the mathematical framework is rigorous, several implementation details (e.g., feature construction, weight calibration, and hyperparameter selection) should be described more explicitly to improve reproducibility.

The experimental evaluation would be strengthened by including additional recent deep learning and foundation-model-based anomaly detection methods as baselines.

The manuscript should discuss the limitations of the proposed approach more explicitly, particularly regarding online adaptation, concept drift, streaming telemetry, and real-time deployment.

Several theoretical sections are unnecessarily long and mathematically dense; condensing these sections while emphasizing the practical workflow would improve readability.

Additional validation on more diverse industrial datasets and production environments would provide stronger evidence of the framework's generalizability and practical applicability

Reviewer #3: 1. What is the main objective of the proposed invariant-guided geometric framework for execution-regime analysis?

2. How does the proposed framework integrate heterogeneous telemetry data, such as logs and KPI observations, into a unified execution-regime space?

3. What datasets and baseline methods were used to evaluate the performance of the proposed framework, and what improvements were observed?

4. How does the proposed framework complement existing production observability infrastructures while providing interpretable execution-regime representations?

5. Ensure that all sources and data are properly cited throughout the manuscript. For instance, the following papers could be helpful in improving the literature;

1)Fractional Analysis of Magnetohydrodynamics Maxwell Flow Over an Inclined Plate with the Effect of Thermal Radiation; 2)Analysis of heat and mass transfer on nanofluid: An application of hybrid fractal-fractional derivative; 3)A comparative study of heat absorption and chemical reaction on MHD flow with fractional derivatives; 4)Effect of Thermal Radiation on Fractional MHD Casson Flow with the Help of Fractional Operator; 5)Heat transfer analysis of Walter's-B flow over a vertical plate by using fractional operator; 6)Fractional modeling of bioconvection in Jeffrey nanofluids with gyrotactic organisms; 7)Enhanced heat transfer in nanofluid flow via prabhakar fractional models for solar thermal systems; 8)Slip and thermal effects on MHD fractional Jeffrey nanofluid flow; 9)Impact of thermal radiation on fractional viscoelastic nanofluid flow with joint heat mass transfer using a neural network based approach;10)Fractional Analysis of Unsteady Casson Flow in the Presence of Riga Plate

**********

what does this mean?). If published, this will include your full peer review and any attached files.

If you choose “no”, your identity will remain anonymous but your review may still be made public.

**Do you want your identity to be public for this peer review?**  For information about this choice, including consent withdrawal, please see our Privacy Policy

Reviewer #2: No

Reviewer #3: No

**********

[NOTE: If reviewer comments were submitted as an attachment file, they will be attached to this email and accessible via the submission site. Please log into your account, locate the manuscript record, and check for the action link "View Attachments". If this link does not appear, there are no attachment files.]

To ensure your figures meet our technical requirements, please review our figure guidelines: https://journals.plos.org/plosone/s/figures

You may also use PLOS’s free figure tool, NAAS, to help you prepare publication quality figures: https://journals.plos.org/plosone/s/figures#loc-tools-for-figure-preparation.

NAAS will assess whether your figures meet our technical requirements by comparing each figure against our figure specifications.

# Author comment: Author response to Decision Letter 2

**DOI:** 10.1371/journal.pone.0362583.r004

10 Sep 2026

We sincerely thank the Academic Editor and the Reviewers for their careful assessment and constructive comments. We have addressed all comments in the revised manuscript. A detailed point-by-point response to the Academic Editor and Reviewers has been uploaded as a separate document entitled “Responses to the Academic Editor and Reviewers”. Please refer to the uploaded response file for our complete responses and a detailed description of the corresponding revisions.

Attachment Submitted filename: Responses.doc

# Peer review: Decision Letter 2

**DOI:** 10.1371/journal.pone.0362583.r005

**Authors:** Issa Atoum

6 Oct 2026

Dear Dr. Kovtun,

Thank you for submitting your manuscript to PLOS One. After careful consideration, we feel that it has merit but does not fully meet PLOS One’s publication criteria as it currently stands. Therefore, we invite you to submit a revised version of the manuscript that addresses the points raised during the review process.

Please submit your revised manuscript by Nov 20 2026 11:59PM. If you will need more time than this to complete your revisions, please reply to this message or contact the journal office at plosone@plos.org. When you're ready to submit your revision, log on to https://www.editorialmanager.com/pone/ and select the 'Submissions Needing Revision' folder to locate your manuscript file.

- A letter that responds to each point raised by the academic editor and reviewer(s). You should upload this letter as a separate file labeled 'Response to Reviewers'.
- A marked-up copy of your manuscript that highlights changes made to the original version. You should upload this as a separate file labeled 'Revised Manuscript with Track Changes'.
- An unmarked version of your revised paper without tracked changes. You should upload this as a separate file labeled 'Manuscript'.

If you would like to make changes to your financial disclosure, please include your updated statement in your cover letter. Guidelines for resubmitting your figure files are available below the reviewer comments at the end of this letter.

If applicable, we recommend that you deposit your laboratory protocols in protocols.io to enhance the reproducibility of your results. Protocols.io assigns your protocol its own identifier (DOI) so that it can be cited independently in the future. For instructions see: https://journals.plos.org/plosone/s/submission-guidelines#loc-laboratory-protocols. Additionally, PLOS One offers an option for publishing peer-reviewed Lab Protocol articles, which describe protocols hosted on protocols.io. Read more information on sharing protocols at https://plos.org/protocols?utm_medium=editorial-email&utm_source=authorletters&utm_campaign=protocols.

As the corresponding author, your ORCID iD is verified in the submission system and will appear in the published article. PLOS supports the use of ORCID, and we encourage all coauthors to register for an ORCID iD and use it as well. Please encourage your coauthors to verify their ORCID iD within the submission system before final acceptance, as unverified ORCID iDs will not appear in the published article. *Only*  the individual author can complete the verification step; PLOS staff *cannot*  verify ORCID iDs on behalf of authors.

We look forward to receiving your revised manuscript.

Kind regards,

Issa Atoum

Academic Editor

PLOS One

Journal Requirements:

If the reviewer comments include a recommendation to cite specific previously published works, please review and evaluate these publications to determine whether they are relevant and should be cited. There is no requirement to cite these works unless the editor has indicated otherwise.

Please review your reference list to ensure that it is complete and correct. If you have cited papers that have been retracted, please include the rationale for doing so in the manuscript text, or remove these references and replace them with relevant current references. Any changes to the reference list should be mentioned in the rebuttal letter that accompanies your revised manuscript. If you need to cite a retracted article, indicate the article’s retracted status in the References list and also include a citation and full reference for the retraction notice.

Additional Editor Comments :

**Comments from the Editorial Office:**  The reviewer has declared the use of AI assistance in their review. They have declared the use of AI assistant (Claude, Anthropic) to help analyse the manuscript, check the consistency of the reported numbers and figures, and draft and edit the review text. They have checked the comments and take responsibility for their content. Your Academic Editor has also assessed the relevance of this review and is proceeding with the decision. If you have any concerns about the use of AI and the comments left at this peer review round, please contact the journal office.

[Note: HTML markup is below. Please do not edit.]

Reviewers' comments:

Reviewer's Responses to Questions

**Comments to the Author**

Reviewer #4: (No Response)

**********

2. Is the manuscript technically sound, and do the data support the conclusions?

Reviewer #4: Partly

**********

3. Has the statistical analysis been performed appropriately and rigorously?

Reviewer #4: No

**********

4. Have the authors made all data underlying the findings in their manuscript fully available?

The PLOS Data policy

Reviewer #4: No

**********

5. Is the manuscript presented in an intelligible fashion and written in standard English?

Reviewer #4: Yes

**********

Reviewer #4: The revision is clearly more concrete than what one usually sees at this stage. The implementation details in Section 3 are much easier to follow, the complexity discussion is useful, and the new HDFS comparison in Table 3 is a good addition. A shared Block-ID split with a threshold chosen only on validation data is the right way to run that experiment. I also appreciated the honesty about LogLLM and Llama3-ReFT doing better.

I think the paper is close. There are, however, some reporting problems in the weak-supervision results that need to be sorted out, plus a few places where the claims go further than the experiments. Most of these should be fixable by correcting, clarifying or adding results the authors likely already have.

MAIN POINTS TO ADDRESS

1. Some of the HDFS numbers don't seem to fit the protocol as described. The paper says the top 1% (or 5%) of scores are flagged, and by the authors' own counts about 2.9% of HDFS sessions are anomalous. If only 1% is flagged, recall can't exceed about 0.34, which caps F1 at roughly 0.51. The text reports about 0.73. A similar back-of-the-envelope check on AIOps gives two different implied anomaly rates for the same data, about 5.4% and 1.3%. I suspect the evaluation set or threshold is different from what's written. Please state exactly which set the metrics come from, give its size and anomaly rate, and include confusion matrices. Please also clarify whether "1%" and "5%" mean the share of labelled anomalies, the flagging threshold, or both.

2. Figure 4 doesn't match the text or Table 2:

- In Fig. 4b, precision goes up as supervision increases. The text says the opposite.

- The baseline F1 values at 1% in Fig. 4a (about 0.73–0.76) are well above those in Table 2 (0.646 and 0.651).

- It's unclear which dataset is shown. ADS, DeepLog and PLELog share one set of axes even though they apply to different data.

- There is a 2% point that the protocol doesn't mention.

Please regenerate the figure per dataset, from the same runs as Table 2.

3. Please describe the evaluation set for the weak-supervision experiment. The weak labels enter model selection through the label-consistency term, and the bootstrap is described as resampling "the test execution regimes." However, I couldn't find where that test set is defined, or whether the labelled regimes are excluded from scoring. It would also help to note that the 20 repetitions vary only the k-means initialization. Ideally, add a few runs with different weak-label subsets.

4. The title and parts of the text claim security relevance that the experiments don't show. Both datasets are labelled for operational problems: failed HDFS blocks and operator-marked KPI anomalies. Section 2.1 itself says invariant violations aren't evidence of a security incident, which I agree with. I'd suggest adjusting the title and phrases such as "fundamentally important for information security tasks" to reflect operational anomaly detection. Alternatively, the authors could clearly mark security application as future work.

5. The invariants need to be specified. I found the names of three invariant families per dataset, but not the functionals ϕ_m themselves, nor which data the "nominal" reference values are estimated from. Please give the formulas. Please also report the selected k, λ1 and λ2 for each setting. Without them, readers can't tell whether the invariant term was actually active (λ2 > 0).

6. The Introduction and Conclusions both refer to an ablation study, including its outcome, but I couldn't find it in the manuscript. Please include those results. Removing R_inv, R_geom and the learned weights one at a time would be the most informative.

7. The significance testing should be revised. A Wilcoxon test on bootstrap replicates doesn't give a meaningful p-value, because the replicates aren't independent. In Table 2, non-overlapping AIOps intervals sit next to Cliff's δ values of 0.64 and 0.50, which doesn't seem consistent. Reporting a paired bootstrap interval on the F1 difference, or McNemar's test on per-regime decisions, would be more appropriate.

8. Please describe how DeepLog, PLELog and ADS were run, tuned, and adapted to the 1%/5% labels and the quantile threshold. Please also comment on why their HDFS F1 values are well below those in their original papers. Invariant Mining (Lou et al., USENIX ATC 2010) is the classic invariant-based HDFS detector and very close to this paper's idea, so it should at least be cited and discussed. Recall, false-positive rate and AUROC/AUPRC would also help readers judge the comparison.

9. Please state which data and sample Figures 1–3 show, and how they were produced.

- Fig. 3 is presented as the matrix of d_w, but its diagonal isn't zero and it doesn't appear symmetric, as a pseudometric should be. It also shows two equal-sized blocks, which doesn't fit a roughly 3% anomaly rate.

- The Fig. 2 legend mentions invariant-consistency encoding that I couldn't see in the plot.

- Statements such as "two thirds" and "a factor of one and a half" should be backed by the calculation.

10. The paper emphasizes combining logs and metrics, but each experiment uses only one modality (p. 30). Please tone down this claim, or clearly mark joint evaluation as future work.

11. A few pipeline details need clarifying:

- For HDFS, how are the independent block sessions ordered to form "transitions," and how is a transition labelled when one side is normal and the other anomalous?

- How are KPI windows labelled?

- How are zero difference vectors handled in the angular similarity?

- How does the method avoid giving low scores to anomalies that form their own compact cluster?

12. Please share the code and derived data: processed features, labels, weak-label subsets, splits, selected hyperparameters and per-run results. Please also report runtime and hardware. Without these the tables and figures can't be checked, which the PLOS data policy expects.

MINOR COMMENTS

1. On p. 33, DeepLog, PLELog and ADS are cited as [16], [26] and [27]. Those are a stock-prediction paper, LAnoBERT and LogFormer. I think [21], [22] and [36] were intended.

2. LogLLM and LogADReFT (Table 3) aren't in the reference list.

3. Some references don't match what they're cited for: [9] (educational learning environments), [15] (a social-science methods handbook) and [16] (stock prices).

4. The KPI data is described as "multivariate" on p. 26 and "univariate" on p. 29.

5. Please give the sampling interval of each KPI series.

6. There are leftover LaTeX fragments: "\(k\)", "\(w\)", "(\delta)" and "\The operational configuration".

7. "The overall optimisation scheme (10)" should probably refer to Eq. (11).

8. The HDFS session count is 575,062 on p. 26 and 575,061 on p. 42.

9. Fig. 1 has no axis labels, and Fig. 2 doesn't say which embedding method was used.

10. Table 1 rates interpretability as "high," but interpretability isn't evaluated.

11. The Section 2.2 heading ("Errors") doesn't match the title's framing.

12. "Static analysis" means something else to most software engineers. "Offline batch evaluation" would be clearer.

13. The notation switches between R_inv(M) and R_inv(Φ), and Eq. (8) isn't defined when all v_m are zero.

14. The keyword "automated software testing" doesn't really fit the paper.

15. A sentence on privacy would be good, since production logs often contain personal data.

QUESTIONS FOR THE AUTHORS

1. Which set are the Table 2 and Fig. 4 metrics computed on, and what is its anomaly rate?

2. Do "1%" and "5%" mean the labelled fraction, the threshold, or both?

3. Which k, λ1 and λ2 were selected? Was λ2 above zero?

4. Where are the ablation results mentioned in the Conclusions?

5. Why doesn't Fig. 3 have a zero diagonal, and what data does it show?

6. How were Cliff's δ values of 0.50–0.64 obtained when the intervals don't overlap?

**********

what does this mean?). If published, this will include your full peer review and any attached files.

If you choose “no”, your identity will remain anonymous but your review may still be made public.

**Do you want your identity to be public for this peer review?**  For information about this choice, including consent withdrawal, please see our Privacy Policy

Reviewer #4: **Yes:**  Tetiana Afanasieva

**********

[NOTE: If reviewer comments were submitted as an attachment file, they will be attached to this email and accessible via the submission site. Please log into your account, locate the manuscript record, and check for the action link "View Attachments". If this link does not appear, there are no attachment files.]

To ensure your figures meet our technical requirements, please review our figure guidelines: https://journals.plos.org/plosone/s/figures

You may also use PLOS’s free figure tool, NAAS, to help you prepare publication quality figures: https://journals.plos.org/plosone/s/figures#loc-tools-for-figure-preparation.

NAAS will assess whether your figures meet our technical requirements by comparing each figure against our figure specifications.

# Author comment: Author response to Decision Letter 3

**DOI:** 10.1371/journal.pone.0362583.r006

7 Oct 2026

We thank the Academic Editor and Reviewer #4 for their careful assessment and constructive comments. Our detailed point-by-point responses to the Academic Editor, journal requirements, and all reviewer comments and questions are provided in the PDF uploaded as “Response to Reviewers”. The corresponding revisions are incorporated into the revised manuscript and identified in the marked version.

Attachment Submitted filename: Responses.pdf

# Peer review: Decision Letter 3

**DOI:** 10.1371/journal.pone.0362583.r007

**Authors:** Issa Atoum

28 Oct 2026

An Invariant-Guided Model for Automated Detection of Anomalous Execution Regimes from Production Telemetry

PONE-D-26-08960R3

Dear Dr. Kovtun,

We’re pleased to inform you that your manuscript has been judged scientifically suitable for publication and will be formally accepted for publication once it meets all outstanding technical requirements.

Within one week, you’ll receive an e-mail detailing the required amendments. When these have been addressed, you’ll receive a formal acceptance letter and your manuscript will be scheduled for publication.

An invoice will be generated when your article is formally accepted. Please note, if your institution has a publishing partnership with PLOS and your article meets the relevant criteria, all or part of your publication costs will be covered. Please make sure your user information is up-to-date by logging into Editorial Manager at Editorial Manager® and clicking the ‘Update My Information' link at the top of the page. For questions related to billing, please contact billing support.

If your institution or institutions have a press office, please notify them about your upcoming paper to help maximize its impact. If they’ll be preparing press materials, please inform our press team as soon as possible -- no later than 48 hours after receiving the formal acceptance. Your manuscript will remain under strict press embargo until 2 pm Eastern Time on the date of publication. For more information, please contact onepress@plos.org.

Kind regards,

Issa Atoum

Academic Editor

PLOS One

Additional Editor Comments (optional):

Reviewers' comments:

Reviewer's Responses to Questions

**Comments to the Author**

Reviewer #4: All comments have been addressed

**********

2. Is the manuscript technically sound, and do the data support the conclusions?

Reviewer #4: Yes

**********

3. Has the statistical analysis been performed appropriately and rigorously?

Reviewer #4: Yes

**********

4. Have the authors made all data underlying the findings in their manuscript fully available?

The PLOS Data policy

Reviewer #4: Yes

**********

5. Is the manuscript presented in an intelligible fashion and written in standard English?

Reviewer #4: Yes

**********

Reviewer #4: I thank the authors for their careful and thorough response to my previous comments. The revised manuscript is substantially improved, and I recommend it for publication.

Main claims and significance

The paper proposes a framework that represents execution regimes from operational telemetry as explicit mathematical objects. The framework combines feature mappings, a weighted pseudometric, invariant-based and geometric regularisation, and weakly supervised calibration of the metric weights. Anomalous regimes are detected through angular similarity to learned prototypes. The method is evaluated separately on HDFS logs and on the 2018 AIOps KPI dataset with 1% and 5% development-label budgets. On both datasets it shows consistent F1 improvements over DeepLog, PLELog and ADS. Under full supervision on HDFS, it occupies an intermediate position between LogGPT and recent LLM-based detectors.

The contribution is relevant to AIOps and log-based anomaly detection. Label scarcity is a practical constraint in these fields, and inspectable quantities (invariant violations, feature weights, distances, prototypes) are valuable to operators. The claims are now appropriately scoped. The evaluation concerns offline operational anomaly detection, not security incidents. Each telemetry modality is evaluated on its own, and joint log–metric evaluation and streaming deployment are explicitly identified as future work.

Placement in the literature

The literature review covers rule-based, statistical, sequence, transformer/LLM, graph-based and KPI-oriented approaches. It now cites and discusses Invariant Mining (Lou et al.) as the direct antecedent of invariant-based HDFS detection, and it states the contribution relative to that work fairly. The baseline citations (DeepLog, PLELog, ADS, LogLLM, LogADReFT) have been corrected. The authors also acknowledge openly that the baselines' published HDFS results were obtained under different protocols, and that the proposed method trails LogLLM and Llama3-ReFT under full supervision. I consider this a fair and balanced treatment.

Support for the claims

The evaluation protocol is now clearly specified. The annotation budgets are separated from the decision threshold, and the test sets are defined with their sizes and prevalences. Thresholds are calibrated on development data only, and confusion matrices are reported. I checked the confusion matrices against the reported precision, recall, F1 and alert fractions, and they are consistent. The supporting analyses are appropriate:

- paired moving-block bootstrap intervals with familywise adjustment;

- separate experiments on initialisation and weak-label-subset sensitivity;

- a component ablation (Table 4);

- complementary metrics: recall, FPR, AUROC and AUPRC (Table 2.1).

Together these support the conclusions within the stated scope.

Reproducibility, protocols and software

The methodology is described in sufficient detail to reproduce the experiments. This includes the invariant functionals, nominal-reference estimation, weight-calibration algorithm, hyperparameter grids, selected configurations, seeds, partitioning and the baseline adaptations. Both datasets are public, and a Python reference implementation is freely available on Zenodo (https://doi.org/10.5281/zenodo.23218142) under GPL-3.0. Optionally, I suggest providing the following as Supporting Information to make independent checks easier: the development/test manifests, the identities of the disclosed weak labels, and the per-run results. This is a suggestion, not a condition for acceptance.

Reporting guidelines

This is a computational study on public benchmark data. Reporting guidelines such as CONSORT, MIAME or STROBE do not apply.

Organisation and clarity

The manuscript is well organised, and the English is clear. The mathematical sections are dense, but the operational descriptions in Section 3 make the method accessible to readers outside the immediate specialty.

Status of my previous comments

1. HDFS/AIOps numbers vs. protocol: resolved. The budgets are distinguished from the threshold, the test sets are defined, and confusion matrices are added.

2. Figure 4 inconsistencies: resolved. The figure has been regenerated per dataset and matches Table 2.

3. Weak-supervision evaluation set: resolved. The test set is defined, and a weak-label-subset experiment has been added.

4. Security framing: resolved. The title and text have been revised, and security evaluation is named as future work.

5. Invariant specification and selected k, lambda1, lambda2: resolved.

6. Missing ablation: resolved (Table 4).

7. Significance testing: resolved. Paired bootstrap intervals on F1 differences are now used.

8. Baseline details, the gap to published results, Invariant Mining, and additional metrics: resolved.

9. Figures 1–3: resolved. Samples and construction are documented, and Figure 3 is a valid pseudometric matrix.

10. Multimodal claim: resolved. Joint evaluation is named as future work.

11. Pipeline details: resolved.

12. Code and runtime environment: resolved. The implementation is archived, and the hardware is reported.

All minor comments have also been addressed.

**********

what does this mean?). If published, this will include your full peer review and any attached files.

If you choose “no”, your identity will remain anonymous but your review may still be made public.

**Do you want your identity to be public for this peer review?**  For information about this choice, including consent withdrawal, please see our Privacy Policy

Reviewer #4: **Yes:**  Tetiana Afanasieva

**********

# Editor report: Acceptance letter

**DOI:** 10.1371/journal.pone.0362583.r008

**Authors:** Issa Atoum

PONE-D-26-08960R3

PLOS One

Dear Dr. Kovtun,

I'm pleased to inform you that your manuscript has been deemed suitable for publication in PLOS One. Congratulations! Your manuscript is now being handed over to our production team.

At this stage, our production department will prepare your paper for publication. This includes ensuring the following:

* All references, tables, and figures are properly cited

* All relevant supporting information is included in the manuscript submission,

* There are no issues that prevent the paper from being properly typeset

You will receive further instructions from the production team, including instructions on how to review your proof when it is ready. Please keep in mind that we are working through a large volume of accepted articles, so please give us a few days to review your paper and let you know the next and final steps.

Lastly, if your institution or institutions have a press office, please let them know about your upcoming paper now to help maximize its impact. If they'll be preparing press materials, please inform our press team within the next 48 hours. Your manuscript will remain under strict press embargo until 2 pm Eastern Time on the date of publication. For more information, please contact onepress@plos.org.

You will receive an invoice from PLOS for your publication fee after your manuscript has reached the completed accept phase. If you receive an email requesting payment before acceptance or for any other service, this may be a phishing scheme. Learn how to identify phishing emails and protect your accounts at https://explore.plos.org/phishing.

If we can help with anything else, please email us at customercare@plos.org.

Thank you for submitting your work to PLOS One and supporting open access.

Kind regards,

PLOS One Editorial Office Staff

on behalf of

Dr. Issa Atoum

Academic Editor

PLOS One
